# Circulating Polyunsaturated Fatty Acids and COVID-19: A Prospective Cohort Study and Mendelian Randomization Analysis

**DOI:** 10.3389/fmed.2022.923746

**Published:** 2022-06-16

**Authors:** Yitang Sun, Radhika Chatterjee, Akash Ronanki, Kaixiong Ye

**Affiliations:** ^1^Department of Genetics, Franklin College of Arts and Sciences, University of Georgia, Athens, GA, United States; ^2^Institute of Bioinformatics, University of Georgia, Athens, GA, United States

**Keywords:** COVID-19, polyunsaturated fatty acid, Mendelian randomization, prospective cohort, docosapentaenoic acid, arachidonic acid

## Abstract

Higher circulating polyunsaturated fatty acids (PUFAs), especially omega-3 fatty acids, have been linked to a better prognosis in patients of coronavirus disease 2019 (COVID-19). However, the effects and causality of pre-infection PUFA levels remain unclear. This study aimed to investigate the observational and causal associations of circulating PUFAs with COVID-19 susceptibility and severity. We first performed a prospective cohort study in UK Biobank, with 20,626 controls who were tested negative and 4,101 COVID-19 patients, including 970 hospitalized ones. Plasma PUFAs at baseline (blood samples collected from 2007 to 2010) were measured by nuclear magnetic resonance, including total PUFAs, omega-3 PUFAs, omega-6 PUFAs, docosahexaenoic acid (DHA), linoleic acid (LA), and the omega-6/omega-3 ratio. Moreover, going beyond UK Biobank, we leveraged summary statistics from existing genome-wide association studies to perform bidirectional two-sample Mendelian randomization (MR) analyses to examine the causal associations of eight individual PUFAs, measured in either plasma or red blood cells, with COVID-19 susceptibility and severity. In the observational association analysis of each PUFA measure separately, total, omega-3, and omega-6 PUFAs, DHA, and LA were associated with a lower risk of severe COVID-19. Omega-3 PUFAs and DHA were also associated with a lower risk of testing positive for COVID-19. The omega-6/omega-3 ratio was positively associated with risks of both susceptibility and severity. When omega-6, omega-3, and their ratio are jointly analyzed, only omega-3 PUFAs remained significantly and inversely associated with both susceptibility and severity. The forward MR analysis indicated that docosapentaenoic acid (DPA-n3) and arachidonic acid (AA) might be causally associated with a lower risk of severe COVID-19, with OR (95% CI) per one SD increase in the plasma level as 0.89 (0.81, 0.99) and 0.96 (0.94, 0.99), respectively. The reverse MR analysis did not support any causal effect of COVID-19 on PUFAs. Our observational analysis supported that higher circulating omega-3 PUFAs, especially DHA, may lower the susceptibility to and alleviate the severity of COVID-19. Our MR analysis further supported causal associations of DPA-n3 and AA with a lower risk of severe COVID-19.

## Introduction

The coronavirus disease 2019 (COVID-19) pandemic, caused by the severe acute respiratory syndrome coronavirus 2 (SARS-CoV-2), has resulted in over five million deaths in less than 2 years (1, 2). Understanding the role of nutrition in moderating susceptibility to and progression of COVID-19 is critical for the development of evidence-based dietary recommendations to prevent infection and to manage disease progression (3, 4). Omega-3 and omega-6 polyunsaturated fatty acids (PUFAs) are of special interest because of their potent immunomodulatory effects, not only in mounting immune responses against viral infection but also in promoting inflammation resolution to avoid tissue damage (5–7). COVID-19 is an infectious disease characterized by cytokine storm and hyperinflammation in severe cases (8), presenting multiple possible points of action for PUFAs.

Recent observational studies have noted significant changes in the circulating levels of various PUFAs when comparing COVID-19 patients to healthy controls and across severity subgroups of patients. In general, total PUFAs, omega-6 PUFAs, linoleic acid (LA), and the omega-3 index measured as the percentage of eicosapentaenoic acid (EPA) and docosahexaenoic acid (DHA) in red blood cell (RBC) fatty acids, are lower in COVID-19 patients and even lower in severe cases (9–12). A higher omega-3 index in patients was further associated with lower risks of requiring mechanical ventilation and death (9, 10). But conflicting patterns were also reported across cohorts and studies (11, 12), such as elevated levels of LA and arachidonic acid (AA) in COVID-19 patients (12). Moreover, the circulating levels of PUFAs in patients are likely confounded by immune responses to the viral infection and do not represent the effects of pre-infection circulating levels. There is a prospective cohort study that compared hospitalized COVID-19 patients to non-cases and found that almost all PUFA measures, including total PUFAs, omega-6 PUFAs, omega-3 PUFAs, LA, and DHA, are associated with a lower risk of severe COVID-19. The only exception is the omega-6/omega-3 ratio, which exhibits a positive association (13). However, the study did not distinguish the effects on susceptibility and severity, and the usage of non-cases without COVID-19 status as the control did not correct for selection bias in those receiving tests. Altogether, while these observational studies provide valuable insights, they are susceptible to residual confounding and reverse causation. The causal effects of circulating PUFAs on COVID-19 susceptibility and severity remain unclear.

Mendelian randomization (MR) is an analytic tool for inferring the causal effects of an exposure on an outcome of interest (14). MR uses randomly allocated genetic variants related to the exposure as instrumental variables, which are inborn and minimally affected by confounders and reverse causation (15). This method has been widely utilized in recent studies to evaluate the causal roles of specific risk factors in COVID-19, such as body mass index (BMI), white blood cells, some circulating proteins, and smoking (16–19). On the other hand, MR studies have also provided support for the causal clinical effects of circulating PUFAs (Supplementary Table 1). The genetically predicted circulating levels of various PUFAs have been associated with clinical biomarkers, such as blood lipids, white blood cell counts, and blood pressure (20–22). They were also directly associated with risks of specific diseases, such as cardiovascular diseases, diabetes, and cancers (23–27). Therefore, MR is a valuable and cost-effective tool to evaluate the causal roles of circulating PUFAs in COVID-19 susceptibility and severity.

In this study, we first performed an observational analysis in a prospective cohort, UK Biobank, with 4,101 COVID-19 patients, including 970 hospitalized ones, and 20,626 controls that were tested negative. We performed multiple comparisons across different case and control groups to evaluate the effects of six baseline plasma PUFA measures on COVID-19 susceptibility and severity. Furthermore, we applied bidirectional two-sample MR analyses to examine the causal associations between eight individual PUFAs and COVID-19. Genetic instruments for circulating PUFAs were obtained from previous genome-wide association studies (GWAS) of corresponding PUFAs measured in either plasma or RBC (28–30). Genetic associations with COVID-19 susceptibility and severity were obtained from GWAS meta-analyses conducted by the COVID-19 Host Genetics Initiative (HGI) (31). Our study, integrating observational and genetics-instrumented MR analyses, unraveled the effects of total and individual circulating PUFAs on the risks of COVID-19 susceptibility and severity.

## Materials and Methods

### Ethical Considerations

The usage of individual-level data for this study was approved by the University of Georgia Institutional Review Board and UK Biobank (application no. 48818). All participants of UK Biobank and the Framingham Heart Study (FHS) provided written informed consent before joining these studies. Informed consent was not required for publicly available summary statistics. Our study follows the guidelines for strengthening the reporting of observational studies in epidemiology (STROBE, Supplementary Table 2) and strengthening the reporting of Mendelian randomization studies (STROBE-MR, Supplementary Table 3) (32).

### Participants and Study Design

We performed an observational cohort study based on UK Biobank and then a bidirectional two-sample MR study with summary statistics from GWAS of PUFAs and COVID-19. UK Biobank is a population-based prospective study, including >500,000 participants aged 37–73 years at recruitment from 2006 to 2010 in the United Kingdom (33). The observational analysis was performed to examine the associations between six plasma PUFA measures and COVID-19 status in UK Biobank. The six plasma PUFA measures include total PUFAs, omega-3 PUFAs, omega-6 PUFAs, DHA, LA, and the calculated omega-6/omega-3 ratio. The MR study investigated the causal effects of eight individual PUFAs on COVID-19 susceptibility and severity. Genetic instruments for plasma PUFAs were obtained directly from published GWAS (28, 29). Genetic instruments for RBC PUFAs were determined based on a published GWAS, but their summary statistics, not reported in the original study, were calculated by ourselves with the same statistical model and individual-level data from 2,462 FHS participants (30). Six PUFAs have genetic instruments for their circulating levels in both plasma and RBC, including α-linolenic acid (ALA), docosapentaenoic acid (DPA-n3), LA, γ-linolenic acid (GLA), dihomo-γ-linolenic acid (DGLA), and AA. Docosatetraenoic acid (DTA) only has genetic instruments for its RBC level, while DHA only for its plasma level.

### Observational Analysis

**FIGURE 1:**
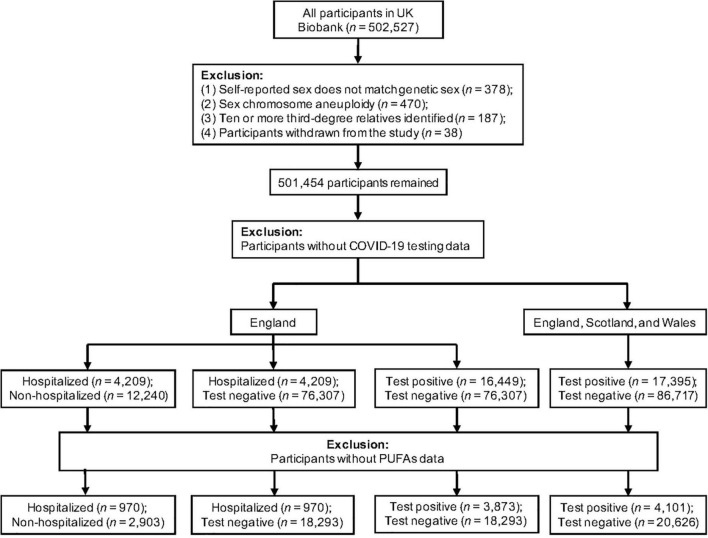
Flowchart of the UK Biobank participants from recruitment to inclusion in the observational analysis.

Figure 1 displays the flow of participants throughout the observational study. To minimize the possibility of bias, we removed participants who had mismatched self-reported sex and genetic sex, sex chromosome aneuploidy, ten or more third-degree or closer relatives, or had withdrawn from UK Biobank. Our exposure variables were six PUFAs, as measured by nuclear magnetic resonance (NMR) in a random subset of plasma samples collected between 2007 and 2010 (13, 33, 34). We used the COVID-19 testing result and inpatient status as our outcome (data accessed on June 21, 2021). The specimen collection dates were March 16, 2020 to June 14, 2021 for those in England; February 11, 2020 to March 18, 2021 in Scotland; and January 13, 2020 to June 7, 2021 in Wales. Hospitalized COVID-19 patients were identified as those with positive PCR-based diagnosis and explicit evidence of being inpatients. Of note, being an inpatient does not necessarily indicate hospitalization for COVID-19 because patients in hospitals for any reason may be prioritized for COVID-19 testing (35). Inpatient status was not available for assessment centers in Scotland and Wales. To test the association with COVID-19 severity, we performed two separate analyses with different controls: (1) non-hospitalized COVID-19 patients, and (2) individuals who tested negative. To examine the association with COVID-19 susceptibility, we focused on all COVID-19 cases, which were tested positive for SARS-CoV-2. Individuals with negative tests were used as the control. This analysis of susceptibility was performed in two datasets: (1) participants from England, and (2) participants from England, Scotland, and Wales. For the 24,727 participants with both plasma PUFA measures and COVID-19 status, we applied logistic regression models on various case and control groups to estimate the associations of PUFAs with COVID-19 susceptibility and severity. Covariates included continuous variables, including age, BMI, and Townsend deprivation index, and categorical variables, including sex, ethnicity, and assessment center. Individuals with missing information in PUFA measures, COVID-19 status, or covariates were excluded. All plasma PUFA measures were standardized to z scores and their comparable effect sizes were expressed per one standard deviation (SD) increase in the corresponding PUFAs. All analyses in the observational study were conducted using R version 4.0.0, and nominal significance was set at *p*-value < 0.05. Bonferroni correction for multiple testing [corrected *P* significance cutoff: 0.05/2 (outcomes)/6 (exposures) = 0.0042] was used to avoid the type I error (36).

### Genetic Associations With Polyunsaturated Fatty Acids

Two types of circulating PUFAs were evaluated in our MR analyses, plasma and RBC PUFAs. For plasma PUFAs, single nucleotide polymorphisms (SNPs) were obtained from published GWAS of omega-3 PUFAs (*n* = 8,866) and omega-6 PUFAs (*n* = 8,631) in participants of European ancestry (28, 29). We selected SNPs for each plasma omega-3 and omega-6 PUFA, which reached genome-wide significance level (*P* < 5 × 10^–8^) and were restricted by linkage disequilibrium (LD) clumping to ensure independence (*r*^2^ < 0.001 within a 10 Mb window). To ensure robustness and reduce false positives, we also used less stringent LD cutoffs (*r*^2^ < 0.01, 0.1, and 0.3) to select SNPs associated with plasma omega-3 PUFAs. The same LD-related sensitivity analysis was not possible for plasma omega-6 PUFAs because their genome-wide summary statistics were not available. To examine the effects of RBC PUFAs, we obtained genetic associations at a genome-wide significance level (*P* < 5 × 10^–8^) identified by Tintle et al. (30). We used the individual-level data from the FHS to confirm the significance of these SNPs and calculate their effect sizes and standard errors. In the same linear mixed model, covariates included age, sex, and matrix of kinship coefficients in the FHS. We respectively selected independent (*r*^2^ < 0.001, 0.01, 0.1, and 0.3 within a 10 Mb window) SNPs predicting RBC PUFAs at genome-wide significance (*P* < 5 × 10^–8^). We calculated *F*-statistics to test instrument strength (*F*-statistics > 10 for all plasma and RBC PUFAs) (37). Summary statistics for the genetic instruments for plasma and RBC PUFAs are openly available for public access (Supplementary Tables 4, 5).

### Genetic Associations With COVID-19

To assess genetic associations with COVID-19 severity, we used three GWAS meta-analyses of only European participants which were conducted by the HGI (release 5, released on January 18, 2021) (31). First, we used the GWAS of severe COVID-19, labeled as study A2, that compared patients confirmed with very severe respiratory symptoms (*n* = 5,101) to the control group of general population samples (*n* = 1,383,241). Second, another HGI GWAS, labeled as study B2, compared hospitalized COVID-19 patients (*n* = 9,986) to general population samples (*n* = 1,877,672). The third severe COVID-19 GWAS utilized in our study, labeled as B1, compared hospitalized COVID-19 patients (*n* = 4,829) to non-hospitalized COVID-19 patients (*n* = 11,816). To assess genetic associations with COVID-19 susceptibility, we used one GWAS by HGI, labeled as study C2, that compared any COVID-19 case (*n* = 38,984) to population controls (*n* = 1,644,784). In addition to these four COVID-19 GWAS used in our primary analysis, we repeated MR analyses using the study A2, B1, B2, and C2 from HGI release 4 (released on October 20, 2020), to examine the consistency of our findings across different data releases. Detailed information about these GWAS is available at the COVID-19 HGI website.^1^

To assess reverse causality, we obtained strong (*P* < 5 × 10^–8^) and independent (*r*^2^ < 0.001 within a 10 Mb clumping window) SNPs associated with COVID-19 phenotypes as genetic instruments. We also used a less stringent selection criterion (*P* < 5 × 10^–6^) to determine the robustness of our results.

### Mendelian Randomization Analyses

Mendelian randomization was used to infer causality between PUFAs and COVID-19 by leveraging genetic data as instrumental variables. We scaled the odds ratio (OR) estimates per SD increment of plasma and RBC PUFAs (% of total fatty acids). We obtained the SNP-specific Wald estimate (ratio of the SNP-outcome effect divided by the SNP-exposure effect) when only one SNP was available. The inverse variance-weighted (IVW) method with a multiplicative random-effects model (≥2 SNPs) was used as the primary analysis (38–40). We used the MR-Egger intercept test to evaluate the extent of unbalanced horizontal pleiotropy, which can lead to a biased causal effect estimate (39). In sensitivity analyses, we applied the MR-Egger and weighted median (WM) methods to account for pleiotropy (39–41). The MR-Egger method provides an unbiased causal estimate even when all SNPs are invalid instruments as long as that the horizontal pleiotropic effects are balanced across SNPs (39). However, MR-Egger can be imprecise and suffer from low statistical power, particularly when based on a small number of SNPs (e.g., <10) (39). The WM method gives robust causal estimates even when up to 50% of SNPs are invalid genetic instruments (41). To test the presence of heterogeneity among genetic instruments, we calculated Cochran’s Q statistic for the IVW method and an extended version of Cochran’s Q statistic (Rücker’s Q’) for the MR-Egger method (42, 43). We utilized Bonferroni correction [corrected *P* significance cutoff: 0.05/2 (outcomes)/7 (exposures) = 0.0036] for multiple testing. Additionally, we required a relationship to be nominally significant (*P* < 0.05) with both measures of the same PUFA (plasma and RBC) and in the case of COVID-19 severity, with different outcome GWAS (study A2, B2, and B1). All MR analyses were performed in R version 4.0.0 with the TwoSampleMR package version 3.6.9 (44).

## Results

### Baseline Characteristics

The flow of UK Biobank participants throughout the observational study is described in Figure 1, while their baseline characteristics are summarized in Table 1. Across all assessment centers in England, Scotland, and Wales, there were 104,112 participants with COVID-19 status. Among them, 17,395 were tested positive for COVID-19. Inpatient status was only reported by assessment centers in England. Of the 92,756 participants with COVID-19 status in England, 16,449 were tested positive, and 4,209 had confirmed inpatient status. Across England, Scotland, and Wales, COVID-19 patients were more likely to be male (*t*-test, *P* = 0.008), with higher BMI (*P* = 9.34 × 10^–14^), but younger than participants with negative testing results (*P* < 2.2 × 10^–16^). Across assessment centers in England, hospitalized COVID-19 patients were older (*P* < 2.2 × 10^–16^), were more likely to be male (*P* = 2.44 × 10^–5^), and had higher BMI (*P* = 1.13 × 10^–14^), when compared to non-hospitalized COVID-19 patients. The three known risk factors of severe COVID-19, age, sex, and BMI, were included as covariates in our observational association analysis.

**TABLE 1:** Characteristics of the UK Biobank participants at baseline.*

	England				England, Scotland, and Wales	
Characteristics	Hospitalized COVID-19	Non-hospitalized COVID-19	Test positive	Test negative	Test positive	Test negative
Participants, *n*	4,209	12,240	16,449	76,307	17,395	86,717
Participants with plasma PUFA measures, *n*	970	2,903	3,873	18,293	4,101	20,626
Age, y	59 [40–70]	51 [40–70]	52 [40–70]	59 [40–70]	52 [40–70]	59 [40–70]
Females, *n* (%)	445 (46)	1,559 (54)	2,004 (52)	9,771 (53)	2,123 (52)	11,145 (54)
Body mass index, kg/m^2^ (SD)	29.55 (5.61)	27.96 (4.94)	28.36 (5.16)	27.69 (4.88)	28.36 (5.14)	27.71 (4.89)
PUFAs, mmol/l (SD)	4.82 (0.81)	4.92 (0.78)	4.89 (0.79)	4.97 (0.80)	4.89 (0.78)	4.96 (0.80)
Omega-3 PUFAs, mmol/l (SD)	0.48 (0.20)	0.49 (0.21)	0.49 (0.20)	0.53 (0.22)	0.49 (0.20)	0.53 (0.22)
DHA, mmol/l (SD)	0.21 (0.074)	0.22 (0.075)	0.22 (0.075)	0.24 (0.084)	0.22 (0.075)	0.23 (0.084)
Omega-6 PUFAs, mmol/l (SD)	4.34 (0.70)	4.42 (0.66)	4.40 (0.67)	4.44 (0.68)	4.40 (0.67)	4.44 (0.68)
LA, mmol/l (SD)	3.29 (0.70)	3.39 (0.65)	3.37 (0.67)	3.39 (0.69)	3.37 (0.66)	3.39 (0.68)

*Values are numbers (%) for categorical variables, mean (SD) or medians [range] for continuous variables. PUFAs, polyunsaturated fatty acids; DHA, docosahexaenoic acid; LA, linoleic acid.

### Observational Association Analysis

Table 2 shows the observational associations between baseline plasma PUFAs and COVID-19 susceptibility and severity. Among participants from England who also had plasma data, there were 18,293 with negative testing results and 3,873 with positive tests. Among the COVID-19 patients, 970 were hospitalized and the other 2,903 were non-hospitalized. Comparing hospitalized patients to those tested negative, we observed a lower risk of COVID-19 severity per SD increase in total PUFAs (OR: 0.88; 95% confidence interval (CI): 0.82, 0.95; *P* = 0.00051), omega-3 PUFAs (OR: 0.82; 95% CI: 0.76, 0.89; *P* = 8.07 × 10^–7^), omega-6 PUFAs (OR: 0.91; 95% CI: 0.85, 0.98; *P* = 0.012), DHA (OR: 0.78; 95% CI: 0.72, 0.85; *P* = 4.56 × 10^–9^), and LA (OR: 0.92; 95% CI: 0.86, 0.99; *P* = 0.023). Using 2,903 non-hospitalized COVID-19 patients as the control group, there were consistently inverse associations of COVID-19 severity with total PUFAs (*P* = 0.0012), omega-3 PUFAs (*P* = 0.0013), omega-6 PUFAs (*P* = 0.0047), DHA (*P* = 8.92 × 10^–5^), and LA (*P* = 0.0079).

**TABLE 2:** Associations of single polyunsaturated fatty acids (PUFAs) with COVID-19 susceptibility and severity.*

	COVID-19 severity						COVID-19 susceptibility					
	Hospitalized vs. non-hospitalized (*n* = 3,873)			Hospitalized vs. test negative (*n* = 19,263)			Test positive vs. test negative (*n* = 22,166)^†^			Test positive vs. test negative (*n* = 24,727)^‡^		
Plasma PUFAs	β	SE	*P*	β	SE	*P*	β	SE	*P*	β	SE	*P*
PUFAs	–0.14	0.043	0.0012	–0.13	0.037	0.00051	–0.029	0.019	0.13	–0.027	0.018	0.13
Omega-3 PUFAs	–0.14	0.044	0.0013	–0.20	0.040	8.07 × 10^–7^	–0.083	0.020	4.29 × 10^–5^	–0.082	0.019	2.27 × 10^–5^
DHA	–0.18	0.045	8.92 × 10^–5^	–0.25	0.042	4.56 × 10^–9^	–0.098	0.021	3.00 × 10^–6^	–0.097	0.020	1.41 × 10^–6^
Omega-6 PUFAs	–0.12	0.043	0.0047	–0.090	0.036	0.012	–0.010	0.019	0.62	–0.0078	0.018	0.67
LA	–0.11	0.043	0.0079	–0.082	0.036	0.023	–0.0066	0.019	0.73	–0.0063	0.018	0.73
Omega-6/omega-3	0.11	0.042	0.0061	0.12	0.029	1.48 × 10^–5^	0.053	0.018	0.0030	0.058	0.017	0.00054

*Only one PUFA measure was included in each logistic regression analysis. Effect sizes (β) per SD increase in the exposure, SEs, and P-values were obtained from the logistic regression analysis of COVID-19 susceptibility and severity. All models were adjusted for age, sex, ethnicity, BMI, Townsend deprivation index, and assessment center. PUFAs, polyunsaturated fatty acids; DHA, docosahexaenoic acid; LA, linoleic acid.

*^†^Data from England only.*

*^‡^Data from England, Scotland, and Wales.*

We further evaluated the effects of baseline plasma PUFAs on COVID-19 susceptibility by comparing COVID-19 patients to those tested negative. Among 24,727 participants in England, Scotland, and Wales, we found a lower risk of getting COVID-19 per SD increase in omega-3 PUFAs (OR: 0.92; 95% CI: 0.89, 0.96; *P* = 2.27 × 10^–5^) and DHA (OR: 0.91; 95% CI: 0.87, 0.94; *P* = 1.41 × 10^–6^). Among 22,166 individuals in England only, we also observed consistently significant associations for omega-3 PUFAs (OR: 0.92; 95% CI: 0.88, 0.96; *P* = 4.29 × 10^–5^) and DHA (OR: 0.91; 95% CI: 0.87, 0.94; *P* = 3.00 × 10^–6^).

The omega-6/omega-3 ratio was significantly associated with an increased risk of severe COVID-19, either by comparing hospitalized patients to participants who tested negative (OR: 1.13; 95% CI: 1.07, 1.20; *P* = 1.48 × 10^–5^) or to non-hospitalized patients (OR: 1.12; 95% CI: 1.03, 1.22; *P* = 0.0061). The ratio was also positively associated with COVID-19 susceptibility when comparing COVID-19 patients to those tested negative in England, Scotland, and Wales (OR: 1.06; 95% CI: 1.03, 1.10; *P* = 0.00054) or in England only (OR: 1.05; 95% CI: 1.02, 1.09; *P* = 0.0030). Notably, these PUFA measures are correlated with each other. For example, in the biggest sample from three regions (*n* = 24,727), there is a medium correlation between omega-6 and omega-3 PUFAs (Spearman’s ρ = 0.46, *P* < 2.2 × 10^–16^). To evaluate if their COVID-19 associations are independent of each other, we jointly evaluate their effects in the same model (Table 3). Only the effects of omega-3 PUFAs persist after controlling for omega-6 PUFAs, the omega-6/omega-3 ratio, or both. In a model including all three PUFA measures, omega-3 PUFAs are associated with a lower risk of hospitalized COVID-19 when compared to those tested negative (OR: 0.86; 95% CI: 0.75, 0.98; *P* = 0.029), and a lower risk of testing positive in the England-only sample (OR: 0.89; 95% CI: 0.82, 0.96; *P* = 9.93 × 10^–4^) and in the sample from three regions (OR: 0.90; 95% CI: 0.84, 0.96; *P* = 2.66 × 10^–3^). Overall, our observational analysis showed that individuals with lower baseline levels of all five examined PUFAs were associated with a higher risk of hospitalized COVID-19, and those with lower levels of omega-3 PUFAs and DHA were also at a higher risk of COVID-19 susceptibility. On the other hand, the omega-6/omega-3 ratio was positively associated with the risks of both COVID-19 susceptibility and severity. A joint analysis further support that these effects were mainly driven by omega-3 PUFAs.

**TABLE 3:** Associations of multiple polyunsaturated fatty acids (PUFAs) with COVID-19 susceptibility and severity.*

Plasma PUFAs	β	SE	*P*	Plasma PUFAs	β	SE	*P*	Plasma PUFAs	β	SE	*P*
**COVID-19 severity**											

**Hospitalized vs. non-hospitalized (*n* = 3,873)**											
Omega3	–0.11	0.049	0.031	Omega6	–0.071	0.048	0.14				
Omega3	–0.12	0.066	0.069	Omega6/Omega3	0.026	0.064	0.69				
Omega6	–0.11	0.043	0.014	Omega6/Omega3	0.099	0.042	0.018				
Omega3	–0.043	0.078	0.58	Omega6	–0.091	0.052	0.080	Omega6/Omega3	0.069	0.068	0.31
**Hospitalized vs. test negative (*n* = 19,263)**											
Omega3	–0.19	0.044	1.61 × 10^–5^	Omega6	–0.015	0.040	0.70				
Omega3	–0.17	0.058	3.08 × 10^–3^	Omega6/Omega3	0.027	0.047	0.56				
Omega6	–0.078	0.036	0.030	Omega6/Omega3	0.12	0.029	3.94 × 10^–5^				
Omega3	–0.15	0.068	0.029	Omega6	–0.028	0.043	0.52	Omega6/Omega3	0.039	0.049	0.43

**COVID-19 susceptibility**											

**Test positive vs. test negative (*n* = 22,166), data from England only**											
Omega3	–0.098	0.023	1.61 × 10^–5^	Omega6	0.031	0.021	0.14				
Omega3	–0.089	0.031	3.97 × 10^–3^	Omega6/Omega3	–0.0072	0.028	0.80				
Omega6	–0.0033	0.019	0.86	Omega6/Omega3	0.052	0.018	3.41 × 10^–3^				
Omega3	–0.12	0.038	9.93 × 10^–4^	Omega6	0.039	0.023	0.088	Omega6/Omega3	–0.027	0.031	0.38
**Test positive vs. test negative (*n* = 24,727), data from England, Scotland, and Wales**											
Omega3	–0.098	0.022	6.42 × 10^–6^	Omega6	0.033	0.020	0.099				
Omega3	–0.075	0.029	9.46 × 10^–3^	Omega6/Omega3	0.0093	0.026	0.72				
Omega6	–0.00060	0.018	0.97	Omega6/Omega3	0.058	0.017	5.99 × 10^–4^				
Omega3	–0.11	0.035	2.66 × 10^–3^	Omega6	0.035	0.022	0.10	Omega6/Omega3	–0.0071	0.029	0.80

*Two or three PUFA measures, shown on the same row, were included in each logistic regression analysis. Effect sizes (β) per SD increase in exposures, SEs, and P-values were reported. All models were adjusted for age, sex, ethnicity, BMI, townsend deprivation index, and assessment center. PUFAs, polyunsaturated fatty acids.

### Bidirectional Mendelian Randomization Analyses

We performed bidirectional MR analyses to examine the causal relationships between individual PUFAs and COVID-19. First, we performed a forward MR analysis to investigate the effects of PUFAs on COVID-19 susceptibility and severity. Second, we conducted a reverse MR analysis to evaluate the causal effects of genetically instrumented COVID-19 on PUFAs. All genetic instruments for PUFAs (*F*-statistics > 31.43) and COVID-19 (*F*-statistics > 30.81) were strong instruments. Six individual PUFAs have existing GWAS for their levels in plasma and RBC, and there are three GWAS on severe COVID-19 (i.e., HGI study A2, B2, B1). Only results that were consistent across these different GWAS were reported here.

**FIGURE 2:**
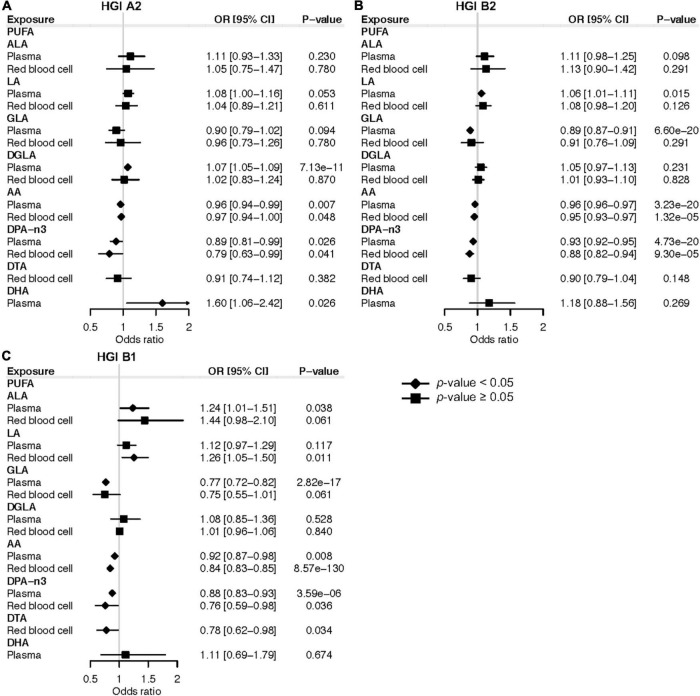
Mendelian randomization estimates of the effects of polyunsaturated fatty acids on COVID-19 severity risk. **(A)** Mendelian randomization analysis based on the release 5 HGI A2. **(B)** Mendelian randomization analysis based on the release 5 HGI B2. **(C)** Mendelian randomization analysis based on the release 5 HGI B1. Odds ratios are scaled to a genetically predicted SD increase in polyunsaturated fatty acids. Associations with *p*-value < 0.05 were indicated with diamonds, while others with squares. Detailed summary statistics are available in Supplementary Tables 6–8. PUFA, polyunsaturated fatty acid; ALA, α-linolenic acid; LA, linoleic acid; GLA, γ-linolenic acid; DGLA, dihomo-γ-linolenic acid; AA, arachidonic acid; DPA-n3, docosapentaenoic acid; DTA, docosatetraenoic acid; DHA, docosahexaenoic acid; OR, odds ratio.

In the forward MR study of plasma PUFAs, genetically instrumented one-SD increase in AA (OR: 0.96; 95% CI: 0.94, 0.99; *P* = 0.007) and DPA-n3 (OR: 0.89; 95% CI: 0.81, 0.99; *P* = 0.026) were associated with a lower risk of very severe respiratory symptoms of COVID-19 based on HGI study A2 (Figure 2A). Consistently, genetically instrumented AA (OR: 0.96; 95% CI: 0.96, 0.97; *P* = 3.23 × 10^–20^) and DPA-n3 (OR: 0.93; 95% CI: 0.92, 0.95; *P* = 4.73 × 10^–20^) were associated with a lower risk of hospitalized COVID-19 based on HGI study B2, which used general population samples as the control (Figure 2B). Similar results were observed with HGI study B1, which used non-hospitalized COVID-19 patients as the control (Figure 2C). Besides plasma PUFAs, MR analyses with RBC PUFAs consistently support the protective effects of AA against severe COVID-19 based on HGI A2 (OR: 0.97; 95% CI: 0.94, 1.00; *P* = 0.048), B2 (OR: 0.95; 95% CI: 0.93, 0.97; *P* = 1.32 × 10^–5^), and B1 (OR: 0.84; 95% CI: 0.83, 0.85; *P* = 8.57 × 10^–130^) studies (Figure 2). For DPA-n3, its genetically instrumented RBC level was consistently associated with a lower risk of COVID-19 severity in our forward MR analysis with study A2 (OR: 0.79; 95% CI: 0.63, 0.99; *P* = 0.041), B2 (OR: 0.88; 95% CI: 0.82, 0.94; *P* = 9.30 × 10^–5^), and B1 (OR: 0.76; 95% CI: 0.59, 0.98; *P* = 0.036) (Figure 2). To ensure the robustness of findings, we selected genetic instruments based on various LD categories (*r*^2^ < 0.001, 0.01, 0.1, and 0.3). The causal estimates of AA and DPA-n3 were consistent and at least nominally significant throughout all MR analyses (Supplementary Tables 6–8). Causal estimates for AA and DPA-n3 maintained the same effect directions in MR-Egger and WM methods, and sensitivity tests identified no evidence of horizontal pleiotropy or heterogeneity of effects (Supplementary Tables 6–8). Of note, while there were nominally significant associations between plasma DHA and very severe COVID-19 with HGI A2 and between RBC DTA and hospitalized COVID-19 with HGI B1, these two relationships were not replicated in analyses with the other two GWAS of severe COVID-19 (Figure 2).

**FIGURE 3:**
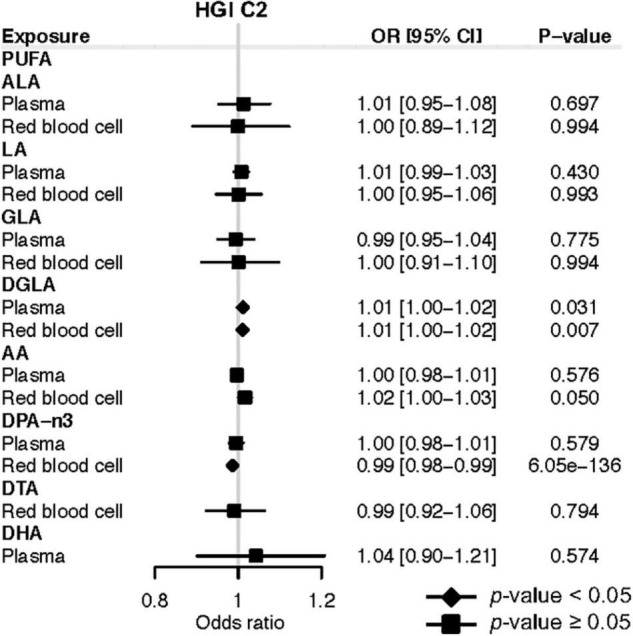
Mendelian randomization estimates of the effects of polyunsaturated fatty acids on COVID-19 susceptibility risk based on the release 5 HGI C2. Odds ratios are scaled to a genetically predicted SD increase in polyunsaturated fatty acids. Associations with *p*-value < 0.05 were indicated with diamonds, while others with squares. Detailed summary statistics are available in Supplementary Table 9. PUFA, polyunsaturated fatty acid; ALA, α-Linolenic acid; LA, linoleic acid; GLA, γ-linoleic acid; DGLA, dihomo-γ-linoleic acid; AA, arachidonic acid; DPA-n3, docosapentaenoic acid; DTA, docosatetraenoic acid; DHA, docosahexaenoic acid; OR, odds ratio.

In terms of COVID-19 susceptibility, we found that genetically instrumented one-SD increase of plasma DGLA (OR: 1.01; 95% CI: 1.00, 1.02; *P* = 0.031) was associated with an increased risk of any SARS-CoV-2 infection (Figure 3). MR analysis with RBC DGLA showed a similar pattern (OR: 1.01; 95% CI: 1.00, 1.02; *P* = 0.007). However, the association of genetically instrumented DGLA with the risk of testing positive for COVID-19 was not statistically significant using any other LD criteria for genetic instruments (Supplementary Table 9). Notably, our forward MR findings were confirmed using additional COVID-19 GWAS from HGI release 4 (Supplementary Tables 10–13). In summary, our forward MR analyses suggest that higher circulating levels of AA and DPA-n3 are associated with a lower risk of developing severe forms of COVID-19.

We further applied reverse MR analyses to investigate the causal effects of COVID-19 on each PUFA. Although several reverse MR analyses showed that genetically instrumented COVID-19 susceptibility or severity was associated with ALA, DHA, GLA, or DGLA, there was no consistent evidence for an effect of COVID-19 on these PUFAs using the conventional genome-wide significance threshold (*P* < 5 × 10^–8^) and the more lenient threshold (*P* < 5 × 10^–6^) for COVID-19 SNPs from HGI release 5 (Supplementary Tables 14–21). In addition, we used SNPs associated with COVID-19 from HGI release 4 and did not observe any causal effect of COVID-19 on PUFAs (Supplementary Tables 22–29). Importantly, the reverse MR results showed no significant association of genetically predicted COVID-19 severity with AA and DPA-n3, suggesting that the significant forward MR results are unlikely to be confounded by reverse causation. Lastly, we performed a supplemental and confirmatory MR analysis utilizing summary statistics of GWAS for four NMR-based plasma PUFA measures, omega-3 PUFAs, omega-6 PUFAs, DHA, and LA (45). The forward MR indicated that higher genetically predicted omega-3 PUFAs were associated with reduced risk of severe COVID-19 based on HGI release 5 study A2 (OR: 0.85; 95% CI: 0.72, 0.99; *P* = 0.034), B1 (OR: 0.76; 95% CI: 0.67, 0.86; *P* = 8.81 × 10^–6^), and B2 (OR: 0.85; 95% CI: 0.76, 0.95; *P* = 0.004) (Supplementary Table 30). No significant association was found for omega-3 PUFAs and COVID-19 susceptibility, nor for any other PUFA measures.

## Discussion

Our observational analysis in a prospective cohort showed that total PUFAs, omega-3 PUFAs, omega-6 PUFAs, DHA, and LA in baseline plasma samples were inversely associated with the risk of severe COVID-19. There were also inverse associations of omega-3 PUFAs and DHA with COVID-19 susceptibility. In contrast, the omega-6/omega-3 ratio was positively associated with both COVID-19 susceptibility and severity. A joint analysis of omega-6 PUFAs, omega-3 PUFAs, and their ratio further revealed that these effects were mainly driven by omega-3 PUFAs. In our bidirectional two-sample MR analyses, we provided evidence for the potential causal roles of higher circulating AA and DPA-n3 in a lower risk of COVID-19 severity.

Our observational findings are broadly consistent with previous observational studies and a pilot clinical trial. Julkunen et al. (13) also examined the UK Biobank cohort, although with smaller sample sizes and different controls. They showed that for total PUFAs, omega-3 PUFAs, omega-6 PUFAs, DHA, and LA, their absolute levels and relative percentages in total fatty acids were both inversely associated with the risk of severe COVID-19 when comparing patients to non-cases with unknown COVID-19 status. Our study corrected for potential selection bias by restricting the analysis to individuals with COVID-19 testing status and used those with negative tests or non-hospitalized patients as the controls. We confirmed the same inverse association patterns for severe COVID-19. We further showed that omega-3 PUFAs and DHA were inversely associated with COVID-19 susceptibility. Importantly, our joint analysis of omega-6 PUFAs, omega-3 PUFAs, and their ratio revealed that these effects were mainly driven by omega-3 PUFAs. Another study investigated the metabolic fingerprint of COVID-19 severity in 581 samples from three cohorts, revealing inverse associations with severity for total PUFAs, omega-6 PUFAs, and LA. But inconsistent associations of omega-3 PUFAs, DHA, and the omega-6/omega-3 ratio were also observed across cohorts (11). Comparing the lipid profile of 42 severe COVID-19 patients to 22 healthy subjects, a study by Perez-Torres et al. (12) found that plasma GLA, DGLA, and EPA were decreased in COVID-19 patients, but LA and AA were elevated. Two small studies found that the omega-3 index was significantly lower in COVID-19 patients and was inversely associated with risks of requiring mechanical ventilation and death (9, 10). The differences in these observational studies are likely results of uncontrolled confounding factors or the usage of patients at different disease stages. In support of the associated protective effect of omega-3 fatty acids, the first randomized clinical trial of supplementing 1,000 mg omega-3 fatty acids in 128 critically ill COVID-19 patients showed that the intervention group had a significantly higher 1-month survival rate and improved respiratory and renal function (46). Altogether with the existing literature, our study supports the protective effects of omega-3 fatty acids against the development of severe COVID-19 and likely also against viral infection.

In our MR study, we examined whether specific individual PUFAs play causal roles in COVID-19 susceptibility and severity. We found that genetically instrumented circulating levels of AA and DPA-n3 are associated with a lower risk of severe COVID-19. AA is an omega-6 fatty acid, while DPA-n3 is an omega-3 fatty acid. Although these two specific PUFAs were not available in our observational analysis, their potentially causal protective effects are consistent with the inverse associations of both omega-6 PUFAs and omega-3 PUFAs with severe COVID-19. The potential protective roles of AA and DPA-n3 in severe COVID-19 have mechanistic support. It is usually generalized that omega-6 PUFAs are precursors to pro-inflammatory signaling molecules, such as the AA-derived prostaglandins (PGs) and leukotrienes, while omega-3 PUFAs, mainly EPA, DPA-n3, and DHA, give rise to anti-inflammatory signaling molecules, such as resolvins, protectins, and maresins. However, the underlying biochemistry and signaling pathways are complex, depending on specific mediating molecules and timing of actions (7, 47). First, both AA and DPA-n3 may modulate the inflammatory process and prevent the development of cytokine storm in COVID-19 patients. Both of them are well-known to serve as precursors of specialized pro-resolving mediators. In addition to resolvins, protectins, and maresins derived from DPA-n3, lipoxins derived from AA play essential roles in promoting the resolution of inflammatory responses and tissue repair (5, 7, 48). Notably, it has been highlighted that the roles of AA in initiating timely inflammatory responses through its derived PGs, such as PGE_2_, may be as important as its roles in inflammatory resolution through lipoxins (6, 47). Second, both AA and DPA-n3 may inhibit virus entry into host cells. LA has been shown to directly and tightly bind the SARS-CoV-2 spike glycoprotein, reducing its interaction with the human ACE2 receptor (49). Similar inhibitory effects were observed for ALA, EPA, and DHA in a ligand screening study (50), which did not include AA and DPA-n3. Third, AA may suppress virus replication in host cells. In a pre-pandemic lipidomics study aiming to comprehensively characterize the host cell lipid response upon coronavirus infection, Huh-7 cells, a hepatocyte-derived carcinoma cell line, when infected with human coronavirus 229E (HCoV-229E), exhibit significantly elevated levels of LA and AA (51), a pattern that is also observed in a recent study of severe COVID-19 patients (12). Interestingly, exogenous supplementation of LA and AA in HCoV-229E-infected cells significantly decreased the virus genome copies in both cell lysates and supernatants, suggesting that LA and AA suppressed HCoV-229E virus replication. Similar suppressive effects were observed for the highly pathogenic Middle East respiratory syndrome coronavirus (MERS-CoV) (51), suggesting a general mechanism of LA and AA on coronavirus. Consistently, it has been known that unsaturated fatty acids, especially AA, can inactivate enveloped viruses, such as influenza and HIV (47). Our MR findings call for future studies into the mechanistic roles of AA and DPA-n3 in the development of severe COVID-19.

Our study has a number of strengths and novel features. Most notably, our study integrates two complementary research approaches, an observational analysis in a prospective cohort and a MR analysis. The observational analyses used, to our knowledge, the largest sample size to date. We also applied multiple comparisons and controls to significantly increase the credibility of the results. The two research approaches revealed consistent patterns. While the observational analyses highlighted omega-3 PUFAs to be negatively associated with both COVID-19 severity and susceptibility, our MR analyses confirmed that total omega-3 PUFAs and DPA-n3 may play causal roles in reducing the risk of severe COVID-19. There are multiple strengths associated with our MR analyses. To our knowledge, this is the first MR study examining the causal effects of PUFAs on COVID-19. It is also the first MR study of PUFAs that used genetic variants for RBC PUFAs, in addition to plasma PUFAs. RBC and plasma PUFAs are two lipid pools that reflect dietary input at varying time frames ranging from months to weeks, with RBC PUFAs reflecting longer-term dietary input and plasma PUFA more impacted by recent dietary intakes. There are medium to high correlations between PUFAs measured in the two sources (52–54). The inclusion of both RBC and plasma PUFAs has at least two benefits. It expanded the list of exposures to include those that only have genetic instruments in one source, including DTA in RBC and DHA in plasma. For PUFAs having genetic instruments in both sources, we only reported consistently significant results to reduce false positives. To obtain robust evidence and to ensure reproducibility across data releases, we confirmed the results with analyses based on four COVID-19 GWAS (A2, B2, B1, and C2) from HGI releases 5 and 4. Bonferroni correction was used to overcome the issue of multiple testing. We also applied sensitivity analysis with various LD cutoffs. Another strength is the application of bidirectional two-sample MR analyses to evaluate the direction of the causality and to rule out the impacts of reverse causation. Additionally, comparing our MR results between severe COVID-19 and any SARS-CoV-2 infection, we found that AA and DPA-n3 might mainly impact the severity of disease progression but not susceptibility to viral infection.

Our study has several limitations. First, we could not completely rule out the possibility that some genetic variants might be pleiotropic, although we applied multiple sensitivity analyses, including the heterogeneity test, MR-Egger, and WM method. We also applied the PhenoScanner to examine the pleiotropic effects of genetic instruments for AA and DPA-n3, which might provide alternative explanations for our MR observations (Supplementary Table 31) (55, 56). However, it is still difficult to distinguish if they represent horizontal or vertical pleiotropic effects. Second, another limitation in the MR analysis is that the population controls have no information on COVID-19 status in three COVID-19 GWAS used in our primary analysis, including the HGI A2, B2, and C2 studies. To mitigate this issue, we also utilized the HGI B1 study, which is another GWAS of COVID-19 using non-hospitalized patients as the control group. Third, dietary intakes of specific PUFAs, which influences their circulating levels, were not available in UK Biobank. So, our observational analysis did not investigate the direct or indirect effects of dietary PUFAs on COVID-19 risk. However, our MR study leveraging genetic instruments yields novel insights into their possible roles. Fourth, UK Biobank recruited healthier individuals and thus may not be representative of the general population. Fifth, the NMR-based measures of plasma PUFAs were collected over 10 years before the COVID-19 pandemic, and the time lag probably attenuates the magnitude of association. Sixth, the NMR-based method only measured two individual PUFAs (DHA and LA), while many other individual PUFAs (e.g., AA, ALA and EPA) were not available for the observational analyses. Notably, our MR study alleviates this limitation by using eight individual PUFAs (i.e., ALA, DPA-n3, DHA, LA, GLA, DGLA, AA, and DTA) from both RBC and plasma. Seventh, our study did not examine saturated or monounsaturated fatty acids. A previous study in UK Biobank found that the percentages of these two groups are both positively associated with the risk of severe COVID-19 (13). Eighth, our observational study could be affected by ascertainment bias in differential healthcare seeking and testing. Being an inpatient does not necessarily indicate hospitalization for COVID-19 because patients in hospitals for any reason may be prioritized for COVID-19 testing. Hospitalized patients and the observed effects of PUFAs might be driven by other diseases instead of COVID-19. One possible mitigation analysis is to use hospitalized non-COVID-19 patients as the control, which was not analyzed in this study. Ninth, our findings might not be extrapolated to other ethnicities because the study mainly focused on participants of European descent. Future studies in large non-European samples are needed to test the generalizability of our observations. Tenth, our study cannot thoroughly explain the mechanisms. Further mechanistic research is necessary to investigate the biological pathways underpinning the roles of PUFAs in severe COVID-19.

## Conclusion

Our observational analysis in a prospective cohort shows that total PUFAs, omega-3 PUFAs, omega-6 PUFAs, DHA, and LA are inversely associated with the risk of severe COVID-19. Omega-3 and DHA may also be protective against SARS-CoV-2. A higher omega-6/omega-3 ratio has adverse effects on both COVID-19 susceptibility and severity. These associations are mainly driven by omega-3 PUFAs. Our MR study further suggests a possible causal role of AA and DPA-n3 in reducing the risk of severe COVID-19. Our findings call for further studies into the mechanistic roles of PUFAs in COVID-19. They also support the possible usage of circulating PUFA levels as biomarkers for identifying high-risk individuals and as therapeutic targets for managing COVID-19 patients.

## Data Availability Statement

The datasets (GWAS summary statistics) of COVID-19 analyzed for this study can be found in the COVID-19 Host Genetics Initiative (https://www.covid19hg.org/). The code for the analyses is available at https://github.com/yitangsun/COVID19_PUFA_MR.

## Author Contributions

YS and KY designed the study, provided statistical advice, and interpreted the data. YS performed data analysis, prepared visualizations, and wrote the manuscript. YS, RC, and AR provided material support during the study. KY critically revised the manuscript. All authors read and approved the final manuscript and took responsibility for the integrity of the work as a whole.

## Conflict of Interest

The authors declare that the research was conducted in the absence of any commercial or financial relationships that could be construed as a potential conflict of interest.

## Publisher’s Note

All claims expressed in this article are solely those of the authors and do not necessarily represent those of their affiliated organizations, or those of the publisher, the editors and the reviewers. Any product that may be evaluated in this article, or claim that may be made by its manufacturer, is not guaranteed or endorsed by the publisher.

## Footnotes

1
https://www.covid19hg.org/results/
