# Corrigendum: Circulating polyunsaturated fatty acids and COVID-19: a prospective cohort study and Mendelian randomization analysis

**DOI:** 10.3389/fmed.2023.1190514

**Published:** 2023-04-12

**Authors:** Yitang Sun, Radhika Chatterjee, Akash Ronanki, Kaixiong Ye

**Affiliations:** ^1^Department of Genetics, Franklin College of Arts and Sciences, University of Georgia, Athens, GA, United States; ^2^Institute of Bioinformatics, University of Georgia, Athens, GA, United States

**Keywords:** COVID-19, polyunsaturated fatty acid, Mendelian randomization, prospective cohort, docosapentaenoic acid, arachidonic acid

In the published article, there was an error. Acknowledgments of the Framingham Heart Study were not included. A correction has been made to Acknowledgments. This sentence previously stated:

“This study was approved by the University of Georgia Institutional Review Board and the UK Biobank consortium (application no. 48818). We thank the investigators of the COVID-19 Host Genetics Initiative for sharing the GWAS summary statistics.”

The corrected sentence appears below:

“This study was approved by the University of Georgia Institutional Review Board and the UK Biobank consortium (application no. 48818). We thank the investigators of the COVID-19 Host Genetics Initiative for sharing the GWAS summary statistics. The Framingham Heart Study is conducted and supported by the National Heart, Lung, and Blood Institute (NHLBI) in collaboration with Boston University (Contract Nos. N01-HC-25195, HHSN268201500001I and 75N92019D00031). This manuscript was not prepared in collaboration with investigators of the Framingham Heart Study and does not necessarily reflect the opinions or views of the Framingham Heart Study, Boston University, or NHLBI. Funding for SHARe Affymetrix genotyping was provided by NHLBI Contract N02-HL-64278. SHARe Illumina genotyping was provided under an agreement between Illumina and Boston University. Funding for Affymetrix genotyping of the FHS Omni cohorts was provided by Intramural NHLBI funds from Andrew D. Johnson and Christopher J. O'Donnell. Funding support for the Framingham Red Blood Cell Fatty Acids dataset was provided by NHLBI grant R01 HL089590.”

The authors apologize for this error and state that this does not change the scientific conclusions of the article in any way. The original article has been updated.

