# Supplementary material for: Circulating Polyunsaturated Fatty Acids and COVID-19: A Prospective Cohort Study and Mendelian Randomization Analysis
Source: Front Med (Lausanne). 2022 Jun 16;9:923746. doi: 10.3389/fmed.2022.923746 (PMC9243664; doi:10.3389/fmed.2022.923746)
Supplement: Supplementary file 1 [file Table_1.docx]

Supplementary Material

**Supplementary Table 1.** Notable Mendelian randomization studies of polyunsaturated fatty acids.

| **MR study** | **Exposure** | **Exposure data sources** | **Exposure unit** | **Outcome** | **Outcome data sources** | **Findings** **(**increasing risk, decreasing risk, null**)** |
| --- | --- | --- | --- | --- | --- | --- |
| Zhao, J.V., Schooling, C.M (1) | LA | CHARGE consortium | % total increase in total fatty acids | IHD, diabetes, HDL-C, LDL-C, TC, BP, and reticulocyte count | CARDIoGRAMplusC4D 1000 Genomes, the Myocardial Infarction Genetics and CARDIoGRAM Exome, the UK Biobank SOFT CAD GWAS, the DIAbetes Genetics Replication And Meta-analysis diabetes case study, Global Lipids Genetics Consortium, and the UK Biobank | Genetically predicted higher serum LA was associated with the lower diabetes risk, lower levels of HDL-C, LDL-C, and TC. Genetically predicted LA was not associated with IHD, SBP, or reticulocyte count. |
| Zhang, T. *et al.* (2) | AA | CHARGE consortium | SD | IHD, stroke, HDL-C, LDL-C, TG, ApoB, ApoA-I, BP, adiposity, and markers of inflammation and coagulation | The CARDIoGRAMplusC4D 1000 Genomes, MEGASTROKE consortium, the UK Biobank pan-ancestry summary statistics, GLGC, the Genetic Investigation of ANthropometric Traits Consortium and the UK Biobank meta-analysis, a GWAS of cytokines in 8293 Finnish individuals, the INTERVAL study in 3301 individuals of European ancestry, the CHARGE Inflammation Working Group, and the Biobank Japan. | Genetically predicted AA was associated with higher levels of ApoB, HDL-C, LDL-C, and a lower level of TG, but not associated with IHD, stroke, ApoA-I, BP, adiposity, or other markers of inflammation and coagulation. |
| Yuan, S. *et al.* (3) | ALA, EPA, DPA, DHA, LA, AA, POA, OA, PA, and SA | CHARGE consortium | SD | CAD, IS, AF, heart failure, aortic valve stenosis, abdominal and thoracic aortic aneurysms, transient ischemic attack, intracerebral and subarachnoid hemorrhages, VTE, and PAD | CARDIoGRAMplusC4D consortium, MEGASTROKE consortium, Atrial Fibrillation consortium, and UK Biobank | Genetically predicted plasma AA and SA levels were associated with the higher CVD risk. Genetically predicted higher plasma ALA, LA, and OA levels were associated with lower risks of large-vessel stroke and venous thromboembolism. |
| Yuan, T. *et al.* (4) | LA, AA, ALA, EPA, DHA, and DPA | CHARGE consortium | SD | IS, LDL-C, HDL-C, TC, SBP, and DBP | The International Stroke Genetics Consortium, the GLGC, and the UK Biobank | Genetically predicted AA was associated with higher IS risk, higher levels of LDL-C, HDL-C, and TC. Genetically predicted serum ALA was associated with a lower IS risk, lower levels of LDL-C, HDL-C, and TC. Genetically predicted LA, EPA, DHA, and DPA were not associated with IS, lipids, or BP. |
| Liao, L. Z. *et al.* (5) | Omega-6 fatty acids | Large GWAS (n = 24,925) from 14 cohorts in people of European ancestry | SD | CHD, TC, LDL-C, HDL-C, and TG | CARDIoGRAMplusC4D and GLGC | Genetically predicted omega-6 was associated with a higher risk of CHD, which might be partially mediated by TC, LDL-C, and TG. |
| Mazidi, M. *et al.* (6) | 10-heptadecenoate, MA, OA, and POA | A GWAS (including 7,824 adult samples of European ancestry) | SD | CHD, MI, CS, and IS | CARDIoGRAMplusC4D 1000 Genomes, UK Biobank SOFT CAD study, two small case (n = 4,120)–control (n = 3,910) studies from Germany and Greece, and METASTROKE | Genetically predicted serum 10-heptadecenoate, MA, OA, and POA were not associated with risks of CHD, MI, CS, or IS. |
| Zhang, T. *et al.* (7) | AA | CHARGE consortium | % total increase in fatty acid | ASCVD, IHD, PAD, VTE, and other CVD | CARDIoGRAMplusC4D 1000 Genomes, MEGASTROKE, Pan-UK Biobank, Atrial Fibrillation Consortium, HERMES consortium, and FinnGen. | Genetically predicted AA was associated with higher risks of ASCVD, IHD, PAD, and VTE, with possibly stronger associations in men than women. |
| Yuan, S., & Larsson, S. C. (8) | ALA, DHA, EPA, DPA, AA, and LA | CHARGE consortium | SD | AF | Atrial Fibrillation Consortium | Genetically predicted ALA, DHA, EPA, DPA, AA, and LA were not associated with the risk of AF. |
| Park, S. *et al.* (9) | DPA, EPA, DHA, LA, GLA, DGLA, AdrA, and AA | CHARGE consortium | SD | CAD and MI | UK Biobank and CARDIoGRAMplusC4D | Genetically predicted AA was associated with a higher risk of CAD. Genetically predicted higher EPA, LA, and DGLA levels were associated with a lower risk of CAD. |
| Wang, W *et al.* (10) | MUFA and omega-3 fatty acids | Large GWAS (n = 24,925) from 14 cohorts in people of European ancestry | SD | Incident heart failure and mortality of heart failure | Cohorts for Heart and Aging Research in Genomic Epidemiology-Heart Failure Working Group | Genetically predicted higher circulating MUFA levels were associated with increased risk of heart failure, but no association with mortality of heart failure. Genetically predicted omega-3 fatty acids were not associated with heart failure. |
| Chen, H. Y. *et al.* (11) | AA | FOS cohort (n = 1310, 492 cases of AVC) and European-ancestry participants in the MESA cohort (n = 2415) | SD | AVC and AS | Genetic Epidemiology Research on Adult Health and Aging cohort and CHARGE Consortium | Genetically predicted higher AA level was associated with higher risks of AS and AVC. |
| Yuan, S. *et al.* (12) | ALA, EPA, DHA, LA, AA, POA, OA, PA, and SA | CHARGE consortium | SD | T2DM | DIAGRAM consortium | Genetically predicted higher EPA, DPA, AA, and SA were associated with a higher risk of T2DM. Genetically predicted higher ALA, LA, POA, and OA levels were associated with a lower risk of T2DM. |
| Jäger, S. *et al.* (13) | D5D (AA/DGLA) and D6D (GLA/LA) | EPIC–Potsdam Study | SD | T2DM and CAD | DIAGRAM consortium and CARDIoGRAM. | Genetically predicted higher D6D and D5D were associated with higher risks of T2DM and CAD. |
| Adams, C. D., & Neuhausen, S. L. (14) | TOTFA, MUFA, omega-3 fatty acids, and PUFA | Large GWAS (n = 24,925) from 14 cohorts in people of European ancestry | SD | Chronotype and T2DM | UK Biobank and DIAGRAM consortium | Genetically predicted higher TOTFA and MUFA levels were associated with a lower risk of T2DM. |
| Zulyniak *et al.* (15) | AA | CHARGE consortium | SD | DI and T2DM | The Meta-Analysis of Glucose- and Insulin-Related Traits Consortium and the UK Biobank | Genetically predicted higher AA was associated with DI. Genetically predicted AA was not associated with T2DM. |
| Ma, M. *et al.* (16) | ALA, EPA, DPA, DHA, LA, and AA | CHARGE consortium | SD | BP, SBP, DBP, and PP | UK Biobank and International Consortium for Blood Pressure | Genetically predicted higher ALA was associated with higher DBP. Genetically predicted higher AA and EPA were associated with lower DBP. Genetically predicted ALA, EPA, DPA, DHA, LA, and AA were not associated with SBP. Genetically predicted DPA and DHA were not associated with DBP. |
| Zhao, J. V., & Schooling, C. M.(17) | LA | Large GWAS (n = 24,925) from 14 cohorts in people of European ancestry | SD | Asthma, eosinophil, neutrophil, and monocyte counts | Case-control study of asthma based on Trans-National Asthma Genetic Consortium and UK Biobank | Genetically predicted higher LA was associated with a lower risk of asthma, lower eosinophil count, and lower neutrophil count. |
| Liyanage, U. *et al.* (18) | EPA, ALA, LA, AA, DHA, and DPA | CHARGE consortium | SD | Overall cancer risk and cancer mortality | UK Biobank | Genetically predicted higher AA was associated with an increased risk for colorectal cancer. Genetically predicted EPA, ALA, LA, AA, DHA, and DPA were not associated with overall cancer risk or mortality. |
| Larsson, S. *et al.* (19) | AA, EPA, and DHA | CHARGE consortium | SD | Ten site-specific cancers (esophagus, stomach, colorectum, pancreas, lung, bladder, prostate, breast, uterus, and ovaries) | BioBank Japan, Breast Cancer Association Consortium, ILCCO, Ovarian Cancer Association Consortium, Prostate Cancer Association Group to Investigate Cancer Associated Alterations in the Genome consortium | Genetically predicted higher plasma phospholipid AA concentrations were associated with increased risks of colorectal cancer, lung cancer, and esophageal cancer. Genetically predicted AA, EPA, and DHA were not associated with cancers of the stomach, pancreas, bladder, prostate, breast, uterus, or ovary. |
| Liu, J. *et al.* (20) | DPA | CHARGE consortium | % total increase in fatty acid | Lung cancer risk | ILCCO | Genetically predicted higher DPA was associated with an increased risk for lung cancer. |
| Shen, J. *et al.* (21) | Other PUFA than 18:2 in blood, DHA, DPA, EPA, AA, DGLA, and LA | Large GWAS (n = 24,925) from 14 cohorts in people of European ancestry and a GWAS (including 7,824 adult samples of European ancestry) | SD | Lung cancer, LUAD, and LUSC | ILCCO | Genetically predicted higher other PUFA than 18:2, DPA, EPA, and AA were associated with an increased risk for lung cancer. |
| Yang, Z. *et al.* (22) | ALA, EPA, DPA, DHA, LA, AA, POA, OA, PA, and SA | CHARGE consortium | SD | Breast cancer and prostate cancer | Breast Cancer Association Consortium, Prostate Cancer Association Group to Investigate Cancer-Associated Alterations in the Genome consortium | Genetically predicted ALA, EPA, DPA, DHA, LA, AA, POA, OA, PA, and SA were not associated with breast cancer or prostate cancer. |
| Guo, F. *et al.* (23) | SFA, MUFA, and PUFA | Large GWAS (n = 24,925) from 14 cohorts in people of European ancestry | SD | Breast cancer | The National Health and Nutrition Examination Survey | Genetically predicted MUFA was associated with a reduced risk of breast cancer. |
| Khankari, N., Murff, H., Zeng, C. *et al.* (24) | LA, AA, ALA, EPA, DPA, and DHA | CHARGE consortium | SD | Prostate cancer | Prostate Cancer Association Group to Investigate Cancer Associated Alterations in the Genome | Genetically predicted LA, AA, ALA, EPA, DPA, and DHA were not associated with prostate cancer risk. |
| Ghoneim, D. H. *et al.* (25) | LA, AA, AdrA, GLA, and DGLA | CHARGE consortium | SD | Pancreatic cancer | Pancreatic Cancer Cohort Consortium and the Pancreatic Cancer Case-Control Consortium | Genetically predicted LA, AA, AdrA, GLA, and DGLA were not associated with pancreatic cancer risk. |
| Seviiri, M. *et al.* (26) | ALA, EPA, DPA, DHA, AA, and LA | CHARGE consortium | SD | Keratinocyte cancer, BCC, and SCC | UK Biobank, 23andMe, and QSkin Sun and Health Study cohort | Genetically predicted higher AA and EPA were associated with a higher BCC risk. Genetically predicted higher LA and ALA were associated with a reduced BCC risk. |
| Khankari, N. K. et al. (27) | LA, AA, ALA, EPA, DPA, and DHA | CHARGE Consortium | % total increase in fatty acid | CRC | Colorectal Cancer Consortium | Genetically predicted higher AA, EPA, and DPA were associated with an increased CRC risk. Genetically predicted higher LA and ALA were associated with a reduced CRC risk. |
| May-Wilson, S. *et al.* (28) | EPA, DPA, DHA, LA, AA, DGLA, OA, POA, PA, and SA | CHARGE consortium | % total increase in fatty acid | CRC | Colon Cancer Family Registry, COIN trial, Finnish Colorectal Cancer Predisposition Study, colorectal Tumour Gene Identification Consortium, Colorectal Cancer Susceptibility Study, and VQ58 | Genetically predicted higher AA and SA were associated with an increased CRC risk. Genetically predicted higher LA, OA, and POA were associated with reduced CRC risk. |
| Isom, C.A. *et al.* (29) | AA | Tennessee Colorectal Polyps Study | % total increase in fatty acid | Colorectal adenoma | Tennessee Colorectal Polyps Study | Genetically predicted AA was not associated with colorectal adenoma risk. |
| Liyanage, U. *et al.* (30) | EPA, DPA, DHA, ALA, AA, and LA | CHARGE consortium | SD | Melanoma | Melanoma risk meta-analysis consisting of 11 GWAS | Genetically predicted plasma EPA, DPA, DHA, ALA, AA, and LA were not associated with melanoma risk. |
| Saunders, C. N. *et al.* (31) | MUFA, omega-3, and omega-6 fatty acids | Large GWAS (n = 24,925) from 14 cohorts in people of European ancestry | SD | Glioma | A GWAS of glioma | Genetically predicted higher MUFA and omega-3 fatty acids were associated with a reduced glioma sk. |
| Yuan, S. *et al.* (32) | ALA, EPA, DPA, DHA, LA, AA, POA, OA, PA, and SA | CHARGE Consortium | SD | Fracture and eBMD | UK Biobank | Genetically predicted higher plasma ALA, LA, PA, and OA levels were associated with an increased eBMD risk. Genetic predisposition to higher plasma AA, EPA, DPA, and SA levels were associated with an increased risk of fracture. Genetically predicted higher ALA, LA, PA, and OA levels were associated with a reduced risk of fracture. Genetically predicted higher plasma AA, EPA, DPA, and SA levels were associated with a lower risk of eBMD. Genetically predicted plasma DHA and PA were not associated with the risk of eBMD or fracture. |
| Zhao, J. V., & Schooling, C. M. (33) | LA | CHARGE consortium | NA | RA and SLE | UK Biobank, Rheumatoid Arthritis Consortium, and ImmunoBase Consortium | Genetically predicted higher LA was associated with reduced risks of RA and SLE. |
| Sun, L. *et al.* (34) | POA and OA | CHARGE consortium | SD | RA and osteoarthritis | A trans-ethnic GWASs and UK Biobank | Genetically predicted higher plasma levels of POA and OA were associated with a lower RA risk. Genetically predicted plasma POA and OA were not associated with osteoarthritis risk. |
| Wang *et al.* (35) | LA and AA | CHARGE consortium | % total increase in total fatty acids | AMD | International AMD Genomics  Consortium | Genetically predicted higher AA was associated with an increased AMD risk. Genetically predicted higher plasma LA was associated with a lower risk of AMD. |
| Carreras-Torres, R. *et al.* (36) | PUFA, omega-3 and omega-6 fatty acids, MUFA, and TFA | Large GWAS (n = 24,925) from 14 cohorts in people of European ancestry | SD | IBD, CD, and UC | IBD Genetics Consortium and the UK IBD Genetics Consortium | Genetically predicted higher omega-3 fatty acids levels were associated with a lower risk of CD. |
| Cheng, T. S. *et al.* (37) | DGLA and PA | EPIC–Potsdam Study | SD | Puberty timing | UK Biobank | Genetically predicted higher DGLA was associated with earlier menarche in girls. |
| Milaneschi, Y. *et al.* (38) | Omega-3 fatty acids | Large GWAS (n = 24,925) from 14 cohorts in people of European ancestry | SD | MDD | The Psychiatric Genomics Consortium | Genetically predicted omega-3 fatty acids were not associated with MDD risk. |
| Tomata *et al.* (39) | LA, AA, ALA, EPA, DPA, and DHA | CHARGE consortium | SD | Late-onset AD | Alzheimer Disease Genetics Consortium, CHARGE, The European Alzheimer’s Disease Initiative, and Genetic and Environmental Risk in AD/Defining Genetic, Polygenic and Environmental Risk for Alzheimer’s Disease Consortium | Genetically predicted LA, AA, ALA, EPA, DPA, and DHA were not associated with late-onset AD risk. |
| Wang, Z. *et al.* (40) | AA, cis-trans-18:2, DGLA, DPA, EPA, GLA, LA, PA, and SA | CHARGE consortium | SD | AD | International Genomics of Alzheimer’s Project | Genetically predicted higher plasma LA was slightly associated with a lower risk of AD. |
| Jones, H. J. *et al.* (41) | ALA, EPA, DPA, DHA, LA, AA, and AdrA | CHARGE consortium | SD | Schizophrenia | Schizophrenia Working Group of the Psychiatric Genomics Consortium | Genetically predicted higher ALA was associated with the increased schizophrenia risk. Genetically predicted higher plasma DHA was associated with a lower risk of schizophrenia. |
| Sallis, H. *et al.* (42) | DHA and EPA | ALSPAC cohort | % total increase in total fatty acids | Perinatal onset depression, antenatal depression, and postnatal depression. | ALSPAC cohort | Genetically predicted EPA and DHA were not associated with depression risk. |
| Thompson, A.D. *et al.* (43) | DHA, LA, omega-3, and omega-6 fatty acids | Large GWAS (n = 24,925) from 14 cohorts in people of European ancestry | SD | PE's and psychotic disorder | The Psychosis-LIKe Symptoms interview | Genetically predicted DHA, LA, omega-3, and omega-6 fatty acids were not associated with PE's or psychotic disorder. |

LA, linoleic acid; AA, arachidonic acid; ALA, α-linolenic acid; EPA, eicosapentaenoic acid; DPA, docosapentaenoic acid; DHA, docosahexaenoic acid; POA, palmitoleic acid; MA, myristoleic acid; OA, oleic acid; PA, palmitic acid; SA, stearic acid; GLA, γ-linolenic acid; DGLA, dihomo-γ-linolenic acid; AdrA, adrenic acid; MUFA, monounsaturated fatty acid; D5D, Δ5-desaturase; D6D, Δ6-desaturase; TOTFA, total fatty acid; PUFA, polyunsaturated fatty acid; SFA, saturated fatty acid; CHARGE, Cohorts for Heart and Aging Research in Genomic Epidemiology; FOS, Framingham Offspring Study; MESA, Multi-Ethnic Study of Atherosclerosis; EPIC, European Prospective Investigation into Cancer and Nutrition; ALSPAC, the Avon Longitudinal Study of Parents and Children; SD, standard deviation; IHD, ischemic heart disease; HDL-C, high-density lipoprotein cholesterol; LDL-C, low-density lipoprotein cholesterol; TC, total cholesterol; TG, triglycerides; BP, blood pressure; SBP, systolic blood pressure; DBP, diastolic blood pressure; ApoA-I, apolipoprotein A-I; ApoB, apolipoprotein B; CAD, coronary artery disease; AF, atrial fibrillation; IS, ischemic stroke; MI, myocardial infarction; CS, cardioembolic stroke; IS, ischemic stroke; ASCVD, atherosclerotic cardiovascular diseases; PAD, peripheral artery disease; CVD, cardiovascular diseases; VTE, venous thromboembolism; MI, myocardial infarction; AVC, aortic valve calcium; AS, aortic stenosis; T2DM, Type 2 diabetes; DI, insulin disposition index; PP, pulse pressure; LUAD, lung adenocarcinoma; LUSC, lung squamous cell carcinoma; BCC, basal cell carcinoma; SCC, squamous cell carcinoma; CRC, colorectal cancer; eBMD, estimated bone mineral density; RA, rheumatoid arthritis; SLE, systemic lupus erythematosus; AMD, age-related macular degeneration; IBD, inflammatory bowel diseases; CD, Crohn’s disease; UC, ulcerative colitis; MDD, major depressive disorder; AD, Alzheimer’s disease; PE's, later psychotic experiences; GLGC, Global Lipids Genetics Consortium; DIAGRAM, DIAbetes Genetics Replication And Meta-analysis; CARDIoGRAM, Coronary ARtery DIsease Genome wide Replication and Meta-analysis; ILCCO, International Lung Cancer Consortium.

**Supplementary Table 2.** STROBE Statement—checklist of items that should be included in reports of cohort studies.

|  | Item No. | Recommendation | Reported on section and paragraph |
| --- | --- | --- | --- |
| **Title and abstract** | 1 | (a) Indicate the study’s design with a commonly used term in the title or the abstract | Title: “Circulating Polyunsaturated Fatty Acids and COVID-19: A Prospective Cohort Study and Mendelian Randomization Analysis”; abstract |
|  |  | (b) Provide in the abstract an informative and balanced summary of what was done and what was found | Abstract |
| Introduction | | | |
| Background/rationale | 2 | Explain the scientific background and rationale for the investigation being reported | Introduction, paragraphs 1-2 |
| Objectives | 3 | State specific objectives, including any prespecified hypotheses | Introduction, paragraph 4 |
| Methods | | | |
| Study design | 4 | Present key elements of study design early in the paper | Methods, section of “Participants and Study Design” |
| Setting | 5 | Describe the setting, locations, and relevant dates, including periods of recruitment, exposure, follow-up, and data collection | Methods, section of “Participants and Study Design”; section of “Observational Analysis” |
| Participants | 6 | (a) Give the eligibility criteria, and the sources and methods of selection of participants. Describe methods of follow-up | Methods, section of “Participants and Study Design”; section of “Observational Analysis”; Figure 1 |
|  |  | (b) For matched studies, give matching criteria and number of exposed and unexposed | NA |
| Variables | 7 | Clearly define all outcomes, exposures, predictors, potential confounders, and effect modifiers. Give diagnostic criteria, if applicable | Methods, section of “Participants and Study Design”; section of “Observational Analysis” |
| Data sources/ measurement | 8* | For each variable of interest, give sources of data and details of methods of assessment (measurement). Describe comparability of assessment methods if there is more than one group | Methods, section of “Observational Analysis”; Figure 1 |
| Bias | 9 | Describe any efforts to address potential sources of bias | Methods, section of “Observational Analysis” |
| Study size | 10 | Explain how the study size was arrived at | Methods, section of “Observational Analysis”; Figure 1 |
| Quantitative variables | 11 | Explain how quantitative variables were handled in the analyses. If applicable, describe which groupings were chosen and why | Methods, section of “Observational Analysis” |
| Statistical methods | 12 | (a) Describe all statistical methods, including those used to control for confounding | Methods, section of “Observational Analysis” |
|  |  | (b) Describe any methods used to examine subgroups and interactions | Methods, section of “Observational Analysis”; Figure 1 |
|  |  | (c) Explain how missing data were addressed | Methods, section of “Observational Analysis”; Figure 1 |
|  |  | (d) If applicable, explain how loss to follow-up was addressed | Methods, section of “Observational Analysis”; Figure 1 |
|  |  | (e) Describe any sensitivity analyses | Methods, section of “Observational Analysis” |
| Results | | | |
| Participants | 13* | (a) Report numbers of individuals at each stage of study—eg numbers potentially eligible, examined for eligibility, confirmed eligible, included in the study, completing follow-up, and analysed | Results, section of “Baseline Characteristics”; Figure 1; Table 1 |
|  |  | (b) Give reasons for non-participation at each stage | Methods, section of “Observational Analysis” |
|  |  | (c) Consider use of a flow diagram | Results, section of “Baseline Characteristics”; Figure 1 |
| Descriptive data | 14* | (a) Give characteristics of study participants (eg demographic, clinical, social) and information on exposures and potential confounders | Results, section of “Baseline Characteristics” and “Observational Association Analysis”; Table 1 |
|  |  | (b) Indicate number of participants with missing data for each variable of interest | Methods, section of “Observational Analysis”; and Results, section of “Baseline characteristics” and “Observational association analysis”; Figure 1; Table 1 |
|  |  | (c) Summarise follow-up time (eg, average and total amount) | NA |
| Outcome data | 15* | Report numbers of outcome events or summary measures over time | Results, section of “Baseline characteristics”; section of “Observational association analysis”; Figure 1; Tables 1-3 |
| Main results | 16 | (a) Give unadjusted estimates and, if applicable, confounder-adjusted estimates and their precision (eg, 95% confidence interval). Make clear which confounders were adjusted for and why they were included | Results, section of “Observational association analysis”; Tables 2-3 |
|  |  | (b) Report category boundaries when continuous variables were categorized | NA |
|  |  | (c) If relevant, consider translating estimates of relative risk into absolute risk for a meaningful time period | NA |
| Other analyses | 17 | Report other analyses done—eg analyses of subgroups and interactions, and sensitivity analyses | Results, section of “Observational Association Analysis”; Tables 2-3 |
| Discussion | | | |
| Key results | 18 | Summarise key results with reference to study objectives | Discussion, paragraph 1 |
| Limitations | 19 | Discuss limitations of the study, taking into account sources of potential bias or imprecision. Discuss both direction and magnitude of any potential bias | Discussion, paragraph 5 |
| Interpretation | 20 | Give a cautious overall interpretation of results considering objectives, limitations, multiplicity of analyses, results from similar studies, and other relevant evidence | Discussion, paragraph 2 |
| Generalisability | 21 | Discuss the generalisability (external validity) of the study results | Discussion, paragraph 5 |
| Other information | | | |
| Funding | 22 | Give the source of funding and the role of the funders for the present study and, if applicable, for the original study on which the present article is based | Funding |

*Give information separately for exposed and unexposed groups.

**Note:** An Explanation and Elaboration article discusses each checklist item and gives methodological background and published examples of transparent reporting. The STROBE checklist is best used in conjunction with this article (freely available on the Web sites of PLoS Medicine at <http://www.plosmedicine.org/>, Annals of Internal Medicine at <http://www.annals.org/>, and Epidemiology at <http://www.epidem.com/>). Information on the STROBE Initiative is available at <http://www.strobe-statement.org>.

**Supplementary Table 3.** STROBE-MR checklist.

| **Item** | **Complete/location** |
| --- | --- |
| 1. **Title and Abstract:** "Mendelian randomization" is named both in the title and the abstract | Title and abstract |
| **Introduction** |  |
| 1. **Background:** Explain the scientific background and rationale for the reported study. Is causality between exposure and outcome plausible? Justify why MR is a helpful method to address the study question. | Introduction, paragraphs 1-3 |
| 1. **Objectives:** State specific objectives clearly, including pre-specified causal hypotheses (if any). | Introduction, paragraph 4 |
| **Methods** |  |
| 1. **Study design and data sources:** Present key elements of study design early in the paper. Consider including a table listing sources of data for all phases of the study. For each data source contributing to the analysis, describe the following:   a) Describe the study design and the underlying population from which it was drawn. Describe also the setting, locations, and relevant dates, including periods of recruitment, exposure, follow-up, and data collection, if available.  b) Give the eligibility criteria, and the sources and methods of selection of participants.  c) Explain how the analyzed sample size was arrived at.  d) Describe measurement, quality and selection of genetic variants.  e) For each exposure, outcome and other relevant variables, describe methods of assessment and, in the case of diseases, the diagnostic criteria used.  f) Provide details of ethics committee approval and participant informed consent, if relevant. | a) Methods, section of “Participants and Study Design”  b) Methods, section of “Participants and Study Design”  c) Methods, section of “Participants and Study Design”; section of “Genetic Associations with Polyunsaturated Fatty Acids”; section of “Genetic Associations with COVID-19”  d) Methods, section of “Genetic Associations with Polyunsaturated Fatty Acids”; section of “Genetic Associations with COVID-19”  e) Methods, section of “Genetic Associations with Polyunsaturated Fatty Acids”; section of “Genetic Associations with COVID-19”  f) Methods, section of “Ethical Considerations” |
| 1. **Assumptions:** Explicitly state assumptions for the main analysis (e.g. relevance, exclusion, independence, homogeneity) as well assumptions for any additional or sensitivity analysis. | Methods, section of “Genetic Associations with Polyunsaturated Fatty Acids”; section of “Genetic Associations with COVID-19”; section of “Mendelian Randomization Analyses” |
| 1. **Statistical methods main analysis**   Describe statistical methods and statistics used.  a) Describe how quantitative variables were handled in the analyses (i.e., scale, units, model).  b) Describe the process for identifying genetic variants and weights to be included in the analyses (i.e, independence and model). Consider a flow diagram.  c) Describe the MR estimator, e.g. two-stage least squares, Wald ratio, and related statistics. Detail the included covariates and, in case of two-sample MR, whether the same covariate set was used for adjustment in the two samples.  d) Explain how missing data were addressed.  e) If applicable, say how multiple testing was dealt with. | a) Methods, section of “Genetic Associations with Polyunsaturated Fatty Acids”; section of “Genetic Associations with COVID-19”  b) Methods, section of “Genetic Associations with Polyunsaturated Fatty Acids”; section of “Genetic Associations with COVID-19”  c) Methods, section of “Mendelian Randomization Analyses”  d) NA  e) Methods, section of “Mendelian Randomization Analyses” |
| 1. **Assessment of assumptions: Describe any methods used to assess the assumptions or justify their validity.** | Methods, section of “Mendelian Randomization Analyses” |
| 1. **Sensitivity analyses:** Describe any sensitivity analyses or additional analyses performed. | Methods, section of “Mendelian Randomization Analyses” |
| 1. **Software and pre-registration**   a) Name statistical software and package(s), including version and settings used.  b) State whether the study protocol and details were pre-registered (as well as when and where). | a) Methods, section of section of “Mendelian Randomization Analyses”  b) Data Available Statement |
| **Results** |  |
| 1. **Descriptive data**   a) Report the numbers of individuals at each stage of included studies and reasons for exclusion. Consider use of a flow-diagram.  b) Report summary statistics for phenotypic exposure(s), outcome(s) and other relevant variables (e.g. means, standard deviations, proportions).  c) If the data sources include meta-analyses of previous studies, provide the number of studies, their reported ancestry, if available, and assessments of heterogeneity across these studies. Consider using a supplementary table for each data source.  d) For two-sample Mendelian randomization:  i. Provide information on the similarity of the genetic variant-exposure associations between the exposure and outcome samples.  ii. Provide information on extent of sample overlap between the exposure and outcome data sources. | a) Results, section of “Bidirectional Mendelian Randomization Analyses”  b) Results, section of “Bidirectional Mendelian Randomization Analyses”; Supplementary Tables 4-5  c) Results, section of “Bidirectional Mendelian Randomization Analyses”  d) Results, section of “Bidirectional Mendelian Randomization Analyses” |
| 1. **Main results**   a) Report the associations between genetic variant and exposure, and between genetic variant and outcome, preferably on an interpretable scale (e.g. comparing 25th and 75th percentile of allele count or genetic risk score, if individual-level data available).  b) Report causal effect estimate between exposure and outcome, and the measures of uncertainty from the MR analysis. Use an intuitive scale, such as odds ratio, or relative  risk, per standard deviation difference.  c) If relevant, consider translating estimates of relative risk into absolute risk for a meaningful time-period.  d) Consider any plots to visualize results (e.g. forest plot, scatterplot of associations between genetic variants and outcome versus between genetic variants and exposure). | a) Results, section of “Bidirectional Mendelian Randomization Analyses”  b) Results, section of “Bidirectional Mendelian Randomization Analyses”; Supplementary Tables 6-29  c) NA  d) Figures 2-3 |
| 1. **Assessment of assumptions**   a) Assess the validity of the assumptions.  b) Report any additional statistics (e.g., assessments of heterogeneity, such as I2, Q statistic). | a) Results, section of “Bidirectional Mendelian Randomization Analyses”; Supplementary Tables 6-29  b) Results, section of “Bidirectional Mendelian Randomization Analyses”; Supplementary Tables 6-29 |
| 1. **Sensitivity and additional analyses**   a) Use sensitivity analyses to assess the robustness of the main results to violations of the assumptions.  b) Report results from other sensitivity analyses (e.g., replication study with different dataset, analyses of subgroups, validation of instrument(s), simulations, etc.).  c) Report any assessment of direction of causality (e.g., bidirectional MR).  d) When relevant, report and compare with estimates from non-MR analyses.  e) Consider any additional plots to visualize results (e.g., leave-one-out analyses). | a) Results, section of “Bidirectional Mendelian Randomization Analyses”; Supplementary Tables 6-31  b) Results, section of “Bidirectional Mendelian Randomization Analyses”; Supplementary Tables 6-31  c) Results, section of “Bidirectional Mendelian Randomization Analyses”; Supplementary Tables 14-29  d) Results, section of “Observational Association Analysis”; Tables 2-3  e) Figures 2-3 |
| **Discussion** |  |
| 1. **Key results** | Discussion, paragraph 1 |
| 1. **Limitations**   Discuss limitations of the study, taking into account the validity of the MR assumptions, other sources of potential bias, and imprecision. Discuss both direction and magnitude of any potential bias, and any efforts to address them. | Discussion, paragraph 5 |
| 1. **Interpretations**   a) Give a cautious overall interpretation of results considering objectives and limitations. Compare with results from other relevant studies.  b) Discuss underlying biological mechanisms that could be modelled by using the genetic variants to assess the relationship between the exposure and the outcome.  c) Discuss whether the results have clinical or policy relevance, and whether interventions could have the same size effect. | a) Discussion, paragraphs 2-3  b) Discussion, paragraphs 2-3  c) Discussion, paragraph 2 |
| 1. **Generalizability:** | Discussion, paragraph 5 |
| 1. **Funding:** | Funding |
| 1. **Data and data sharing:** | Data Available Statement |
| 1. **Conflicts of Interest:** | Conflicts of Interest |

**Supplementary Table 4.** Genetic instruments for plasma polyunsaturated fatty acids.

| **PUFA** | **SNP** | **Chr** | **Position (BP)** | **Nearest gene** | **Effect allele** | **Non effect allele** | **EAF** | **Beta** | **SE** | **P-value** |
| --- | --- | --- | --- | --- | --- | --- | --- | --- | --- | --- |
| ALA | rs174547 | 11 | 61570783 | *FADS1* | C | T | 0.33124 | 0.016 | 0.001 | 3.50×10^−641^ |
| LA | rs10740118 | 10 | 65101207 | *JMJD1C* | C | G | 0.407725 | -0.248 | 0.043 | 8.10×10^−9^ |
| LA | rs174547 | 11 | 61570783 | *FADS1* | C | T | 0.33124 | 1.474 | 0.042 | 5.00×10^−274^ |
| LA | rs16966952 | 16 | 15135943 | *NTAN1* | A | G | 0.279871 | -0.351 | 0.044 | 1.20×10^−15^ |
| GLA | rs174547 | 11 | 61570783 | *FADS1* | C | T | 0.33124 | -0.016 | 0.001 | 2.30×10^−72^ |
| GLA | rs16966952 | 16 | 15135943 | *PDXDC1* | A | G | 0.279871 | -0.0061 | 0.001 | 5.00×10^−11^ |
| DGLA | rs174547 | 11 | 61570783 | *FADS1* | C | T | 0.33124 | 0.36 | 0.01 | 2.60×10^−151^ |
| DGLA | rs16966952 | 16 | 15135943 | *PDXDC1* | A | G | 0.279871 | -0.22 | 0.02 | 7.60×10^−15^ |
| AA | rs174547 | 11 | 61570783 | *FADS1* | C | T | 0.33124 | -1.691 | 0.025 | 3×10^−971^ |
| AA | rs16966952 | 16 | 15135943 | *NTAN1* | A | G | 0.279871 | -0.199 | 0.031 | 2.40×10^−10^ |
| DPA-n3 | rs780094 | 2 | 27741237 | *GCKR* | T | C | 0.447679 | 0.017 | 0.003 | 9.04×10^−9^ |
| DPA-n3 | rs3734398 | 6 | 10982973 | *ELOVL2* | C | T | 0.434361 | 0.04 | 0.003 | 9.60×10^−44^ |
| DPA-n3 | rs174547 | 11 | 61570783 | *FADS1* | T | C | 0.66876 | 0.075 | 0.003 | 3.80×10^−154^ |
| DHA | rs2236212 | 6 | 10995015 | *ELOVL2* | G | C | 0.569 | 0.113 | 0.014 | 1.30×10^−15^ |

PUFA, polyunsaturated fatty acid; ALA, α-linolenic acid; LA, linoleic acid; GLA, γ-linolenic acid; DGLA, dihomo-γ-linolenic acid; AA, arachidonic acid; DPA-n3, docosapentaenoic acid; DHA, docosahexaenoic acid; SNP, single nucleotide polymorphism; Chr, chromosome; EAF, Effect allele frequency; SE, standard error. For each PUFA, we selected SNPs that reached genome-wide significance level (P < 5 × 10^−8^) and were restricted by linkage disequilibrium (LD) clumping to ensure independence (R^2^ < 0.001 within a 10 Mb window).

**Supplementary Table 5.** Significant SNPs for red blood cell polyunsaturated fatty acids.

| **PUFA** | **SNP** | **Chr** | **Position (BP)** | **Nearest gene** | **Effect allele** | **Non effect allele** | **EAF** | **Beta** | **SE** | **P-value** |
| --- | --- | --- | --- | --- | --- | --- | --- | --- | --- | --- |
| ALA | rs174548 | 11 | 61571348 | *FADS1* | C | G | 0.69996 | -0.031079453 | 0.005544021 | 2.10×10^−8^ |
| ALA | rs1535 | 11 | 61597972 | *FADS2* | A | G | 0.66595 | -0.030344391 | 0.005554751 | 4.70×10^−8^ |
| ALA | rs174574 | 11 | 61600342 | *FADS2* | C | A | 0.63796 | -0.030961912 | 0.005666031 | 4.60×10^−8^ |
| LA | rs174550 | 11 | 61571478 | *FADS1* | T | C | 0.67143 | -0.025923175 | 0.001983862 | <1×10^−16^ |
| LA | rs174547 | 11 | 61570783 | *FADS1* | T | C | 0.67136 | -0.025924923 | 0.001983583 | <1×10^−16^ |
| LA | rs174546 | 11 | 61569830 | *FADS1* | C | T | 0.67116 | -0.02592704 | 0.001984449 | <1×10^−16^ |
| LA | rs174545 | 11 | 61569306 | *FADS1* | C | G | 0.6711 | -0.025927951 | 0.001984711 | <1×10^−16^ |
| LA | rs174541 | 11 | 61565908 |  | T | C | 0.65148 | -0.026187705 | 0.002005041 | <1×10^−16^ |
| LA | rs4246215 | 11 | 61564299 | *FEN1* | G | T | 0.65088 | -0.02618057 | 0.002007268 | <1×10^−16^ |
| LA | rs174549 | 11 | 61571382 | *FADS1* | G | A | 0.70775 | -0.026380702 | 0.002034896 | <1×10^−16^ |
| LA | rs174555 | 11 | 61579760 | *FADS1* | T | C | 0.70764 | -0.026513764 | 0.002047489 | <1×10^−16^ |
| LA | rs174556 | 11 | 61580635 | *FADS1* | C | T | 0.70764 | -0.026518518 | 0.002048022 | <1×10^−16^ |
| LA | rs174548 | 11 | 61571348 | *FADS1* | C | G | 0.69996 | -0.026169921 | 0.0020244 | <1×10^−16^ |
| LA | rs1535 | 11 | 61597972 | *FADS2* | A | G | 0.66595 | -0.02613899 | 0.002025075 | <1×10^−16^ |
| LA | rs174537 | 11 | 61552680 | *C11orf9* | G | T | 0.66488 | -0.025442966 | 0.00200945 | <1×10^−16^ |
| LA | rs174576 | 11 | 61603510 | *FADS2* | C | A | 0.65841 | -0.025850504 | 0.0020428 | <1×10^−16^ |
| LA | rs174535 | 11 | 61551356 | *C11orf9* | T | C | 0.65839 | -0.025365057 | 0.002007878 | <1×10^−16^ |
| LA | rs174536 | 11 | 61551927 | *C11orf9* | A | C | 0.66386 | -0.025322394 | 0.002009214 | <1×10^−16^ |
| LA | rs174577 | 11 | 61604814 | *FADS2* | C | A | 0.6524 | -0.025577242 | 0.002040915 | <1×10^−16^ |
| LA | rs174574 | 11 | 61600342 | *FADS2* | C | A | 0.63796 | -0.025861826 | 0.002072471 | <1×10^−16^ |
| LA | rs174578 | 11 | 61605499 | *FADS2* | T | A | 0.65154 | -0.025095132 | 0.002039835 | <1×10^−16^ |
| LA | rs102275 | 11 | 61557803 | *C11orf10* | T | C | 0.62686 | -0.02551658 | 0.002076206 | <1×10^−16^ |
| LA | rs174583 | 11 | 61609750 | *FADS2* | C | T | 0.65299 | -0.025052255 | 0.002052984 | <1×10^−16^ |
| LA | rs174538 | 11 | 61560081 | *C11orf10* | G | A | 0.69487 | -0.024581334 | 0.002023435 | <1×10^−16^ |
| LA | rs174528 | 11 | 61543499 | *C11orf9* | T | C | 0.61669 | -0.023670615 | 0.002054035 | <1×10^−16^ |
| LA | rs174601 | 11 | 61623140 | *FADS2* | C | T | 0.61373 | -0.024275521 | 0.002151782 | <1×10^−16^ |
| LA | rs108499 | 11 | 61547237 | *C11orf9* | C | T | 0.67142 | -0.021955949 | 0.002013526 | <1×10^−16^ |
| LA | rs174534 | 11 | 61549458 | *C11orf9* | A | G | 0.66316 | -0.020878982 | 0.001958043 | <1×10^−16^ |
| LA | rs174575 | 11 | 61602003 | *FADS2* | C | G | 0.7332 | -0.02085927 | 0.002270508 | <1×10^−16^ |
| LA | rs174593 | 11 | 61618831 | *FADS2* | T | C | 0.7208 | -0.019803494 | 0.002267427 | <1×10^−16^ |
| LA | rs174579 | 11 | 61605613 | *FADS2* | C | T | 0.78402 | -0.019710352 | 0.002352998 | <1×10^−16^ |
| LA | rs174591 | 11 | 61617676 | *FADS2* | T | A | 0.71832 | -0.019329175 | 0.002308087 | <1×10^−16^ |
| LA | rs968567 | 11 | 61595564 | *FADS2* | C | T | 0.83527 | -0.022516292 | 0.002717439 | 1.10×10^−16^ |
| LA | rs174585 | 11 | 61611694 | *FADS2* | G | A | 0.78304 | -0.019280656 | 0.002340284 | 2.20×10^−16^ |
| LA | rs174448 | 11 | 61639573 |  | A | G | 0.64303 | -0.01629102 | 0.002011707 | 5.60×10^−16^ |
| LA | rs174570 | 11 | 61597212 | *FADS2* | C | T | 0.86422 | -0.023734742 | 0.002960526 | 1.10×10^−15^ |
| LA | rs174589 | 11 | 61615803 | *FADS2* | C | G | 0.78765 | -0.018654832 | 0.002330472 | 1.20×10^−15^ |
| LA | rs422249 | 11 | 61639488 |  | C | T | 0.67098 | -0.016220448 | 0.002071517 | 4.90×10^−15^ |
| LA | rs174605 | 11 | 61626921 | *FADS2* | G | T | 0.71151 | -0.016218161 | 0.002074222 | 5.30×10^−15^ |
| LA | rs174597 | 11 | 61621040 | *FADS2* | G | C | 0.74735 | -0.018547979 | 0.002373894 | 5.60×10^−15^ |
| LA | rs174611 | 11 | 61627881 | *FADS2* | T | C | 0.71039 | -0.01626599 | 0.002083075 | 5.80×10^−15^ |
| LA | rs174449 | 11 | 61640379 |  | A | G | 0.63271 | -0.015007949 | 0.001941676 | 1.10×10^−14^ |
| LA | rs509360 | 11 | 61548559 | *C11orf9* | G | A | 0.649 | 0.019146856 | 0.002509696 | 2.40×10^−14^ |
| LA | rs2269928 | 11 | 61537529 | *C11orf9* | T | G | 0.77978 | -0.020839115 | 0.002805667 | 1.10×10^−13^ |
| LA | rs2727270 | 11 | 61603237 | *FADS2* | C | T | 0.89029 | -0.022241527 | 0.002996633 | 1.20×10^−13^ |
| LA | rs2727271 | 11 | 61603358 | *FADS2* | A | T | 0.89 | -0.022240948 | 0.002998407 | 1.20×10^−13^ |
| LA | rs174627 | 11 | 61637466 |  | G | A | 0.835 | -0.019385566 | 0.002750477 | 1.80×10^−12^ |
| LA | rs2524299 | 11 | 61604782 | *FADS2* | A | T | 0.88064 | -0.021077959 | 0.003019821 | 3.00×10^−12^ |
| LA | rs174479 | 11 | 61678754 | *RAB3IL1* | C | G | 0.81614 | -0.019966658 | 0.002868303 | 3.40×10^−12^ |
| LA | rs2845573 | 11 | 61601908 | *FADS2* | A | G | 0.92668 | -0.025076609 | 0.003623849 | 4.50×10^−12^ |
| LA | rs2072114 | 11 | 61605215 | *FADS2* | A | G | 0.8814 | -0.020285664 | 0.002962778 | 7.50×10^−12^ |
| LA | rs174455 | 11 | 61656117 | *FADS3* | G | A | 0.36538 | 0.013278008 | 0.001944147 | 8.50×10^−12^ |
| LA | rs2851682 | 11 | 61616012 | *FADS2* | A | G | 0.92129 | -0.026018 | 0.003862008 | 1.60×10^−11^ |
| LA | rs174616 | 11 | 61629122 | *FADS2* | G | A | 0.52756 | -0.012926856 | 0.001927302 | 2.00×10^−11^ |
| LA | rs174532 | 11 | 61548874 | *C11orf9* | G | A | 0.72114 | 0.017458535 | 0.002616894 | 2.50×10^−11^ |
| LA | rs174626 | 11 | 61637057 |  | A | G | 0.52886 | -0.01304876 | 0.001959376 | 2.70×10^−11^ |
| LA | rs174450 | 11 | 61641542 | *FADS3* | G | T | 0.47314 | 0.013061701 | 0.001993513 | 5.70×10^−11^ |
| LA | rs198462 | 11 | 61524119 | *C11orf9* | G | A | 0.48644 | 0.012838099 | 0.001984777 | 9.90×10^−11^ |
| LA | rs198476 | 11 | 61525730 | *C11orf9* | G | A | 0.47167 | 0.01298316 | 0.002038935 | 1.90×10^−10^ |
| LA | rs198464 | 11 | 61521621 |  | G | A | 0.48411 | 0.012173434 | 0.001915542 | 2.10×10^−10^ |
| LA | rs412334 | 11 | 61560261 | *FEN1* | C | T | 0.85305 | 0.026964394 | 0.004252483 | 2.30×10^−10^ |
| LA | rs174634 | 11 | 61647387 | *FADS3* | C | G | 0.72588 | -0.013223298 | 0.002151382 | 7.90×10^−10^ |
| LA | rs174464 | 11 | 61657926 | *FADS3* | G | A | 0.72982 | -0.013261427 | 0.002186526 | 1.30×10^−9^ |
| LA | rs1000778 | 11 | 61655305 | *FADS3* | G | A | 0.72904 | -0.012692579 | 0.00211719 | 2.00×10^−9^ |
| LA | rs174456 | 11 | 61656182 | *FADS3* | A | C | 0.72918 | -0.012665509 | 0.002115463 | 2.10×10^−9^ |
| LA | rs2526678 | 11 | 61623793 | *FADS2* | G | A | 0.93057 | -0.026337496 | 0.004428129 | 2.70×10^−9^ |
| LA | rs149803 | 11 | 61539020 | *C11orf9* | C | G | 0.74636 | 0.015625177 | 0.002763177 | 1.60×10^−8^ |
| LA | rs12580543 | 12 | 7100973 | *MBOAT5* | A | C | 0.93596 | -0.045842834 | 0.0039947 | <1×10^−16^ |
| LA | rs4394918 | 12 | 7110335 | *MBOAT5* | C | A | 0.93926 | -0.04537712 | 0.004016596 | <1×10^−16^ |
| LA | rs16928105 | 12 | 7114196 | *MBOAT5* | C | T | 0.93917 | -0.045109814 | 0.00400763 | <1×10^−16^ |
| LA | rs1984564 | 12 | 7090193 | *MBOAT5* | A | G | 0.91021 | -0.036294608 | 0.003302183 | <1×10^−16^ |
| LA | rs7952839 | 12 | 7110571 | *MBOAT5* | G | A | 0.90923 | -0.036105147 | 0.003312813 | <1×10^−16^ |
| LA | rs12582990 | 12 | 7145853 |  | T | C | 0.90538 | -0.037264689 | 0.003452779 | <1×10^−16^ |
| LA | rs16933023 | 12 | 7107348 | *MBOAT5* | C | G | 0.9166 | -0.037045099 | 0.003512866 | <1×10^−16^ |
| LA | rs12580233 | 12 | 7124522 | *MBOAT5* | T | C | 0.91542 | -0.036368727 | 0.003485457 | <1×10^−16^ |
| LA | rs16933011 | 12 | 7073805 |  | G | T | 0.91316 | -0.036528813 | 0.003541195 | <1×10^−16^ |
| LA | rs3764031 | 12 | 7091918 | *MBOAT5* | C | T | 0.91377 | -0.035061406 | 0.003409011 | <1×10^−16^ |
| LA | rs7311050 | 12 | 7120836 | *MBOAT5* | C | T | 0.91388 | -0.03495796 | 0.003409288 | <1×10^−16^ |
| LA | rs2110073 | 12 | 7075882 | *PHB2* | C | T | 0.89587 | -0.031198306 | 0.003100397 | <1×10^−16^ |
| LA | rs7962738 | 12 | 7166672 | *C1S* | T | A | 0.94463 | -0.040285903 | 0.004311937 | <1×10^−16^ |
| LA | rs12579775 | 12 | 7085171 |  | G | A | 0.90521 | -0.030763165 | 0.003293403 | <1×10^−16^ |
| LA | rs759052 | 12 | 7069620 | *PTPN6* | C | T | 0.8603 | -0.029867787 | 0.003337284 | <1×10^−16^ |
| LA | rs11064497 | 12 | 7169661 | *C1S* | C | T | 0.86151 | -0.017528017 | 0.002841673 | 6.90×10^−10^ |
| LA | rs12368181 | 12 | 7181105 |  | A | G | 0.86823 | -0.017538707 | 0.002862766 | 9.00×10^−10^ |
| LA | rs12366520 | 12 | 7181162 |  | C | A | 0.8682 | -0.017532536 | 0.002863014 | 9.10×10^−10^ |
| LA | rs12146727 | 12 | 7170336 | *C1S* | G | A | 0.86979 | -0.017143651 | 0.002814623 | 1.10×10^−9^ |
| LA | rs16933078 | 12 | 7171338 | *C1S* | T | A | 0.86831 | -0.017158756 | 0.002818129 | 1.10×10^−9^ |
| LA | rs16933084 | 12 | 7172084 | *C1S* | A | T | 0.86906 | -0.017078607 | 0.002814758 | 1.30×10^−9^ |
| LA | rs11838267 | 12 | 7175872 | *C1S* | T | C | 0.86788 | -0.017089309 | 0.002813916 | 1.30×10^−9^ |
| LA | rs12368783 | 12 | 7179400 |  | G | T | 0.86858 | -0.017239953 | 0.002841371 | 1.30×10^−9^ |
| LA | rs7962629 | 12 | 7166770 | *C1S* | A | G | 0.86456 | -0.017224081 | 0.002842693 | 1.40×10^−9^ |
| LA | rs11064501 | 12 | 7179822 |  | A | C | 0.86691 | -0.017250584 | 0.002845788 | 1.30×10^−9^ |
| LA | rs10849546 | 12 | 7176204 | *C1S* | G | A | 0.86876 | -0.01702788 | 0.002813537 | 1.40×10^−9^ |
| LA | rs12371227 | 12 | 7176978 | *C1S* | C | T | 0.86834 | -0.017016845 | 0.002810648 | 1.40×10^−9^ |
| LA | rs7183 | 12 | 7178019 | *C1S* | G | T | 0.86851 | -0.017045684 | 0.002816664 | 1.40×10^−9^ |
| LA | rs11064498 | 12 | 7171507 | *C1S* | A | G | 0.85324 | -0.016734174 | 0.002842967 | 4.00×10^−9^ |
| LA | rs3919533 | 12 | 7162801 |  | T | C | 0.83695 | -0.015492514 | 0.002653374 | 5.30×10^−9^ |
| LA | rs7311672 | 12 | 7165114 |  | G | A | 0.83872 | -0.014988034 | 0.002629557 | 1.20×10^−8^ |
| GLA | rs174548 | 11 | 61571348 | *FADS1* | C | G | 0.69996 | 0.056093204 | 0.008052263 | 3.30×10^−12^ |
| GLA | rs174549 | 11 | 61571382 | *FADS1* | G | A | 0.70775 | 0.05399605 | 0.008102706 | 2.70×10^−11^ |
| GLA | rs174555 | 11 | 61579760 | *FADS1* | T | C | 0.70764 | 0.054235078 | 0.008152221 | 2.90×10^−11^ |
| GLA | rs174556 | 11 | 61580635 | *FADS1* | C | T | 0.70764 | 0.054246393 | 0.008154209 | 2.90×10^−11^ |
| GLA | rs4246215 | 11 | 61564299 | *FEN1* | G | T | 0.65088 | 0.051586938 | 0.008006419 | 1.20×10^−11^ |
| GLA | rs174541 | 11 | 61565908 |  | T | C | 0.65148 | 0.051462442 | 0.007998025 | 1.20×10^−10^ |
| GLA | rs174535 | 11 | 61551356 | *C11orf9* | T | C | 0.65839 | 0.051333021 | 0.007993626 | 1.30×10^−10^ |
| GLA | rs102275 | 11 | 61557803 | *C11orf10* | T | C | 0.62686 | 0.052391657 | 0.008240345 | 2.00×10^−10^ |
| GLA | rs174574 | 11 | 61600342 | *FADS2* | C | A | 0.63796 | 0.05204465 | 0.00824537 | 2.80×10^−10^ |
| GLA | rs1535 | 11 | 61597972 | *FADS2* | A | G | 0.66595 | 0.050795864 | 0.008081966 | 3.30×10^−10^ |
| GLA | rs174577 | 11 | 61604814 | *FADS2* | C | A | 0.6524 | 0.050837053 | 0.008124732 | 3.90×10^−10^ |
| GLA | rs174578 | 11 | 61605499 | *FADS2* | T | A | 0.65154 | 0.050440978 | 0.008113275 | 5.10×10^−10^ |
| GLA | rs174536 | 11 | 61551927 | *C11orf9* | A | C | 0.66386 | 0.049696809 | 0.008001506 | 5.30×10^−10^ |
| GLA | rs174537 | 11 | 61552680 | *C11orf9* | G | T | 0.66488 | 0.049704656 | 0.008006979 | 5.40×10^−10^ |
| GLA | rs174550 | 11 | 61571478 | *FADS1* | T | C | 0.67143 | 0.049090576 | 0.007921297 | 5.70×10^−10^ |
| GLA | rs174545 | 11 | 61569306 | *FADS1* | C | G | 0.6711 | 0.049037087 | 0.007925609 | 6.10×10^−10^ |
| GLA | rs174546 | 11 | 61569830 | *FADS1* | C | T | 0.67116 | 0.049019012 | 0.007924646 | 6.20×10^−10^ |
| GLA | rs174547 | 11 | 61570783 | *FADS1* | T | C | 0.67136 | 0.048973853 | 0.007921437 | 6.30×10^−10^ |
| GLA | rs174576 | 11 | 61603510 | *FADS2* | C | A | 0.65841 | 0.050172662 | 0.008139624 | 7.10×10^−10^ |
| GLA | rs174583 | 11 | 61609750 | *FADS2* | C | T | 0.65299 | 0.049850145 | 0.00816299 | 1.00×10^−9^ |
| GLA | rs174528 | 11 | 61543499 | *C11orf9* | T | C | 0.61669 | 0.049271281 | 0.008126065 | 1.30×10^−9^ |
| DGLA | rs174555 | 11 | 61579760 | *FADS1* | T | C | 0.70764 | -0.093383417 | 0.002276368 | <1×10^−16^ |
| DGLA | rs174556 | 11 | 61580635 | *FADS1* | C | T | 0.70764 | -0.093407652 | 0.002276937 | <1×10^−16^ |
| DGLA | rs174549 | 11 | 61571382 | *FADS1* | G | A | 0.70775 | -0.092786816 | 0.002263023 | <1×10^−16^ |
| DGLA | rs174548 | 11 | 61571348 | *FADS1* | C | G | 0.69996 | -0.092279425 | 0.002252042 | <1×10^−16^ |
| DGLA | rs174538 | 11 | 61560081 | *C11orf10* | G | A | 0.69487 | -0.089612614 | 0.002277243 | <1×10^−16^ |
| DGLA | rs174545 | 11 | 61569306 | *FADS1* | C | G | 0.6711 | -0.087925132 | 0.002250125 | <1×10^−16^ |
| DGLA | rs174550 | 11 | 61571478 | *FADS1* | T | C | 0.67143 | -0.087877368 | 0.002249418 | <1×10^−16^ |
| DGLA | rs174546 | 11 | 61569830 | *FADS1* | C | T | 0.67116 | -0.087912789 | 0.002249869 | <1×10^−16^ |
| DGLA | rs174547 | 11 | 61570783 | *FADS1* | T | C | 0.67136 | -0.087868043 | 0.002249103 | <1×10^−16^ |
| DGLA | rs1535 | 11 | 61597972 | *FADS2* | A | G | 0.66595 | -0.089394544 | 0.002298171 | <1×10^−16^ |
| DGLA | rs174537 | 11 | 61552680 | *C11orf9* | G | T | 0.66488 | -0.088477735 | 0.0022804 | <1×10^−16^ |
| DGLA | rs174536 | 11 | 61551927 | *C11orf9* | A | C | 0.66386 | -0.088243053 | 0.002282768 | <1×10^−16^ |
| DGLA | rs174535 | 11 | 61551356 | *C11orf9* | T | C | 0.65839 | -0.08813322 | 0.00228418 | <1×10^−16^ |
| DGLA | rs174541 | 11 | 61565908 |  | T | C | 0.65148 | -0.08718654 | 0.002300447 | <1×10^−16^ |
| DGLA | rs4246215 | 11 | 61564299 | *FEN1* | G | T | 0.65088 | -0.087265838 | 0.002302899 | <1×10^−16^ |
| DGLA | rs174574 | 11 | 61600342 | *FADS2* | C | A | 0.63796 | -0.089774642 | 0.002372438 | <1×10^−16^ |
| DGLA | rs174577 | 11 | 61604814 | *FADS2* | C | A | 0.6524 | -0.088450038 | 0.002337515 | <1×10^−16^ |
| DGLA | rs174576 | 11 | 61603510 | *FADS2* | C | A | 0.65841 | -0.088521769 | 0.002340525 | <1×10^−16^ |
| DGLA | rs102275 | 11 | 61557803 | *C11orf10* | T | C | 0.62686 | -0.089486222 | 0.002382655 | <1×10^−16^ |
| DGLA | rs174578 | 11 | 61605499 | *FADS2* | T | A | 0.65154 | -0.08766831 | 0.002343702 | <1×10^−16^ |
| DGLA | rs174583 | 11 | 61609750 | *FADS2* | C | T | 0.65299 | -0.087686767 | 0.002364368 | <1×10^−16^ |
| DGLA | rs108499 | 11 | 61547237 | *C11orf9* | C | T | 0.67142 | -0.083095987 | 0.002342602 | <1×10^−16^ |
| DGLA | rs174528 | 11 | 61543499 | *C11orf9* | T | C | 0.61669 | -0.084062886 | 0.002413168 | <1×10^−16^ |
| DGLA | rs174534 | 11 | 61549458 | *C11orf9* | A | G | 0.66316 | -0.079069068 | 0.002300709 | <1×10^−16^ |
| DGLA | rs968567 | 11 | 61595564 | *FADS2* | C | T | 0.83527 | -0.108510323 | 0.003158099 | <1×10^−16^ |
| DGLA | rs174601 | 11 | 61623140 | *FADS2* | C | T | 0.61373 | -0.084828533 | 0.002568194 | <1×10^−16^ |
| DGLA | rs174579 | 11 | 61605613 | *FADS2* | C | T | 0.78402 | -0.08787706 | 0.00282469 | <1×10^−16^ |
| DGLA | rs174585 | 11 | 61611694 | *FADS2* | G | A | 0.78304 | -0.086669437 | 0.002816734 | <1×10^−16^ |
| DGLA | rs174575 | 11 | 61602003 | *FADS2* | C | G | 0.7332 | -0.083882013 | 0.002750669 | <1×10^−16^ |
| DGLA | rs174589 | 11 | 61615803 | *FADS2* | C | G | 0.78765 | -0.084379029 | 0.002825277 | <1×10^−16^ |
| DGLA | rs174591 | 11 | 61617676 | *FADS2* | T | A | 0.71832 | -0.080976985 | 0.002834895 | <1×10^−16^ |
| DGLA | rs174593 | 11 | 61618831 | *FADS2* | T | C | 0.7208 | -0.077790899 | 0.002813594 | <1×10^−16^ |
| DGLA | rs174597 | 11 | 61621040 | *FADS2* | G | C | 0.74735 | -0.079166265 | 0.002959683 | <1×10^−16^ |
| DGLA | rs174627 | 11 | 61637466 |  | G | A | 0.835 | -0.081359634 | 0.003515982 | <1×10^−16^ |
| DGLA | rs2269928 | 11 | 61537529 | *C11orf9* | T | G | 0.77978 | -0.080790453 | 0.003623491 | <1×10^−16^ |
| DGLA | rs509360 | 11 | 61548559 | *C11orf9* | G | A | 0.649 | 0.070454333 | 0.003264146 | <1×10^−16^ |
| DGLA | rs174611 | 11 | 61627881 | *FADS2* | T | C | 0.71039 | -0.057047069 | 0.002721521 | <1×10^−16^ |
| DGLA | rs174605 | 11 | 61626921 | *FADS2* | G | T | 0.71151 | -0.056782086 | 0.002710525 | <1×10^−16^ |
| DGLA | rs174448 | 11 | 61639573 |  | A | G | 0.64303 | -0.052877561 | 0.002655524 | <1×10^−16^ |
| DGLA | rs422249 | 11 | 61639488 |  | C | T | 0.67098 | -0.053451571 | 0.00273317 | <1×10^−16^ |
| DGLA | rs174449 | 11 | 61640379 |  | A | G | 0.63271 | -0.050106436 | 0.002567625 | <1×10^−16^ |
| DGLA | rs174469 | 11 | 61667443 | *RAB3IL1* | C | T | 0.82555 | -0.067713817 | 0.00367945 | <1×10^−16^ |
| DGLA | rs174479 | 11 | 61678754 | *RAB3IL1* | C | G | 0.81614 | -0.06773423 | 0.003816048 | <1×10^−16^ |
| DGLA | rs174634 | 11 | 61647387 | *FADS3* | C | G | 0.72588 | -0.050487213 | 0.00286443 | <1×10^−16^ |
| DGLA | rs526126 | 11 | 61624885 | *FADS2* | C | G | 0.76593 | -0.072444969 | 0.004129038 | <1×10^−16^ |
| DGLA | rs174464 | 11 | 61657926 | *FADS3* | G | A | 0.72982 | -0.04998859 | 0.002920249 | <1×10^−16^ |
| DGLA | rs1000778 | 11 | 61655305 | *FADS3* | G | A | 0.72904 | -0.048000579 | 0.002829881 | <1×10^−16^ |
| DGLA | rs174456 | 11 | 61656182 | *FADS3* | A | C | 0.72918 | -0.047889577 | 0.002828034 | <1×10^−16^ |
| DGLA | rs174532 | 11 | 61548874 | *C11orf9* | G | A | 0.72114 | 0.058842315 | 0.003511758 | <1×10^−16^ |
| DGLA | rs174455 | 11 | 61656117 | *FADS3* | G | A | 0.36538 | 0.042120936 | 0.002625943 | <1×10^−16^ |
| DGLA | rs412334 | 11 | 61560261 | *FEN1* | C | T | 0.85305 | 0.084373277 | 0.005760646 | <1×10^−16^ |
| DGLA | rs174616 | 11 | 61629122 | *FADS2* | G | A | 0.52756 | -0.038302878 | 0.002618867 | <1×10^−16^ |
| DGLA | rs174626 | 11 | 61637057 |  | A | G | 0.52886 | -0.038773801 | 0.002662012 | <1×10^−16^ |
| DGLA | rs174450 | 11 | 61641542 | *FADS3* | G | T | 0.47314 | 0.038438145 | 0.002713908 | <1×10^−16^ |
| DGLA | rs149803 | 11 | 61539020 | *C11orf9* | C | G | 0.74636 | 0.051444004 | 0.003767124 | <1×10^−16^ |
| DGLA | rs174570 | 11 | 61597212 | *FADS2* | C | T | 0.86422 | -0.054262313 | 0.004080799 | <1×10^−16^ |
| DGLA | rs198462 | 11 | 61524119 | *C11orf9* | G | A | 0.48644 | 0.032261333 | 0.00275405 | <1×10^−16^ |
| DGLA | rs198464 | 11 | 61521621 |  | G | A | 0.48411 | 0.030068552 | 0.002661756 | <1×10^−16^ |
| DGLA | rs7102974 | 11 | 61560035 | *C11orf10* | C | T | 0.97886 | -0.125814384 | 0.011223658 | <1×10^−16^ |
| DGLA | rs198476 | 11 | 61525730 | *C11orf9* | G | A | 0.47167 | 0.031434935 | 0.002835967 | <1×10^−16^ |
| DGLA | rs498793 | 11 | 61624705 | *FADS2* | C | T | 0.60008 | 0.045940115 | 0.004146446 | <1×10^−16^ |
| DGLA | rs174468 | 11 | 61663691 |  | G | A | 0.60365 | 0.034164486 | 0.003117167 | <1×10^−16^ |
| DGLA | rs2845573 | 11 | 61601908 | *FADS2* | A | G | 0.92668 | -0.054916984 | 0.005041632 | <1×10^−16^ |
| DGLA | rs2851682 | 11 | 61616012 | *FADS2* | A | G | 0.92129 | -0.057949708 | 0.00537644 | <1×10^−16^ |
| DGLA | rs198473 | 11 | 61526556 | *C11orf9* | A | G | 0.74785 | -0.035487193 | 0.003376086 | <1×10^−16^ |
| DGLA | rs174476 | 11 | 61674118 | *RAB3IL1* | C | T | 0.60208 | 0.033121705 | 0.00318768 | <1×10^−16^ |
| DGLA | rs174478 | 11 | 61678576 | *RAB3IL1* | T | G | 0.60099 | 0.033154421 | 0.003200503 | <1×10^−16^ |
| DGLA | rs174602 | 11 | 61624414 | *FADS2* | T | C | 0.76774 | -0.045141254 | 0.004359976 | <1×10^−16^ |
| DGLA | rs666870 | 11 | 61677479 | *RAB3IL1* | G | A | 0.6013 | 0.033122844 | 0.003199448 | <1×10^−16^ |
| DGLA | rs198475 | 11 | 61526071 | *C11orf9* | C | T | 0.75215 | -0.034406845 | 0.003339929 | <1×10^−16^ |
| DGLA | rs13966 | 11 | 61664992 | *RAB3IL1* | C | T | 0.54676 | 0.031902864 | 0.003191463 | <1×10^−16^ |
| DGLA | rs2235093 | 11 | 61665122 | *RAB3IL1* | G | A | 0.54967 | 0.031732481 | 0.003194219 | <1×10^−16^ |
| DGLA | rs579383 | 11 | 61536583 | *C11orf9* | A | G | 0.57529 | 0.030046342 | 0.00304175 | <1×10^−16^ |
| DGLA | rs2727270 | 11 | 61603237 | *FADS2* | C | T | 0.89029 | -0.041144884 | 0.004187134 | <1×10^−16^ |
| DGLA | rs2727271 | 11 | 61603358 | *FADS2* | A | T | 0.89 | -0.041055738 | 0.004190331 | <1×10^−16^ |
| DGLA | rs650436 | 11 | 61536430 | *C11orf9* | C | T | 0.57937 | 0.029706454 | 0.003050024 | <1×10^−16^ |
| DGLA | rs2526678 | 11 | 61623793 | *FADS2* | G | A | 0.93057 | -0.059961422 | 0.006165896 | <1×10^−16^ |
| DGLA | rs198446 | 11 | 61503381 | *DAGLA* | G | A | 0.74811 | -0.03169164 | 0.003313523 | <1×10^−16^ |
| DGLA | rs2072114 | 11 | 61605215 | *FADS2* | A | G | 0.8814 | -0.039457728 | 0.004132432 | <1×10^−16^ |
| DGLA | rs1692120 | 11 | 61417472 |  | G | A | 0.56219 | 0.03328641 | 0.003663514 | <1×10^−16^ |
| DGLA | rs174472 | 11 | 61671956 | *RAB3IL1* | G | A | 0.45428 | 0.0295229 | 0.00325842 | <1×10^−16^ |
| DGLA | rs740006 | 11 | 61557868 | *C11orf10* | T | C | 0.91448 | 0.059024656 | 0.006536418 | <1×10^−16^ |
| DGLA | rs1800009 | 11 | 61730234 | *BEST1* | C | T | 0.3478 | 0.026452212 | 0.002951664 | <1×10^−16^ |
| DGLA | rs2524299 | 11 | 61604782 | *FADS2* | A | T | 0.88064 | -0.037770077 | 0.004228786 | <1×10^−16^ |
| DGLA | rs198418 | 11 | 61496272 | *DAGLA* | A | C | 0.73879 | -0.028012495 | 0.003145658 | <1×10^−16^ |
| DGLA | rs198425 | 11 | 61491431 | *DAGLA* | A | T | 0.7406 | -0.027561243 | 0.003129851 | <1×10^−16^ |
| DGLA | rs2453710 | 11 | 61406542 | *RPLP0P2* | A | G | 0.58218 | 0.034261295 | 0.003928726 | <1×10^−16^ |
| DGLA | rs3758977 | 11 | 61737244 | *LOC399900* | G | T | 0.3436 | 0.025007725 | 0.002869771 | <1×10^−16^ |
| DGLA | rs81658 | 11 | 61487944 | *DAGLA* | G | A | 0.74192 | -0.026810422 | 0.003098609 | <1×10^−16^ |
| DGLA | rs198430 | 11 | 61487690 | *DAGLA* | G | A | 0.74192 | -0.026799632 | 0.003098162 | <1×10^−16^ |
| DGLA | rs17633020 | 11 | 61744881 |  | G | A | 0.76205 | -0.026458923 | 0.003117735 | <1×10^−16^ |
| DGLA | rs17185574 | 11 | 61745694 |  | T | C | 0.76159 | -0.026226638 | 0.003098671 | <1×10^−16^ |
| DGLA | rs198432 | 11 | 61484981 | *DAGLA* | C | A | 0.73843 | -0.026339822 | 0.003116762 | <1×10^−16^ |
| DGLA | rs1791785 | 11 | 61442813 |  | C | T | 0.7418 | -0.029047122 | 0.003440297 | <1×10^−16^ |
| DGLA | rs2028062 | 11 | 61745953 |  | A | G | 0.34067 | 0.023546253 | 0.002808945 | <1×10^−16^ |
| DGLA | rs10792320 | 11 | 61746291 |  | C | A | 0.34095 | 0.023443942 | 0.002812546 | 1.10×10^−16^ |
| DGLA | rs198453 | 11 | 61464550 | *DAGLA* | C | T | 0.73648 | -0.027339291 | 0.003359827 | 4.40×10^−16^ |
| DGLA | rs472031 | 11 | 61638420 |  | G | A | 0.90451 | 0.039188093 | 0.004829156 | 4.40×10^−16^ |
| DGLA | rs482548 | 11 | 61633182 | *FADS2* | C | T | 0.90592 | 0.03821008 | 0.004845138 | 3.10×10^−15^ |
| DGLA | rs2521568 | 11 | 61700933 | *RAB3IL1* | G | C | 0.92845 | -0.039959378 | 0.005208729 | 1.70×10^−14^ |
| DGLA | rs3825036 | 11 | 61516476 |  | G | A | 0.81744 | 0.030058234 | 0.003918162 | 1.70×10^−14^ |
| DGLA | rs2727266 | 11 | 61704334 | *RAB3IL1* | A | G | 0.92849 | -0.039999915 | 0.005295094 | 4.20×10^−14^ |
| DGLA | rs2521572 | 11 | 61711475 | *RAB3IL1* | G | T | 0.96168 | -0.050851342 | 0.006866003 | 1.30×10^−13^ |
| DGLA | rs2240287 | 11 | 61505583 | *DAGLA* | G | A | 0.82919 | 0.028707876 | 0.003943812 | 3.40×10^−13^ |
| DGLA | rs2238001 | 11 | 61524507 | *C11orf9* | T | C | 0.8425 | 0.031498283 | 0.004338058 | 3.80×10^−13^ |
| DGLA | rs879486 | 11 | 61475233 | *DAGLA* | C | T | 0.6649 | -0.022341409 | 0.003125355 | 8.80×10^−13^ |
| DGLA | rs9735635 | 11 | 61490880 | *DAGLA* | C | A | 0.83017 | 0.027813027 | 0.003894957 | 9.30×10^−13^ |
| DGLA | rs12274157 | 11 | 61477647 | *DAGLA* | A | C | 0.82525 | 0.027112706 | 0.003823371 | 1.30×10^−12^ |
| DGLA | rs2727261 | 11 | 61712131 | *RAB3IL1* | C | T | 0.90126 | -0.037934632 | 0.005412819 | 2.40×10^−12^ |
| DGLA | rs198435 | 11 | 61483324 | *DAGLA* | G | C | 0.84649 | 0.028753775 | 0.004101929 | 2.40×10^−12^ |
| DGLA | rs4963243 | 11 | 61494327 | *DAGLA* | G | A | 0.84942 | 0.029374573 | 0.004225089 | 3.60×10^−12^ |
| DGLA | rs4423188 | 11 | 61766418 |  | T | A | 0.72099 | -0.021455644 | 0.003103223 | 4.70×10^−12^ |
| DGLA | rs10736716 | 11 | 61765133 |  | G | C | 0.63697 | -0.019460274 | 0.00292327 | 2.80×10^−11^ |
| DGLA | rs569258 | 11 | 61520668 | *MYRF* | T | C | 0.65011 | 0.020504387 | 0.003093989 | 3.40×10^−11^ |
| DGLA | rs174552 | 11 | 61574999 | *FADS1* | C | T | 0.99468 | -0.200839135 | 0.030353866 | 3.70×10^−11^ |
| DGLA | rs11827215 | 11 | 61458595 | *DAGLA* | G | A | 0.83972 | 0.024691446 | 0.003732647 | 3.70×10^−11^ |
| DGLA | rs198746 | 11 | 61376970 |  | G | A | 0.81159 | 0.057831365 | 0.008769685 | 4.30×10^−11^ |
| DGLA | rs4963308 | 11 | 61456426 | *DAGLA* | G | A | 0.83996 | 0.024468091 | 0.003719449 | 4.80×10^−11^ |
| DGLA | rs883724 | 11 | 61457857 | *DAGLA* | C | T | 0.84031 | 0.024428044 | 0.003715425 | 4.90×10^−11^ |
| DGLA | rs1109748 | 11 | 61722645 | *BEST1* | C | A | 0.92539 | -0.036466281 | 0.005670758 | 1.30×10^−10^ |
| DGLA | rs17762402 | 11 | 61553201 | *C11orf9* | G | A | 0.94147 | 0.046143991 | 0.007250444 | 2.00×10^−10^ |
| DGLA | rs4313591 | 11 | 61766569 |  | T | C | 0.62638 | -0.018246733 | 0.002906377 | 3.40×10^−10^ |
| DGLA | rs4963441 | 11 | 61768639 |  | C | G | 0.62575 | -0.01808825 | 0.002903362 | 4.70×10^−10^ |
| DGLA | rs6591657 | 11 | 61434532 |  | G | T | 0.88788 | 0.028180673 | 0.004523613 | 4.70×10^−10^ |
| DGLA | rs7937198 | 11 | 61768973 |  | A | G | 0.62364 | -0.018037627 | 0.002900861 | 5.00×10^−10^ |
| DGLA | rs4963442 | 11 | 61768706 |  | T | C | 0.62445 | -0.018054672 | 0.002905273 | 5.20×10^−10^ |
| DGLA | rs7925523 | 11 | 61442492 |  | G | A | 0.83567 | 0.023437215 | 0.003773306 | 5.30×10^−10^ |
| DGLA | rs198745 | 11 | 61376680 |  | T | C | 0.8253 | 0.055397405 | 0.008959375 | 6.30×10^−10^ |
| DGLA | rs198428 | 11 | 61489705 | *DAGLA* | T | A | 0.61775 | 0.018459334 | 0.003019546 | 9.80×10^−10^ |
| DGLA | rs198426 | 11 | 61490486 | *DAGLA* | C | T | 0.67872 | 0.019104342 | 0.003140967 | 1.20×10^−9^ |
| DGLA | rs11230749 | 11 | 61362978 | *LOC101927495* | C | T | 0.87613 | -0.069231534 | 0.011822452 | 4.70×10^−9^ |
| AA | rs2581624 | 3 | 142633869 |  | G | C | 0.79336 | 0.009754995 | 0.001589181 | 8.30×10^−10^ |
| AA | rs2248811 | 3 | 142606942 | *PCOLCE2* | G | C | 0.78715 | 0.008693928 | 0.001593601 | 4.90×10^−9^ |
| AA | rs174545 | 11 | 61569306 | *FADS1* | C | G | 0.6711 | 0.024581721 | 0.001250655 | <1×10^−16^ |
| AA | rs174546 | 11 | 61569830 | *FADS1* | C | T | 0.67116 | 0.024577777 | 0.001250506 | <1×10^−16^ |
| AA | rs174547 | 11 | 61570783 | *FADS1* | T | C | 0.67136 | 0.024564543 | 0.001250019 | <1×10^−16^ |
| AA | rs174550 | 11 | 61571478 | *FADS1* | T | C | 0.67143 | 0.024550948 | 0.001250319 | <1×10^−16^ |
| AA | rs174537 | 11 | 61552680 | *C11orf9* | G | T | 0.66488 | 0.024690364 | 0.001264599 | <1×10^−16^ |
| AA | rs1535 | 11 | 61597972 | *FADS2* | A | G | 0.66595 | 0.024866869 | 0.00127604 | <1×10^−16^ |
| AA | rs174536 | 11 | 61551927 | *C11orf9* | A | C | 0.66386 | 0.024631536 | 0.001264481 | <1×10^−16^ |
| AA | rs174535 | 11 | 61551356 | *C11orf9* | T | C | 0.65839 | 0.024623865 | 0.001264411 | <1×10^−16^ |
| AA | rs4246215 | 11 | 61564299 | *FEN1* | G | T | 0.65088 | 0.024433841 | 0.001268673 | <1×10^−16^ |
| AA | rs174541 | 11 | 61565908 |  | T | C | 0.65148 | 0.024407299 | 0.001267406 | <1×10^−16^ |
| AA | rs102275 | 11 | 61557803 | *C11orf10* | T | C | 0.62686 | 0.024886779 | 0.001309693 | <1×10^−16^ |
| AA | rs174576 | 11 | 61603510 | *FADS2* | C | A | 0.65841 | 0.024507775 | 0.001290441 | <1×10^−16^ |
| AA | rs174574 | 11 | 61600342 | *FADS2* | C | A | 0.63796 | 0.024829465 | 0.001307902 | <1×10^−16^ |
| AA | rs174577 | 11 | 61604814 | *FADS2* | C | A | 0.6524 | 0.024440279 | 0.001288954 | <1×10^−16^ |
| AA | rs174578 | 11 | 61605499 | *FADS2* | T | A | 0.65154 | 0.024219492 | 0.001288197 | <1×10^−16^ |
| AA | rs174583 | 11 | 61609750 | *FADS2* | C | T | 0.65299 | 0.024199448 | 0.001296296 | <1×10^−16^ |
| AA | rs174555 | 11 | 61579760 | *FADS1* | T | C | 0.70764 | 0.024018629 | 0.001301119 | <1×10^−16^ |
| AA | rs174556 | 11 | 61580635 | *FADS1* | C | T | 0.70764 | 0.024026422 | 0.001301439 | <1×10^−16^ |
| AA | rs174548 | 11 | 61571348 | *FADS1* | C | G | 0.69996 | 0.023714033 | 0.001287432 | <1×10^−16^ |
| AA | rs174549 | 11 | 61571382 | *FADS1* | G | A | 0.70775 | 0.023812139 | 0.001293691 | <1×10^−16^ |
| AA | rs174538 | 11 | 61560081 | *C11orf10* | G | A | 0.69487 | 0.023462625 | 0.001279837 | <1×10^−16^ |
| AA | rs174528 | 11 | 61543499 | *C11orf9* | T | C | 0.61669 | 0.023234781 | 0.001300411 | <1×10^−16^ |
| AA | rs174601 | 11 | 61623140 | *FADS2* | C | T | 0.61373 | 0.023990221 | 0.001361246 | <1×10^−16^ |
| AA | rs108499 | 11 | 61547237 | *C11orf9* | C | T | 0.67142 | 0.021386038 | 0.001279824 | <1×10^−16^ |
| AA | rs174534 | 11 | 61549458 | *C11orf9* | A | G | 0.66316 | 0.020499565 | 0.001244953 | <1×10^−16^ |
| AA | rs174570 | 11 | 61597212 | *FADS2* | C | T | 0.86422 | 0.025409935 | 0.001896779 | <1×10^−16^ |
| AA | rs174575 | 11 | 61602003 | *FADS2* | C | G | 0.7332 | 0.018928681 | 0.00146665 | <1×10^−16^ |
| AA | rs174593 | 11 | 61618831 | *FADS2* | T | C | 0.7208 | 0.018203337 | 0.001464843 | <1×10^−16^ |
| AA | rs174579 | 11 | 61605613 | *FADS2* | C | T | 0.78402 | 0.018337051 | 0.001519732 | <1×10^−16^ |
| AA | rs174591 | 11 | 61617676 | *FADS2* | T | A | 0.71832 | 0.017953589 | 0.001491899 | <1×10^−16^ |
| AA | rs174585 | 11 | 61611694 | *FADS2* | G | A | 0.78304 | 0.018018992 | 0.001511651 | <1×10^−16^ |
| AA | rs174448 | 11 | 61639573 |  | A | G | 0.64303 | 0.015302061 | 0.00129954 | <1×10^−16^ |
| AA | rs174589 | 11 | 61615803 | *FADS2* | C | G | 0.78765 | 0.017527017 | 0.001505997 | <1×10^−16^ |
| AA | rs2727270 | 11 | 61603237 | *FADS2* | C | T | 0.89029 | 0.022375137 | 0.001932447 | <1×10^−16^ |
| AA | rs2727271 | 11 | 61603358 | *FADS2* | A | T | 0.89 | 0.022344776 | 0.001933855 | <1×10^−16^ |
| AA | rs509360 | 11 | 61548559 | *C11orf9* | G | A | 0.649 | -0.018655647 | 0.001621963 | <1×10^−16^ |
| AA | rs2851682 | 11 | 61616012 | *FADS2* | A | G | 0.92129 | 0.028410035 | 0.002486314 | <1×10^−16^ |
| AA | rs2845573 | 11 | 61601908 | *FADS2* | A | G | 0.92668 | 0.026552235 | 0.002334587 | <1×10^−16^ |
| AA | rs174449 | 11 | 61640379 |  | A | G | 0.63271 | 0.01413323 | 0.001255778 | <1×10^−16^ |
| AA | rs2524299 | 11 | 61604782 | *FADS2* | A | T | 0.88064 | 0.021480109 | 0.001949754 | <1×10^−16^ |
| AA | rs174597 | 11 | 61621040 | *FADS2* | G | C | 0.74735 | 0.016773041 | 0.001538603 | <1×10^−16^ |
| AA | rs968567 | 11 | 61595564 | *FADS2* | C | T | 0.83527 | 0.019099505 | 0.001763269 | <1×10^−16^ |
| AA | rs422249 | 11 | 61639488 |  | C | T | 0.67098 | 0.014503632 | 0.001342686 | <1×10^−16^ |
| AA | rs2072114 | 11 | 61605215 | *FADS2* | A | G | 0.8814 | 0.020548561 | 0.00191428 | <1×10^−16^ |
| AA | rs2269928 | 11 | 61537529 | *C11orf9* | T | G | 0.77978 | 0.019499261 | 0.001818698 | <1×10^−16^ |
| AA | rs174532 | 11 | 61548874 | *C11orf9* | G | A | 0.72114 | -0.017638345 | 0.001693061 | <1×10^−16^ |
| AA | rs174611 | 11 | 61627881 | *FADS2* | T | C | 0.71039 | 0.014014928 | 0.00135256 | <1×10^−16^ |
| AA | rs174605 | 11 | 61626921 | *FADS2* | G | T | 0.71151 | 0.013918115 | 0.001347101 | <1×10^−16^ |
| AA | rs174455 | 11 | 61656117 | *FADS3* | G | A | 0.36538 | -0.012877611 | 0.001259959 | <1×10^−16^ |
| AA | rs2526678 | 11 | 61623793 | *FADS2* | G | A | 0.93057 | 0.028878715 | 0.002861908 | <1×10^−16^ |
| AA | rs412334 | 11 | 61560261 | *FEN1* | C | T | 0.85305 | -0.027620235 | 0.002751166 | <1×10^−16^ |
| AA | rs174616 | 11 | 61629122 | *FADS2* | G | A | 0.52756 | 0.012524792 | 0.001249924 | <1×10^−16^ |
| AA | rs174626 | 11 | 61637057 |  | A | G | 0.52886 | 0.012688919 | 0.001270477 | <1×10^−16^ |
| AA | rs174450 | 11 | 61641542 | *FADS3* | G | T | 0.47314 | -0.012731387 | 0.001293061 | <1×10^−16^ |
| AA | rs149803 | 11 | 61539020 | *C11orf9* | C | G | 0.74636 | -0.015835817 | 0.001793894 | <1×10^−16^ |
| AA | rs174627 | 11 | 61637466 |  | G | A | 0.835 | 0.015374786 | 0.001793824 | <1×10^−16^ |
| AA | rs174468 | 11 | 61663691 |  | G | A | 0.60365 | -0.012450023 | 0.001468168 | <1×10^−16^ |
| AA | rs666870 | 11 | 61677479 | *RAB3IL1* | G | A | 0.6013 | -0.0125676 | 0.001503774 | 1.10×10^−16^ |
| AA | rs174478 | 11 | 61678576 | *RAB3IL1* | T | G | 0.60099 | -0.012569856 | 0.001504329 | 1.10×10^−16^ |
| AA | rs174634 | 11 | 61647387 | *FADS3* | C | G | 0.72588 | 0.011703135 | 0.001401397 | 1.10×10^−16^ |
| AA | rs174479 | 11 | 61678754 | *RAB3IL1* | C | G | 0.81614 | 0.01555274 | 0.001871898 | 1.10×10^−16^ |
| AA | rs174464 | 11 | 61657926 | *FADS3* | G | A | 0.72982 | 0.011685088 | 0.001424754 | 2.20×10^−16^ |
| AA | rs174476 | 11 | 61674118 | *RAB3IL1* | C | T | 0.60208 | -0.012249595 | 0.001499231 | 3.30×10^−16^ |
| AA | rs1000778 | 11 | 61655305 | *FADS3* | G | A | 0.72904 | 0.011228085 | 0.001379677 | 4.40×10^−16^ |
| AA | rs174469 | 11 | 61667443 | *RAB3IL1* | C | T | 0.82555 | 0.014755401 | 0.001813425 | 4.40×10^−16^ |
| AA | rs174456 | 11 | 61656182 | *FADS3* | A | C | 0.72918 | 0.011202139 | 0.001378583 | 4.40×10^−16^ |
| AA | rs526126 | 11 | 61624885 | *FADS2* | C | G | 0.76593 | 0.016088208 | 0.00202466 | 1.90×10^−15^ |
| AA | rs174602 | 11 | 61624414 | *FADS2* | T | C | 0.76774 | 0.016117955 | 0.00204734 | 3.40×10^−15^ |
| AA | rs198462 | 11 | 61524119 | *C11orf9* | G | A | 0.48644 | -0.010170448 | 0.001297982 | 4.70×10^−15^ |
| AA | rs198476 | 11 | 61525730 | *C11orf9* | G | A | 0.47167 | -0.010063125 | 0.001334135 | 4.60×10^−14^ |
| AA | rs198464 | 11 | 61521621 |  | G | A | 0.48411 | -0.009390417 | 0.001253592 | 6.90×10^−14^ |
| AA | rs1692120 | 11 | 61417472 |  | G | A | 0.56219 | -0.011385056 | 0.001721542 | 3.80×10^−11^ |
| AA | rs650436 | 11 | 61536430 | *C11orf9* | C | T | 0.57937 | -0.009229438 | 0.00143508 | 1.30×10^−10^ |
| AA | rs2453710 | 11 | 61406542 | *RPLP0P2* | A | G | 0.58218 | -0.011508617 | 0.0018469 | 4.60×10^−10^ |
| DPA-n3 | rs8523 | 6 | 10981053 | *ELOVL2* | A | G | 0.44465 | 0.011293305 | 0.002041436 | 3.20×10^−8^ |
| DPA-n3 | rs3798707 | 6 | 10991935 | *ELOVL2* | T | C | 0.44596 | 0.011325177 | 0.002047072 | 3.20×10^−8^ |
| DPA-n3 | rs4532436 | 6 | 10983971 | *ELOVL2* | G | C | 0.44571 | 0.011295698 | 0.002047548 | 3.50×10^−8^ |
| DPA-n3 | rs4713103 | 6 | 10969141 | *SYCP2L* | T | G | 0.46298 | 0.01132793 | 0.002057244 | 3.70×10^−8^ |
| DPA-n3 | rs1225717 | 6 | 10978240 |  | G | A | 0.46545 | 0.011529213 | 0.002095265 | 3.70×10^−8^ |
| DPA-n3 | rs3798711 | 6 | 11002810 | *ELOVL2* | C | T | 0.44625 | 0.01120606 | 0.002047701 | 4.40×10^−8^ |
| DPA-n3 | rs2295602 | 6 | 11005842 | *ELOVL2* | C | T | 0.44643 | 0.011203922 | 0.002047984 | 4.50×10^−8^ |
| DPA-n3 | rs174549 | 11 | 61571382 | *FADS1* | G | A | 0.70775 | 0.01248346 | 0.002243944 | 2.60×10^−8^ |
| DPA-n3 | rs174555 | 11 | 61579760 | *FADS1* | T | C | 0.70764 | 0.012503236 | 0.002257835 | 3.10×10^−8^ |
| DPA-n3 | rs174556 | 11 | 61580635 | *FADS1* | C | T | 0.70764 | 0.01250357 | 0.002258417 | 3.10×10^−8^ |
| DTA | rs174546 | 11 | 61569830 | *FADS1* | C | T | 0.67116 | 0.028954763 | 0.003208542 | <1×10^−16^ |
| DTA | rs174545 | 11 | 61569306 | *FADS1* | C | G | 0.6711 | 0.028960669 | 0.003208942 | <1×10^−16^ |
| DTA | rs174547 | 11 | 61570783 | *FADS1* | T | C | 0.67136 | 0.02893559 | 0.003207219 | <1×10^−16^ |
| DTA | rs174550 | 11 | 61571478 | *FADS1* | T | C | 0.67143 | 0.028901207 | 0.00320778 | <1×10^−16^ |
| DTA | rs174537 | 11 | 61552680 | *C11orf9* | G | T | 0.66488 | 0.029222614 | 0.003242213 | <1×10^−16^ |
| DTA | rs174536 | 11 | 61551927 | *C11orf9* | A | C | 0.66386 | 0.029169235 | 0.00324112 | <1×10^−16^ |
| DTA | rs174535 | 11 | 61551356 | *C11orf9* | T | C | 0.65839 | 0.028930165 | 0.003242579 | <1×10^−16^ |
| DTA | rs1535 | 11 | 61597972 | *FADS2* | A | G | 0.66595 | 0.029022244 | 0.003272799 | <1×10^−16^ |
| DTA | rs174538 | 11 | 61560081 | *C11orf10* | G | A | 0.69487 | 0.028802552 | 0.003253572 | <1×10^−16^ |
| DTA | rs4246215 | 11 | 61564299 | *FEN1* | G | T | 0.65088 | 0.028385671 | 0.003248709 | <1×10^−16^ |
| DTA | rs174541 | 11 | 61565908 |  | T | C | 0.65148 | 0.028344796 | 0.003245402 | <1×10^−16^ |
| DTA | rs174576 | 11 | 61603510 | *FADS2* | C | A | 0.65841 | 0.027746738 | 0.003302934 | <1×10^−16^ |
| DTA | rs174574 | 11 | 61600342 | *FADS2* | C | A | 0.63796 | 0.028012059 | 0.00334974 | 1.10×10^−16^ |
| DTA | rs174555 | 11 | 61579760 | *FADS1* | T | C | 0.70764 | 0.027768362 | 0.003322717 | 1.10×10^−16^ |
| DTA | rs174556 | 11 | 61580635 | *FADS1* | C | T | 0.70764 | 0.027778084 | 0.003323559 | 1.10×10^−16^ |
| DTA | rs174549 | 11 | 61571382 | *FADS1* | G | A | 0.70775 | 0.027546529 | 0.003302683 | 1.10×10^−16^ |
| DTA | rs102275 | 11 | 61557803 | *C11orf10* | T | C | 0.62686 | 0.027917164 | 0.003355333 | 1.10×10^−16^ |
| DTA | rs174577 | 11 | 61604814 | *FADS2* | C | A | 0.6524 | 0.02741364 | 0.00330046 | 1.10×10^−16^ |
| DTA | rs174548 | 11 | 61571348 | *FADS1* | C | G | 0.69996 | 0.027024764 | 0.003288992 | 2.20×10^−16^ |
| DTA | rs174578 | 11 | 61605499 | *FADS2* | T | A | 0.65154 | 0.026865055 | 0.003296302 | 3.30×10^−16^ |
| DTA | rs174583 | 11 | 61609750 | *FADS2* | C | T | 0.65299 | 0.026853695 | 0.003314948 | 5.60×10^−16^ |
| DTA | rs174534 | 11 | 61549458 | *C11orf9* | A | G | 0.66316 | 0.0246155 | 0.003138156 | 4.30×10^−15^ |
| DTA | rs108499 | 11 | 61547237 | *C11orf9* | C | T | 0.67142 | 0.025336206 | 0.003231711 | 4.60×10^−15^ |
| DTA | rs174528 | 11 | 61543499 | *C11orf9* | T | C | 0.61669 | 0.025454057 | 0.003314775 | 1.60×10^−14^ |
| DTA | rs174601 | 11 | 61623140 | *FADS2* | C | T | 0.61373 | 0.025711832 | 0.003466068 | 1.20×10^−13^ |
| DTA | rs174579 | 11 | 61605613 | *FADS2* | C | T | 0.78402 | 0.024832406 | 0.003742543 | 3.20×10^−11^ |
| DTA | rs174585 | 11 | 61611694 | *FADS2* | G | A | 0.78304 | 0.024103702 | 0.003722047 | 9.40×10^−11^ |
| DTA | rs174589 | 11 | 61615803 | *FADS2* | C | G | 0.78765 | 0.023079185 | 0.003705559 | 4.70×10^−10^ |
| DTA | rs174575 | 11 | 61602003 | *FADS2* | C | G | 0.7332 | 0.022193588 | 0.00363031 | 9.80×10^−10^ |
| DTA | rs174570 | 11 | 61597212 | *FADS2* | C | T | 0.86422 | 0.028406585 | 0.004715405 | 1.70×10^−9^ |
| DTA | rs968567 | 11 | 61595564 | *FADS2* | C | T | 0.83527 | 0.025899899 | 0.004325846 | 2.10×10^−9^ |
| DTA | rs2269928 | 11 | 61537529 | *C11orf9* | T | G | 0.77978 | 0.026124322 | 0.004452842 | 4.40×10^−9^ |
| DTA | rs174593 | 11 | 61618831 | *FADS2* | T | C | 0.7208 | 0.020456431 | 0.003622509 | 1.60×10^−8^ |

PUFA, polyunsaturated fatty acid; ALA, α-linolenic acid; LA, linoleic acid; GLA, γ-linolenic acid; DGLA, dihomo-γ-linolenic acid; AA, arachidonic acid; DPA-n3, docosapentaenoic acid; DTA, docosatetraenoic acid; SNP, single nucleotide polymorphism; Chr, chromosome; EAF, Effect allele frequency; SE, standard error. For the 413 genome-wide significant associations (188 unique SNPs) reported by Tintle *et al.* 2015, a total of 369 associations (171 unique SNPs) remained significant at p < 5.0×10^−8^ in our repeating analysis with the Framingham Heart Study. Their summary statistics were reported in this table. Among them, genetic instruments were further selected based on different LD cutoffs.**Supplementary Table 6.** Forward Mendelian randomization estimates of associations of genetically predicted polyunsaturated fatty acids with COVID-19 severity based on the release 5 HGI A2.

|  |  |  | **IVW_MRE** | | | **MR-Egger** | | | **Wald ratio** | | | **MR-Egger intercept** | | **Heterogeneity test** | | **Weighted median** | | |  |  |
| --- | --- | --- | --- | --- | --- | --- | --- | --- | --- | --- | --- | --- | --- | --- | --- | --- | --- | --- | --- | --- |
| **Group name** | **PUFA** | **R^2^** | **β** | **SE** | ***P*** | **β** | **SE** | ***P*** | **β** | **SE** | ***P*** | **β** | ***P*** | ***P*_IVW** | ***P*_Egger** | **β** | **SE** | ***P*** | **nsnps** | ***F*-statistic** |
| Plasma | ALA | 0.001 | - | - | - | - | - | - | 0.107 | 0.089 | 0.23 | - | - | - | - | - | - | - | 1 | 256 |
| Plasma | ALA | 0.01 | - | - | - | - | - | - | 0.108 | 0.09 | 0.23 | - | - | - | - | - | - | - | 1 | 312 |
| Plasma | ALA | 0.1 | 0.099 | 0.027 | 2.00E-04 | 0.042 | 0.17 | 0.828 | - | - | - | 0.016 | 0.745 | 0.935 | 0.866 | 0.108 | 0.078 | 0.164 | 4 | 436 |
| Plasma | ALA | 0.3 | 0.125 | 0.046 | 0.006 | -0.075 | 0.132 | 0.58 | - | - | - | 0.05 | 0.134 | 0.441 | 0.582 | 0.103 | 0.063 | 0.104 | 13 | 1107 |
| RBC | ALA | 0.001 | - | - | - | - | - | - | 0.048 | 0.172 | 0.78 | - | - | - | - | - | - | - | 1 | 31 |
| RBC | ALA | 0.01 | - | - | - | - | - | - | 0.048 | 0.172 | 0.78 | - | - | - | - | - | - | - | 1 | 31 |
| RBC | ALA | 0.1 | - | - | - | - | - | - | 0.048 | 0.172 | 0.78 | - | - | - | - | - | - | - | 1 | 31 |
| RBC | ALA | 0.3 | - | - | - | - | - | - | 0.048 | 0.172 | 0.78 | - | - | - | - | - | - | - | 1 | 31 |
| Plasma | LA | 0.001 | 0.073 | 0.038 | 0.053 | 0.013 | 0.081 | 0.899 | - | - | - | 0.027 | 0.517 | 0.574 | 0.645 | 0.065 | 0.051 | 0.204 | 3 | 1329 |
| RBC | LA | 0.001 | - | - | - | - | - | - | 0.04 | 0.079 | 0.611 | - | - | - | - | - | - | - | 1 | 133 |
| RBC | LA | 0.01 | - | - | - | - | - | - | 0.04 | 0.079 | 0.611 | - | - | - | - | - | - | - | 1 | 133 |
| RBC | LA | 0.1 | 0.057 | 0.03 | 0.054 | - | - | - | - | - | - | - | - | - | - | - | - | - | 2 | 175 |
| RBC | LA | 0.3 | 0.072 | 0.037 | 0.053 | -0.099 | 0.2 | 0.635 | - | - | - | 0.05 | 0.414 | 0.534 | 0.509 | 0.042 | 0.051 | 0.406 | 9 | 566 |
| Plasma | GLA | 0.001 | -0.11 | 0.066 | 0.094 | - | - | - | - | - | - | - | - | - | - | - | - | - | 2 | 293 |
| RBC | GLA | 0.001 | - | - | - | - | - | - | -0.038 | 0.138 | 0.78 | - | - | - | - | - | - | - | 1 | 49 |
| RBC | GLA | 0.01 | - | - | - | - | - | - | -0.038 | 0.138 | 0.78 | - | - | - | - | - | - | - | 1 | 49 |
| RBC | GLA | 0.1 | - | - | - | - | - | - | -0.038 | 0.138 | 0.78 | - | - | - | - | - | - | - | 1 | 49 |
| RBC | GLA | 0.3 | - | - | - | - | - | - | -0.038 | 0.138 | 0.78 | - | - | - | - | - | - | - | 1 | 49 |
| Plasma | DGLA | 0.001 | 0.069 | 0.011 | 7.00E-11 | - | - | - | - | - | - | - | - | - | - | - | - | - | 2 | 1417 |
| RBC | DGLA | 0.001 | 0.017 | 0.103 | 0.87 | - | - | - | - | - | - | - | - | - | - | - | - | - | 2 | 542 |
| RBC | DGLA | 0.01 | 0.044 | 0.056 | 0.436 | -0.103 | 0.125 | 0.498 | - | - | - | 0.085 | 0.329 | 0.042 | 0.104 | 0.059 | 0.047 | 0.201 | 4 | 788 |
| RBC | DGLA | 0.1 | 0.045 | 0.019 | 0.017 | 0.015 | 0.045 | 0.747 | - | - | - | 0.02 | 0.464 | 0.488 | 0.453 | 0.01 | 0.026 | 0.708 | 14 | 2375 |
| RBC | DGLA | 0.3 | 0.041 | 0.012 | 0.001 | -3.00E-04 | 0.032 | 0.992 | - | - | - | 0.027 | 0.168 | 0.672 | 0.733 | 0.016 | 0.019 | 0.408 | 26 | 5970 |
| Plasma | AA | 0.001 | -0.039 | 0.014 | 0.007 | - | - | - | - | - | - | - | - | - | - | - | - | - | 2 | 4616 |
| RBC | AA | 0.001 | -0.029 | 0.015 | 0.048 | -0.089 | 0.182 | 0.712 | - | - | - | 0.024 | 0.788 | 0.938 | 0.927 | -0.035 | 0.066 | 0.599 | 3 | 268 |
| RBC | AA | 0.01 | -0.041 | 0.021 | 0.049 | -0.114 | 0.166 | 0.616 | - | - | - | 0.031 | 0.72 | 0.872 | 0.816 | -0.044 | 0.065 | 0.493 | 3 | 287 |
| RBC | AA | 0.1 | -0.111 | 0.038 | 0.003 | -0.038 | 0.129 | 0.782 | - | - | - | -0.029 | 0.575 | 0.429 | 0.352 | -0.081 | 0.05 | 0.109 | 7 | 584 |
| RBC | AA | 0.3 | -0.078 | 0.024 | 0.001 | -0.015 | 0.076 | 0.842 | - | - | - | -0.026 | 0.4 | 0.345 | 0.33 | -0.051 | 0.033 | 0.118 | 14 | 1440 |
| Plasma | DPA-n3 | 0.001 | -0.114 | 0.051 | 0.026 | -0.066 | 0.157 | 0.746 | - | - | - | -0.016 | 0.792 | 0.426 | 0.216 | -0.113 | 0.058 | 0.051 | 3 | 835 |
| Plasma | DPA-n3 | 0.01 | -0.115 | 0.051 | 0.024 | -0.069 | 0.157 | 0.736 | - | - | - | -0.016 | 0.801 | 0.434 | 0.219 | -0.114 | 0.058 | 0.05 | 3 | 937 |
| Plasma | DPA-n3 | 0.1 | -0.116 | 0.027 | 2.00E-05 | -0.047 | 0.082 | 0.578 | - | - | - | -0.021 | 0.358 | 0.954 | 0.963 | -0.087 | 0.054 | 0.108 | 14 | 1506 |
| Plasma | DPA-n3 | 0.3 | -0.119 | 0.025 | 1.00E-06 | -0.02 | 0.062 | 0.744 | - | - | - | -0.03 | 0.084 | 0.77 | 0.869 | -0.084 | 0.041 | 0.039 | 29 | 3466 |
| RBC | DPA-n3 | 0.001 | -0.237 | 0.116 | 0.041 | - | - | - | - | - | - | - | - | - | - | - | - | - | 2 | 62 |
| RBC | DPA-n3 | 0.01 | -0.237 | 0.116 | 0.041 | - | - | - | - | - | - | - | - | - | - | - | - | - | 2 | 62 |
| RBC | DPA-n3 | 0.1 | -0.237 | 0.116 | 0.041 | - | - | - | - | - | - | - | - | - | - | - | - | - | 2 | 62 |
| RBC | DPA-n3 | 0.3 | -0.237 | 0.116 | 0.041 | - | - | - | - | - | - | - | - | - | - | - | - | - | 2 | 62 |
| RBC | DTA | 0.001 | - | - | - | - | - | - | -0.091 | 0.104 | 0.382 | - | - | - | - | - | - | - | 1 | 80 |
| RBC | DTA | 0.01 | - | - | - | - | - | - | -0.091 | 0.104 | 0.382 | - | - | - | - | - | - | - | 1 | 80 |
| RBC | DTA | 0.1 | - | - | - | - | - | - | -0.091 | 0.104 | 0.382 | - | - | - | - | - | - | - | 1 | 80 |
| RBC | DTA | 0.3 | -0.081 | 0.016 | 7.00E-07 | - | - | - | - | - | - | - | - | - | - | - | - | - | 2 | 114 |
| Plasma | DHA | 0.001 | - | - | - | - | - | - | 0.47 | 0.212 | 0.026 | - | - | - | - | - | - | - | 1 | 65 |
| Plasma | DHA | 0.01 | - | - | - | - | - | - | 0.469 | 0.211 | 0.026 | - | - | - | - | - | - | - | 1 | 64 |
| Plasma | DHA | 0.1 | - | - | - | - | - | - | 0.469 | 0.211 | 0.026 | - | - | - | - | - | - | - | 1 | 64 |
| Plasma | DHA | 0.3 | 0.393 | 0.137 | 0.004 | - | - | - | - | - | - | - | - | - | - | - | - | - | 2 | 95 |

PUFA, polyunsaturated fatty acid; RBC, red blood cell; ALA, α-linolenic acid; LA, linoleic acid; GLA, γ-linolenic acid; DGLA, dihomo-γ-linolenic acid; AA, arachidonic acid; DPA-n3, docosapentaenoic acid; DTA, docosatetraenoic acid; DHA, docosahexaenoic acid; β, causal effect size; SE, standard error; IVW_MRE, inverse-variance weighted random-effects model; Egger, MR-Egger; nsnps, number of SNPs retained for this analysis.

**Supplementary Table 7.** Forward Mendelian randomization estimates of associations of genetically predicted polyunsaturated fatty acids with COVID-19 severity based on the release 5 HGI B2.

|  |  |  | **IVW_MRE** | | | **MR-Egger** | | | **Wald ratio** | | | **MR-Egger intercept** | | **Heterogeneity test** | | **Weighted median** | | |  |  |
| --- | --- | --- | --- | --- | --- | --- | --- | --- | --- | --- | --- | --- | --- | --- | --- | --- | --- | --- | --- | --- |
| **Group name** | **PUFA** | **R^2^** | **β** | **SE** | ***P*** | **β** | **SE** | ***P*** | **β** | **SE** | ***P*** | **β** | ***P*** | ***P*_IVW** | ***P*_Egger** | **β** | **SE** | ***P*** | **nsnps** | ***F*-statistic** |
| Plasma | ALA | 0.001 | - | - | - | - | - | - | 0.101 | 0.061 | 0.098 | - | - | - | - | - | - | - | 1 | 256 |
| Plasma | ALA | 0.01 | - | - | - | - | - | - | 0.101 | 0.061 | 0.098 | - | - | - | - | - | - | - | 1 | 312 |
| Plasma | ALA | 0.1 | 0.109 | 0.083 | 0.192 | -0.090 | 0.195 | 0.689 | - | - | - | 0.057 | 0.380 | 0.020 | 0.049 | 0.118 | 0.056 | 0.034 | 4 | 436 |
| Plasma | ALA | 0.3 | 0.101 | 0.038 | 0.008 | -0.108 | 0.096 | 0.284 | - | - | - | 0.052 | 0.041 | 0.042 | 0.203 | 0.094 | 0.039 | 0.016 | 13 | 1107 |
| RBC | ALA | 0.001 | - | - | - | - | - | - | 0.122 | 0.116 | 0.291 | - | - | - | - | - | - | - | 1 | 31 |
| RBC | ALA | 0.01 | - | - | - | - | - | - | 0.122 | 0.116 | 0.291 | - | - | - | - | - | - | - | 1 | 31 |
| RBC | ALA | 0.1 | - | - | - | - | - | - | 0.122 | 0.116 | 0.291 | - | - | - | - | - | - | - | 1 | 31 |
| RBC | ALA | 0.3 | - | - | - | - | - | - | 0.122 | 0.116 | 0.291 | - | - | - | - | - | - | - | 1 | 31 |
| Plasma | LA | 0.001 | 0.058 | 0.024 | 0.015 | 0.052 | 0.055 | 0.517 | - | - | - | 0.003 | 0.915 | 0.617 | 0.331 | 0.057 | 0.036 | 0.108 | 3 | 1329 |
| RBC | LA | 0.001 | - | - | - | - | - | - | 0.082 | 0.053 | 0.126 | - | - | - | - | - | - | - | 1 | 133 |
| RBC | LA | 0.01 | - | - | - | - | - | - | 0.082 | 0.053 | 0.126 | - | - | - | - | - | - | - | 1 | 133 |
| RBC | LA | 0.1 | 0.109 | 0.048 | 0.024 | - | - | - | - | - | - | - | - | - | - | - | - | - | 2 | 175 |
| RBC | LA | 0.3 | 0.076 | 0.023 | 0.001 | -0.045 | 0.136 | 0.752 | - | - | - | 0.036 | 0.397 | 0.622 | 0.610 | 0.074 | 0.034 | 0.030 | 9 | 566 |
| Plasma | GLA | 0.001 | -0.117 | 0.013 | 6.60e-20 | - | - | - | - | - | - | - | - | - | - | - | - | - | 2 | 293 |
| RBC | GLA | 0.001 | - | - | - | - | - | - | -0.098 | 0.093 | 0.291 | - | - | - | - | - | - | - | 1 | 49 |
| RBC | GLA | 0.01 | - | - | - | - | - | - | -0.098 | 0.093 | 0.291 | - | - | - | - | - | - | - | 1 | 49 |
| RBC | GLA | 0.1 | - | - | - | - | - | - | -0.098 | 0.093 | 0.291 | - | - | - | - | - | - | - | 1 | 49 |
| RBC | GLA | 0.3 | - | - | - | - | - | - | -0.098 | 0.093 | 0.291 | - | - | - | - | - | - | - | 1 | 49 |
| Plasma | DGLA | 0.001 | 0.048 | 0.040 | 0.231 | - | - | - | - | - | - | - | - | - | - | - | - | - | 2 | 1417 |
| RBC | DGLA | 0.001 | 0.010 | 0.044 | 0.828 | - | - | - | - | - | - | - | - | - | - | - | - | - | 2 | 542 |
| RBC | DGLA | 0.01 | 0.006 | 0.022 | 0.774 | -0.069 | 0.054 | 0.328 | - | - | - | 0.044 | 0.266 | 0.332 | 0.582 | -0.007 | 0.024 | 0.760 | 4 | 788 |
| RBC | DGLA | 0.1 | 0.029 | 0.017 | 0.080 | 0.039 | 0.040 | 0.353 | - | - | - | -0.006 | 0.795 | 0.091 | 0.065 | 0.023 | 0.017 | 0.177 | 13 | 2341 |
| RBC | DGLA | 0.3 | 0.033 | 0.010 | 0.001 | 0.006 | 0.024 | 0.819 | - | - | - | 0.017 | 0.238 | 0.089 | 0.106 | 0.026 | 0.011 | 0.021 | 25 | 5936 |
| Plasma | AA | 0.001 | -0.038 | 0.004 | 3.23e-20 | - | - | - | - | - | - | - | - | - | - | - | - | - | 2 | 4616 |
| RBC | AA | 0.001 | -0.049 | 0.011 | 1.32e-05 | -0.097 | 0.123 | 0.574 | - | - | - | 0.019 | 0.753 | 0.912 | 0.894 | -0.053 | 0.041 | 0.189 | 3 | 268 |
| RBC | AA | 0.01 | -0.056 | 0.015 | 1.78e-04 | -0.112 | 0.113 | 0.502 | - | - | - | 0.024 | 0.694 | 0.844 | 0.795 | -0.059 | 0.039 | 0.135 | 3 | 287 |
| RBC | AA | 0.1 | -0.069 | 0.015 | 3.89e-06 | -0.056 | 0.082 | 0.528 | - | - | - | -0.005 | 0.871 | 0.888 | 0.807 | -0.059 | 0.030 | 0.047 | 7 | 584 |
| RBC | AA | 0.3 | -0.055 | 0.012 | 9.17e-06 | -0.039 | 0.048 | 0.425 | - | - | - | -0.006 | 0.736 | 0.791 | 0.734 | -0.052 | 0.021 | 0.012 | 14 | 1440 |
| Plasma | DPA-n3 | 0.001 | -0.069 | 0.007 | 4.73e-20 | -0.089 | 0.082 | 0.475 | - | - | - | 0.007 | 0.827 | 0.962 | 0.977 | -0.070 | 0.038 | 0.067 | 3 | 835 |
| Plasma | DPA-n3 | 0.01 | -0.069 | 0.008 | 6.21e-20 | -0.089 | 0.083 | 0.475 | - | - | - | 0.007 | 0.826 | 0.961 | 0.990 | -0.070 | 0.039 | 0.073 | 3 | 937 |
| Plasma | DPA-n3 | 0.1 | -0.076 | 0.028 | 0.007 | 0.012 | 0.053 | 0.830 | - | - | - | -0.027 | 0.081 | 0.302 | 0.491 | -0.070 | 0.034 | 0.042 | 14 | 1506 |
| Plasma | DPA-n3 | 0.3 | -0.072 | 0.019 | 1.65e-04 | 0.015 | 0.040 | 0.710 | - | - | - | -0.026 | 0.022 | 0.188 | 0.401 | -0.065 | 0.026 | 0.012 | 29 | 3466 |
| RBC | DPA-n3 | 0.001 | -0.130 | 0.033 | 9.30e-05 | - | - | - | - | - | - | - | - | - | - | - | - | - | 2 | 62 |
| RBC | DPA-n3 | 0.01 | -0.130 | 0.033 | 9.30e-05 | - | - | - | - | - | - | - | - | - | - | - | - | - | 2 | 62 |
| RBC | DPA-n3 | 0.1 | -0.130 | 0.033 | 9.30e-05 | - | - | - | - | - | - | - | - | - | - | - | - | - | 2 | 62 |
| RBC | DPA-n3 | 0.3 | -0.130 | 0.033 | 9.30e-05 | - | - | - | - | - | - | - | - | - | - | - | - | - | 2 | 62 |
| RBC | DTA | 0.001 | - | - | - | - | - | - | -0.102 | 0.071 | 0.148 | - | - | - | - | - | - | - | 1 | 80 |
| RBC | DTA | 0.01 | - | - | - | - | - | - | -0.102 | 0.071 | 0.148 | - | - | - | - | - | - | - | 1 | 80 |
| RBC | DTA | 0.1 | - | - | - | - | - | - | -0.102 | 0.071 | 0.148 | - | - | - | - | - | - | - | 1 | 80 |
| RBC | DTA | 0.3 | -0.098 | 0.006 | 3.04e-60 | - | - | - | - | - | - | - | - | - | - | - | - | - | 2 | 114 |
| Plasma | DHA | 0.001 | - | - | - | - | - | - | 0.161 | 0.146 | 0.269 | - | - | - | - | - | - | - | 1 | 65 |
| Plasma | DHA | 0.01 | - | - | - | - | - | - | 0.161 | 0.146 | 0.269 | - | - | - | - | - | - | - | 1 | 64 |
| Plasma | DHA | 0.1 | - | - | - | - | - | - | 0.161 | 0.146 | 0.269 | - | - | - | - | - | - | - | 1 | 64 |
| Plasma | DHA | 0.3 | 0.170 | 0.014 | 5.32e-34 | - | - | - | - | - | - | - | - | - | - | - | - | - | 2 | 95 |

PUFA, polyunsaturated fatty acid; RBC, red blood cell; ALA, α-linolenic acid; LA, linoleic acid; GLA, γ-linolenic acid; DGLA, dihomo-γ-linolenic acid; AA, arachidonic acid; DPA-n3, docosapentaenoic acid; DTA, docosatetraenoic acid; DHA, docosahexaenoic acid; β, causal effect size; SE, standard error; IVW_MRE, inverse-variance weighted random-effects model; Egger, MR-Egger; nsnps, number of SNPs retained for this analysis.

**Supplementary Table 8.** Forward Mendelian randomization estimates of associations of genetically predicted polyunsaturated fatty acids with COVID-19 severity based on the release 5 HGI B1.

|  |  |  | **IVW_MRE** | | | **MR-Egger** | | | **Wald ratio** | | | **MR-Egger intercept** | | **Heterogeneity test** | | **Weighted median** | | |  |  |
| --- | --- | --- | --- | --- | --- | --- | --- | --- | --- | --- | --- | --- | --- | --- | --- | --- | --- | --- | --- | --- |
| **Group name** | **PUFA** | **R^2^** | **β** | **SE** | ***P*** | **β** | **SE** | ***P*** | **β** | **SE** | ***P*** | **β** | ***P*** | ***P*_IVW** | ***P*_Egger** | **β** | **SE** | ***P*** | **nsnps** | ***F*-statistic** |
| Plasma | ALA | 0.001 | - | - | - | - | - | - | 0.211 | 0.102 | 0.038 | - | - | - | - | - | - | - | 1 | 256 |
| Plasma | ALA | 0.01 | - | - | - | - | - | - | 0.213 | 0.102 | 0.038 | - | - | - | - | - | - | - | 1 | 312 |
| Plasma | ALA | 0.1 | 0.224 | 0.179 | 0.211 | -0.174 | 0.45 | 0.737 | - | - | - | 0.115 | 0.437 | 0.001 | 0.002 | 0.229 | 0.097 | 0.018 | 4 | 436 |
| Plasma | ALA | 0.3 | 0.183 | 0.07 | 0.009 | -0.053 | 0.204 | 0.8 | - | - | - | 0.058 | 0.245 | 0.004 | 0.007 | 0.209 | 0.065 | 0.001 | 13 | 1107 |
| RBC | ALA | 0.001 | - | - | - | - | - | - | 0.362 | 0.193 | 0.061 | - | - | - | - | - | - | - | 1 | 31 |
| RBC | ALA | 0.01 | - | - | - | - | - | - | 0.362 | 0.193 | 0.061 | - | - | - | - | - | - | - | 1 | 31 |
| RBC | ALA | 0.1 | - | - | - | - | - | - | 0.362 | 0.193 | 0.061 | - | - | - | - | - | - | - | 1 | 31 |
| RBC | ALA | 0.3 | - | - | - | - | - | - | 0.362 | 0.193 | 0.061 | - | - | - | - | - | - | - | 1 | 31 |
| Plasma | LA | 0.001 | 0.112 | 0.071 | 0.117 | 0.138 | 0.159 | 0.545 | - | - | - | -0.011 | 0.868 | 0.213 | 0.085 | 0.113 | 0.058 | 0.05 | 3 | 1329 |
| RBC | LA | 0.001 | - | - | - | - | - | - | 0.227 | 0.09 | 0.011 | - | - | - | - | - | - | - | 1 | 133 |
| RBC | LA | 0.01 | - | - | - | - | - | - | 0.227 | 0.09 | 0.011 | - | - | - | - | - | - | - | 1 | 133 |
| RBC | LA | 0.1 | 0.27 | 0.076 | 3.90E-04 | - | - | - | - | - | - | - | - | - | - | - | - | - | 2 | 175 |
| RBC | LA | 0.3 | 0.183 | 0.038 | 1.75E-06 | 0.057 | 0.228 | 0.81 | - | - | - | 0.037 | 0.592 | 0.55 | 0.476 | 0.216 | 0.056 | 1.04E-04 | 9 | 566 |
| Plasma | GLA | 0.001 | -0.265 | 0.031 | 2.82E-17 | - | - | - | - | - | - | - | - | - | - | - | - | - | 2 | 293 |
| RBC | GLA | 0.001 | - | - | - | - | - | - | -0.29 | 0.155 | 0.061 | - | - | - | - | - | - | - | 1 | 49 |
| RBC | GLA | 0.01 | - | - | - | - | - | - | -0.29 | 0.155 | 0.061 | - | - | - | - | - | - | - | 1 | 49 |
| RBC | GLA | 0.1 | - | - | - | - | - | - | -0.29 | 0.155 | 0.061 | - | - | - | - | - | - | - | 1 | 49 |
| RBC | GLA | 0.3 | - | - | - | - | - | - | -0.29 | 0.155 | 0.061 | - | - | - | - | - | - | - | 1 | 49 |
| Plasma | DGLA | 0.001 | 0.074 | 0.118 | 0.528 | - | - | - | - | - | - | - | - | - | - | - | - | - | 2 | 1417 |
| RBC | DGLA | 0.001 | 0.005 | 0.025 | 0.84 | - | - | - | - | - | - | - | - | - | - | - | - | - | 2 | 542 |
| RBC | DGLA | 0.01 | -0.015 | 0.027 | 0.591 | -0.014 | 0.086 | 0.886 | - | - | - | -4.98E-04 | 0.993 | 0.55 | 0.348 | -0.006 | 0.036 | 0.876 | 4 | 788 |
| RBC | DGLA | 0.1 | 0.048 | 0.029 | 0.101 | 0.087 | 0.07 | 0.24 | - | - | - | -0.025 | 0.551 | 0.029 | 0.024 | 0.053 | 0.029 | 0.069 | 13 | 2341 |
| RBC | DGLA | 0.3 | 0.059 | 0.017 | 0.001 | 0.049 | 0.044 | 0.279 | - | - | - | 0.007 | 0.804 | 0.02 | 0.014 | 0.054 | 0.02 | 0.007 | 25 | 5936 |
| Plasma | AA | 0.001 | -0.082 | 0.031 | 0.008 | - | - | - | - | - | - | - | - | - | - | - | - | - | 2 | 4616 |
| RBC | AA | 0.001 | -0.171 | 0.007 | 8.57E-130 | -0.139 | 0.204 | 0.621 | - | - | - | -0.013 | 0.895 | 0.986 | 0.998 | -0.167 | 0.067 | 0.013 | 3 | 268 |
| RBC | AA | 0.01 | -0.167 | 0.009 | 4.60E-80 | -0.13 | 0.188 | 0.615 | - | - | - | -0.016 | 0.871 | 0.978 | 0.956 | -0.164 | 0.066 | 0.012 | 3 | 287 |
| RBC | AA | 0.1 | -0.109 | 0.031 | 4.43E-04 | -0.185 | 0.137 | 0.234 | - | - | - | 0.03 | 0.587 | 0.687 | 0.61 | -0.113 | 0.05 | 0.025 | 7 | 584 |
| RBC | AA | 0.3 | -0.1 | 0.025 | 5.99E-05 | -0.137 | 0.083 | 0.126 | - | - | - | 0.015 | 0.652 | 0.418 | 0.358 | -0.114 | 0.032 | 3.62E-04 | 14 | 1440 |
| Plasma | DPA-n3 | 0.001 | -0.131 | 0.028 | 3.59E-06 | -0.164 | 0.132 | 0.431 | - | - | - | 0.011 | 0.82 | 0.818 | 0.573 | -0.149 | 0.064 | 0.021 | 3 | 835 |
| Plasma | DPA-n3 | 0.01 | -0.131 | 0.029 | 6.00E-06 | -0.164 | 0.132 | 0.433 | - | - | - | 0.011 | 0.824 | 0.811 | 0.561 | -0.15 | 0.067 | 0.025 | 3 | 937 |
| Plasma | DPA-n3 | 0.1 | -0.138 | 0.058 | 0.018 | -0.017 | 0.115 | 0.887 | - | - | - | -0.038 | 0.244 | 0.02 | 0.031 | -0.153 | 0.064 | 0.017 | 14 | 1506 |
| Plasma | DPA-n3 | 0.3 | -0.121 | 0.034 | 3.48E-04 | -0.028 | 0.075 | 0.718 | - | - | - | -0.028 | 0.178 | 0.034 | 0.048 | -0.15 | 0.041 | 2.88E-04 | 29 | 3466 |
| RBC | DPA-n3 | 0.001 | -0.274 | 0.131 | 0.036 | - | - | - | - | - | - | - | - | - | - | - | - | - | 2 | 62 |
| RBC | DPA-n3 | 0.01 | -0.274 | 0.131 | 0.036 | - | - | - | - | - | - | - | - | - | - | - | - | - | 2 | 62 |
| RBC | DPA-n3 | 0.1 | -0.274 | 0.131 | 0.036 | - | - | - | - | - | - | - | - | - | - | - | - | - | 2 | 62 |
| RBC | DPA-n3 | 0.3 | -0.274 | 0.131 | 0.036 | - | - | - | - | - | - | - | - | - | - | - | - | - | 2 | 62 |
| RBC | DTA | 0.001 | - | - | - | - | - | - | -0.25 | 0.118 | 0.034 | - | - | - | - | - | - | - | 1 | 80 |
| RBC | DTA | 0.01 | - | - | - | - | - | - | -0.25 | 0.118 | 0.034 | - | - | - | - | - | - | - | 1 | 80 |
| RBC | DTA | 0.1 | - | - | - | - | - | - | -0.25 | 0.118 | 0.034 | - | - | - | - | - | - | - | 1 | 80 |
| RBC | DTA | 0.3 | -0.271 | 0.027 | 2.21E-23 | - | - | - | - | - | - | - | - | - | - | - | - | - | 2 | 114 |
| Plasma | DHA | 0.001 | - | - | - | - | - | - | 0.103 | 0.246 | 0.674 | - | - | - | - | - | - | - | 1 | 65 |
| Plasma | DHA | 0.01 | - | - | - | - | - | - | 0.103 | 0.245 | 0.674 | - | - | - | - | - | - | - | 1 | 64 |
| Plasma | DHA | 0.1 | - | - | - | - | - | - | 0.103 | 0.245 | 0.674 | - | - | - | - | - | - | - | 1 | 64 |
| Plasma | DHA | 0.3 | -0.005 | 0.158 | 0.974 | - | - | - | - | - | - | - | - | - | - | - | - | - | 2 | 95 |

PUFA, polyunsaturated fatty acid; RBC, red blood cell; ALA, α-linolenic acid; LA, linoleic acid; GLA, γ-linolenic acid; DGLA, dihomo-γ-linolenic acid; AA, arachidonic acid; DPA-n3, docosapentaenoic acid; DTA, docosatetraenoic acid; DHA, docosahexaenoic acid; β, causal effect size; SE, standard error; IVW_MRE, inverse-variance weighted random-effects model; Egger, MR-Egger; nsnps, number of SNPs retained for this analysis.

**Supplementary Table 9.** Forward Mendelian randomization estimates of associations of genetically predicted polyunsaturated fatty acids with COVID-19 susceptibility based on the release 5 HGI C2.

|  |  |  | **IVW_MRE** | | | **MR-Egger** | | | **Wald ratio** | | | **MR-Egger intercept** | | **Heterogeneity test** | | **Weighted median** | | |  |  |
| --- | --- | --- | --- | --- | --- | --- | --- | --- | --- | --- | --- | --- | --- | --- | --- | --- | --- | --- | --- | --- |
| **Group name** | **PUFA** | **R^2^** | **β** | **SE** | ***P*** | **β** | **SE** | ***P*** | **β** | **SE** | ***P*** | **β** | ***P*** | ***P*_IVW** | ***P*_Egger** | **β** | **SE** | ***P*** | **nsnps** | ***F*-statistic** |
| Plasma | ALA | 0.001 | - | - | - | - | - | - | 0.012 | 0.031 | 0.697 | - | - | - | - | - | - | - | 1 | 256 |
| Plasma | ALA | 0.01 | - | - | - | - | - | - | 0.012 | 0.031 | 0.697 | - | - | - | - | - | - | - | 1 | 312 |
| Plasma | ALA | 0.1 | 0.010 | 0.008 | 0.211 | 0.031 | 0.056 | 0.636 | - | - | - | -0.006 | 0.728 | 0.938 | 0.882 | 0.010 | 0.025 | 0.693 | 4 | 436 |
| Plasma | ALA | 0.3 | 0.012 | 0.009 | 0.200 | -0.012 | 0.041 | 0.770 | - | - | - | 0.006 | 0.545 | 0.943 | 0.930 | 0.012 | 0.017 | 0.477 | 13 | 1107 |
| RBC | ALA | 0.001 | - | - | - | - | - | - | -4.57e-04 | 0.059 | 0.994 | - | - | - | - | - | - | - | 1 | 31 |
| RBC | ALA | 0.01 | - | - | - | - | - | - | -4.57e-04 | 0.059 | 0.994 | - | - | - | - | - | - | - | 1 | 31 |
| RBC | ALA | 0.1 | - | - | - | - | - | - | -4.57e-04 | 0.059 | 0.994 | - | - | - | - | - | - | - | 1 | 31 |
| RBC | ALA | 0.3 | - | - | - | - | - | - | -4.57e-04 | 0.059 | 0.994 | - | - | - | - | - | - | - | 1 | 31 |
| Plasma | LA | 0.001 | 0.007 | 0.009 | 0.430 | 0.010 | 0.028 | 0.779 | - | - | - | -0.001 | 0.914 | 0.761 | 0.468 | 0.008 | 0.018 | 0.669 | 3 | 1329 |
| RBC | LA | 0.001 | - | - | - | - | - | - | 2.25e-04 | 0.028 | 0.993 | - | - | - | - | - | - | - | 1 | 133 |
| RBC | LA | 0.01 | - | - | - | - | - | - | 2.25e-04 | 0.028 | 0.993 | - | - | - | - | - | - | - | 1 | 133 |
| RBC | LA | 0.1 | -0.004 | 0.008 | 0.594 | - | - | - | - | - | - | - | - | - | - | - | - | - | 2 | 175 |
| RBC | LA | 0.3 | -0.005 | 0.005 | 0.245 | 0.012 | 0.070 | 0.872 | - | - | - | -0.005 | 0.812 | 0.998 | 0.996 | -0.009 | 0.017 | 0.593 | 9 | 566 |
| Plasma | GLA | 0.001 | -0.006 | 0.023 | 0.775 | - | - | - | - | - | - | - | - | - | - | - | - | - | 2 | 293 |
| RBC | GLA | 0.001 | - | - | - | - | - | - | 3.67e-04 | 0.047 | 0.994 | - | - | - | - | - | - | - | 1 | 49 |
| RBC | GLA | 0.01 | - | - | - | - | - | - | 3.67e-04 | 0.047 | 0.994 | - | - | - | - | - | - | - | 1 | 49 |
| RBC | GLA | 0.1 | - | - | - | - | - | - | 3.67e-04 | 0.047 | 0.994 | - | - | - | - | - | - | - | 1 | 49 |
| RBC | GLA | 0.3 | - | - | - | - | - | - | 3.67e-04 | 0.047 | 0.994 | - | - | - | - | - | - | - | 1 | 49 |
| Plasma | DGLA | 0.001 | 0.011 | 0.005 | 0.031 | - | - | - | - | - | - | - | - | - | - | - | - | - | 2 | 1417 |
| RBC | DGLA | 0.001 | 0.010 | 0.004 | 0.007 | - | - | - | - | - | - | - | - | - | - | - | - | - | 2 | 542 |
| RBC | DGLA | 0.01 | 0.007 | 0.006 | 0.284 | 0.017 | 0.026 | 0.579 | - | - | - | -0.006 | 0.702 | 0.786 | 0.647 | 0.009 | 0.012 | 0.438 | 4 | 788 |
| RBC | DGLA | 0.1 | 0.001 | 0.005 | 0.793 | 0.001 | 0.015 | 0.943 | - | - | - | 1.23e-04 | 0.989 | 0.850 | 0.792 | -0.002 | 0.008 | 0.767 | 14 | 2375 |
| RBC | DGLA | 0.3 | 3.29e-04 | 0.003 | 0.896 | 0.002 | 0.010 | 0.854 | - | - | - | -0.001 | 0.869 | 0.997 | 0.996 | -0.002 | 0.005 | 0.702 | 26 | 5970 |
| Plasma | AA | 0.001 | -0.004 | 0.007 | 0.576 | - | - | - | - | - | - | - | - | - | - | - | - | - | 2 | 4616 |
| RBC | AA | 0.001 | 0.016 | 0.008 | 0.050 | -0.021 | 0.063 | 0.793 | - | - | - | 0.015 | 0.648 | 0.821 | 0.914 | 0.013 | 0.020 | 0.527 | 3 | 268 |
| RBC | AA | 0.01 | 0.011 | 0.010 | 0.294 | -0.031 | 0.058 | 0.684 | - | - | - | 0.018 | 0.582 | 0.711 | 0.767 | 0.008 | 0.020 | 0.670 | 3 | 287 |
| RBC | AA | 0.1 | -0.004 | 0.008 | 0.580 | -0.024 | 0.042 | 0.599 | - | - | - | 0.008 | 0.651 | 0.876 | 0.821 | -0.012 | 0.014 | 0.399 | 7 | 584 |
| RBC | AA | 0.3 | 0.001 | 0.004 | 0.754 | -0.002 | 0.024 | 0.946 | - | - | - | 0.001 | 0.899 | 0.991 | 0.983 | 0.002 | 0.010 | 0.873 | 14 | 1440 |
| Plasma | DPA-n3 | 0.001 | -0.005 | 0.009 | 0.579 | -0.027 | 0.041 | 0.628 | - | - | - | 0.007 | 0.650 | 0.809 | 0.828 | -0.007 | 0.020 | 0.716 | 3 | 835 |
| Plasma | DPA-n3 | 0.01 | -0.005 | 0.009 | 0.577 | -0.027 | 0.041 | 0.627 | - | - | - | 0.007 | 0.648 | 0.809 | 0.836 | -0.007 | 0.019 | 0.708 | 3 | 937 |
| Plasma | DPA-n3 | 0.1 | -0.009 | 0.009 | 0.293 | -0.035 | 0.026 | 0.202 | - | - | - | 0.008 | 0.273 | 0.949 | 0.970 | -0.006 | 0.016 | 0.731 | 14 | 1506 |
| Plasma | DPA-n3 | 0.3 | -0.009 | 0.007 | 0.224 | -0.006 | 0.019 | 0.741 | - | - | - | -0.001 | 0.900 | 0.883 | 0.852 | -0.006 | 0.012 | 0.582 | 29 | 3466 |
| RBC | DPA-n3 | 0.001 | -0.014 | 0.001 | 6.05e-136 | - | - | - | - | - | - | - | - | - | - | - | - | - | 2 | 62 |
| RBC | DPA-n3 | 0.01 | -0.014 | 0.001 | 6.05e-136 | - | - | - | - | - | - | - | - | - | - | - | - | - | 2 | 62 |
| RBC | DPA-n3 | 0.1 | -0.014 | 0.001 | 6.05e-136 | - | - | - | - | - | - | - | - | - | - | - | - | - | 2 | 62 |
| RBC | DPA-n3 | 0.3 | -0.014 | 0.001 | 6.05e-136 | - | - | - | - | - | - | - | - | - | - | - | - | - | 2 | 62 |
| RBC | DTA | 0.001 | - | - | - | - | - | - | -0.009 | 0.036 | 0.794 | - | - | - | - | - | - | - | 1 | 80 |
| RBC | DTA | 0.01 | - | - | - | - | - | - | -0.009 | 0.036 | 0.794 | - | - | - | - | - | - | - | 1 | 80 |
| RBC | DTA | 0.1 | - | - | - | - | - | - | -0.009 | 0.036 | 0.794 | - | - | - | - | - | - | - | 1 | 80 |
| RBC | DTA | 0.3 | 0.003 | 0.017 | 0.858 | - | - | - | - | - | - | - | - | - | - | - | - | - | 2 | 114 |
| Plasma | DHA | 0.001 | - | - | - | - | - | - | 0.042 | 0.075 | 0.574 | - | - | - | - | - | - | - | 1 | 65 |
| Plasma | DHA | 0.01 | - | - | - | - | - | - | 0.042 | 0.075 | 0.574 | - | - | - | - | - | - | - | 1 | 64 |
| Plasma | DHA | 0.1 | - | - | - | - | - | - | 0.042 | 0.075 | 0.574 | - | - | - | - | - | - | - | 1 | 64 |
| Plasma | DHA | 0.3 | 0.049 | 0.010 | 1.56e-06 | - | - | - | - | - | - | - | - | - | - | - | - | - | 2 | 95 |

PUFA, polyunsaturated fatty acid; RBC, red blood cell; ALA, α-linolenic acid; LA, linoleic acid; GLA, γ-linolenic acid; DGLA, dihomo-γ-linolenic acid; AA, arachidonic acid; DPA-n3, docosapentaenoic acid; DTA, docosatetraenoic acid; DHA, docosahexaenoic acid; β, causal effect size; SE, standard error; IVW_MRE, inverse-variance weighted random-effects model; Egger, MR-Egger; nsnps, number of SNPs retained for this analysis.

**Supplementary Table 10.** Forward Mendelian randomization estimates of associations of genetically predicted polyunsaturated fatty acids with COVID-19 severity based on the release 4 HGI A2.

|  |  |  | **IVW_MRE** | | | **MR-Egger** | | | **Wald ratio** | | | **MR-Egger intercept** | | **Heterogeneity test** | | **Weighted median** | | |  |  |
| --- | --- | --- | --- | --- | --- | --- | --- | --- | --- | --- | --- | --- | --- | --- | --- | --- | --- | --- | --- | --- |
| **Group name** | **PUFA** | **R^2^** | **β** | **SE** | ***P*** | **β** | **SE** | ***P*** | **β** | **SE** | ***P*** | **β** | ***P*** | ***P*_IVW** | ***P*_Egger** | **β** | **SE** | ***P*** | **nsnps** | ***F*-statistic** |
| Plasma | ALA | 0.001 | - | - | - | - | - | - | 0.067 | 0.092 | 0.467 | - | - | - | - | - | - | - | 1 | 256 |
| Plasma | ALA | 0.01 | - | - | - | - | - | - | 0.067 | 0.093 | 0.467 | - | - | - | - | - | - | - | 1 | 312 |
| Plasma | ALA | 0.1 | 0.056 | 0.049 | 0.247 | -0.092 | 0.177 | 0.653 | - | - | - | 0.041 | 0.451 | 0.739 | 0.820 | 0.045 | 0.084 | 0.596 | 4 | 436 |
| Plasma | ALA | 0.3 | 0.087 | 0.049 | 0.075 | -0.130 | 0.137 | 0.364 | - | - | - | 0.054 | 0.120 | 0.398 | 0.551 | 0.069 | 0.066 | 0.295 | 13 | 1107 |
| RBC | ALA | 0.001 | - | - | - | - | - | - | -0.070 | 0.173 | 0.687 | - | - | - | - | - | - | - | 1 | 31 |
| RBC | ALA | 0.01 | - | - | - | - | - | - | -0.070 | 0.173 | 0.687 | - | - | - | - | - | - | - | 1 | 31 |
| RBC | ALA | 0.1 | - | - | - | - | - | - | -0.070 | 0.173 | 0.687 | - | - | - | - | - | - | - | 1 | 31 |
| RBC | ALA | 0.3 | - | - | - | - | - | - | -0.070 | 0.173 | 0.687 | - | - | - | - | - | - | - | 1 | 31 |
| Plasma | LA | 0.001 | 0.050 | 0.030 | 0.093 | 0.001 | 0.083 | 0.993 | - | - | - | 0.021 | 0.590 | 0.725 | 0.777 | 0.048 | 0.052 | 0.351 | 3 | 1329 |
| RBC | LA | 0.001 | - | - | - | - | - | - | 0.037 | 0.081 | 0.645 | - | - | - | - | - | - | - | 1 | 133 |
| RBC | LA | 0.01 | - | - | - | - | - | - | 0.037 | 0.081 | 0.645 | - | - | - | - | - | - | - | 1 | 133 |
| RBC | LA | 0.1 | 0.059 | 0.038 | 0.121 | - | - | - | - | - | - | - | - | - | - | - | - | - | 2 | 175 |
| RBC | LA | 0.3 | 0.059 | 0.045 | 0.185 | -0.177 | 0.221 | 0.448 | - | - | - | 0.070 | 0.311 | 0.312 | 0.332 | 0.041 | 0.057 | 0.468 | 9 | 566 |
| Plasma | GLA | 0.001 | -0.051 | 0.102 | 0.614 | - | - | - | - | - | - | - | - | - | - | - | - | - | 2 | 293 |
| RBC | GLA | 0.001 | - | - | - | - | - | - | 0.056 | 0.139 | 0.687 | - | - | - | - | - | - | - | 1 | 49 |
| RBC | GLA | 0.01 | - | - | - | - | - | - | 0.056 | 0.139 | 0.687 | - | - | - | - | - | - | - | 1 | 49 |
| RBC | GLA | 0.1 | - | - | - | - | - | - | 0.056 | 0.139 | 0.687 | - | - | - | - | - | - | - | 1 | 49 |
| RBC | GLA | 0.3 | - | - | - | - | - | - | 0.056 | 0.139 | 0.687 | - | - | - | - | - | - | - | 1 | 49 |
| Plasma | DGLA | 0.001 | 0.057 | 0.024 | 0.016 | - | - | - | - | - | - | - | - | - | - | - | - | - | 2 | 1417 |
| RBC | DGLA | 0.001 | -0.003 | 0.109 | 0.975 | - | - | - | - | - | - | - | - | - | - | - | - | - | 2 | 542 |
| RBC | DGLA | 0.01 | 0.034 | 0.064 | 0.598 | -0.130 | 0.142 | 0.456 | - | - | - | 0.095 | 0.334 | 0.021 | 0.068 | 0.053 | 0.052 | 0.303 | 4 | 788 |
| RBC | DGLA | 0.1 | 0.052 | 0.023 | 0.021 | 0.022 | 0.053 | 0.690 | - | - | - | 0.020 | 0.538 | 0.229 | 0.198 | 0.037 | 0.028 | 0.187 | 14 | 2375 |
| RBC | DGLA | 0.3 | 0.044 | 0.014 | 0.002 | 0.001 | 0.034 | 0.974 | - | - | - | 0.028 | 0.185 | 0.299 | 0.345 | 0.028 | 0.020 | 0.166 | 26 | 5970 |
| Plasma | AA | 0.001 | -0.022 | 0.029 | 0.440 | - | - | - | - | - | - | - | - | - | - | - | - | - | 2 | 4616 |
| RBC | AA | 0.001 | 4.31e-04 | 0.067 | 0.995 | -0.195 | 0.215 | 0.532 | - | - | - | 0.077 | 0.514 | 0.285 | 0.253 | -0.002 | 0.073 | 0.978 | 3 | 268 |
| RBC | AA | 0.01 | 0.005 | 0.067 | 0.937 | -0.146 | 0.223 | 0.630 | - | - | - | 0.063 | 0.601 | 0.275 | 0.193 | 0.005 | 0.068 | 0.939 | 3 | 287 |
| RBC | AA | 0.1 | -0.099 | 0.042 | 0.017 | -0.087 | 0.145 | 0.574 | - | - | - | -0.005 | 0.935 | 0.358 | 0.252 | -0.076 | 0.052 | 0.144 | 7 | 584 |
| RBC | AA | 0.3 | -0.069 | 0.028 | 0.014 | 0.004 | 0.086 | 0.966 | - | - | - | -0.030 | 0.387 | 0.176 | 0.172 | -0.059 | 0.034 | 0.083 | 14 | 1440 |
| Plasma | DPA-n3 | 0.001 | -0.089 | 0.053 | 0.093 | -0.022 | 0.157 | 0.913 | - | - | - | -0.023 | 0.717 | 0.424 | 0.237 | -0.086 | 0.061 | 0.158 | 3 | 835 |
| Plasma | DPA-n3 | 0.01 | -0.090 | 0.053 | 0.089 | -0.024 | 0.157 | 0.903 | - | - | - | -0.022 | 0.725 | 0.430 | 0.238 | -0.087 | 0.059 | 0.141 | 3 | 937 |
| Plasma | DPA-n3 | 0.1 | -0.105 | 0.039 | 0.006 | 0.004 | 0.086 | 0.963 | - | - | - | -0.034 | 0.171 | 0.614 | 0.716 | -0.054 | 0.057 | 0.350 | 14 | 1506 |
| Plasma | DPA-n3 | 0.3 | -0.101 | 0.029 | 0.001 | -0.009 | 0.064 | 0.894 | - | - | - | -0.028 | 0.120 | 0.437 | 0.522 | -0.052 | 0.042 | 0.218 | 29 | 3466 |
| RBC | DPA-n3 | 0.001 | -0.178 | 0.199 | 0.370 | - | - | - | - | - | - | - | - | - | - | - | - | - | 2 | 62 |
| RBC | DPA-n3 | 0.01 | -0.178 | 0.199 | 0.370 | - | - | - | - | - | - | - | - | - | - | - | - | - | 2 | 62 |
| RBC | DPA-n3 | 0.1 | -0.178 | 0.199 | 0.370 | - | - | - | - | - | - | - | - | - | - | - | - | - | 2 | 62 |
| RBC | DPA-n3 | 0.3 | -0.178 | 0.199 | 0.370 | - | - | - | - | - | - | - | - | - | - | - | - | - | 2 | 62 |
| RBC | DTA | 0.001 | - | - | - | - | - | - | -0.052 | 0.108 | 0.632 | - | - | - | - | - | - | - | 1 | 80 |
| RBC | DTA | 0.01 | - | - | - | - | - | - | -0.052 | 0.108 | 0.632 | - | - | - | - | - | - | - | 1 | 80 |
| RBC | DTA | 0.1 | - | - | - | - | - | - | -0.052 | 0.108 | 0.632 | - | - | - | - | - | - | - | 1 | 80 |
| RBC | DTA | 0.3 | -0.091 | 0.068 | 0.182 | - | - | - | - | - | - | - | - | - | - | - | - | - | 2 | 114 |
| Plasma | DHA | 0.001 | - | - | - | - | - | - | 0.491 | 0.217 | 0.024 | - | - | - | - | - | - | - | 1 | 65 |
| Plasma | DHA | 0.01 | - | - | - | - | - | - | 0.490 | 0.217 | 0.024 | - | - | - | - | - | - | - | 1 | 64 |
| Plasma | DHA | 0.1 | - | - | - | - | - | - | 0.490 | 0.217 | 0.024 | - | - | - | - | - | - | - | 1 | 64 |
| Plasma | DHA | 0.3 | 0.401 | 0.164 | 0.014 | - | - | - | - | - | - | - | - | - | - | - | - | - | 2 | 95 |

PUFA, polyunsaturated fatty acid; RBC, red blood cell; ALA, α-linolenic acid; LA, linoleic acid; GLA, γ-linolenic acid; DGLA, dihomo-γ-linolenic acid; AA, arachidonic acid; DPA-n3, docosapentaenoic acid; DTA, docosatetraenoic acid; DHA, docosahexaenoic acid; β, causal effect size; SE, standard error; IVW_MRE, inverse-variance weighted random-effects model; Egger, MR-Egger; nsnps, number of SNPs retained for this analysis.

**Supplementary Table 11.** Forward Mendelian randomization estimates of associations of genetically predicted polyunsaturated fatty acids with COVID-19 severity based on the release 4 HGI B2.

|  |  |  | **IVW_MRE** | | | **MR-Egger** | | | **Wald ratio** | | | **MR-Egger intercept** | | **Heterogeneity test** | | **Weighted median** | | |  |  |
| --- | --- | --- | --- | --- | --- | --- | --- | --- | --- | --- | --- | --- | --- | --- | --- | --- | --- | --- | --- | --- |
| **Group name** | **PUFA** | **R^2^** | **β** | **SE** | ***P*** | **β** | **SE** | ***P*** | **β** | **SE** | ***P*** | **β** | ***P*** | ***P*_IVW** | ***P*_Egger** | **β** | **SE** | ***P*** | **nsnps** | ***F*-statistic** |
| Plasma | ALA | 0.001 | - | - | - | - | - | - | 0.105 | 0.076 | 0.166 | - | - | - | - | - | - | - | 1 | 256 |
| Plasma | ALA | 0.01 | - | - | - | - | - | - | 0.106 | 0.076 | 0.166 | - | - | - | - | - | - | - | 1 | 312 |
| Plasma | ALA | 0.1 | 0.136 | 0.082 | 0.096 | -0.040 | 0.201 | 0.859 | - | - | - | 0.050 | 0.436 | 0.124 | 0.141 | 0.130 | 0.071 | 0.067 | 4 | 436 |
| Plasma | ALA | 0.3 | 0.150 | 0.040 | 1.44e-04 | -0.117 | 0.109 | 0.309 | - | - | - | 0.066 | 0.025 | 0.340 | 0.825 | 0.108 | 0.050 | 0.032 | 13 | 1107 |
| RBC | ALA | 0.001 | - | - | - | - | - | - | 0.092 | 0.146 | 0.526 | - | - | - | - | - | - | - | 1 | 31 |
| RBC | ALA | 0.01 | - | - | - | - | - | - | 0.092 | 0.146 | 0.526 | - | - | - | - | - | - | - | 1 | 31 |
| RBC | ALA | 0.1 | - | - | - | - | - | - | 0.092 | 0.146 | 0.526 | - | - | - | - | - | - | - | 1 | 31 |
| RBC | ALA | 0.3 | - | - | - | - | - | - | 0.092 | 0.146 | 0.526 | - | - | - | - | - | - | - | 1 | 31 |
| Plasma | LA | 0.001 | 0.073 | 0.034 | 0.032 | 0.011 | 0.070 | 0.900 | - | - | - | 0.027 | 0.463 | 0.531 | 0.969 | 0.071 | 0.043 | 0.098 | 3 | 1329 |
| RBC | LA | 0.001 | - | - | - | - | - | - | 0.118 | 0.067 | 0.079 | - | - | - | - | - | - | - | 1 | 133 |
| RBC | LA | 0.01 | - | - | - | - | - | - | 0.118 | 0.067 | 0.079 | - | - | - | - | - | - | - | 1 | 133 |
| RBC | LA | 0.1 | 0.138 | 0.035 | 9.47e-05 | - | - | - | - | - | - | - | - | - | - | - | - | - | 2 | 175 |
| RBC | LA | 0.3 | 0.123 | 0.019 | 1.84e-10 | -0.025 | 0.172 | 0.888 | - | - | - | 0.044 | 0.410 | 0.950 | 0.962 | 0.122 | 0.041 | 0.003 | 9 | 566 |
| Plasma | GLA | 0.001 | -0.091 | 0.113 | 0.417 | - | - | - | - | - | - | - | - | - | - | - | - | - | 2 | 293 |
| RBC | GLA | 0.001 | - | - | - | - | - | - | -0.074 | 0.117 | 0.526 | - | - | - | - | - | - | - | 1 | 49 |
| RBC | GLA | 0.01 | - | - | - | - | - | - | -0.074 | 0.117 | 0.526 | - | - | - | - | - | - | - | 1 | 49 |
| RBC | GLA | 0.1 | - | - | - | - | - | - | -0.074 | 0.117 | 0.526 | - | - | - | - | - | - | - | 1 | 49 |
| RBC | GLA | 0.3 | - | - | - | - | - | - | -0.074 | 0.117 | 0.526 | - | - | - | - | - | - | - | 1 | 49 |
| Plasma | DGLA | 0.001 | 0.077 | 0.010 | 8.31e-14 | - | - | - | - | - | - | - | - | - | - | - | - | - | 2 | 1417 |
| RBC | DGLA | 0.001 | 0.048 | 0.044 | 0.271 | - | - | - | - | - | - | - | - | - | - | - | - | - | 2 | 542 |
| RBC | DGLA | 0.01 | 0.056 | 0.026 | 0.029 | -0.039 | 0.070 | 0.637 | - | - | - | 0.055 | 0.279 | 0.472 | 0.837 | 0.032 | 0.032 | 0.316 | 4 | 788 |
| RBC | DGLA | 0.1 | 0.043 | 0.017 | 0.010 | 0.036 | 0.040 | 0.394 | - | - | - | 0.005 | 0.841 | 0.396 | 0.324 | 0.032 | 0.022 | 0.138 | 14 | 2375 |
| RBC | DGLA | 0.3 | 0.051 | 0.010 | 1.07e-07 | 0.023 | 0.026 | 0.388 | - | - | - | 0.018 | 0.263 | 0.736 | 0.758 | 0.046 | 0.016 | 0.003 | 26 | 5970 |
| Plasma | AA | 0.001 | -0.036 | 0.030 | 0.221 | - | - | - | - | - | - | - | - | - | - | - | - | - | 2 | 4616 |
| RBC | AA | 0.001 | -0.080 | 0.041 | 0.053 | -0.099 | 0.188 | 0.692 | - | - | - | 0.008 | 0.933 | 0.481 | 0.229 | -0.054 | 0.060 | 0.369 | 3 | 268 |
| RBC | AA | 0.01 | -0.078 | 0.041 | 0.058 | -0.076 | 0.176 | 0.740 | - | - | - | -0.001 | 0.994 | 0.470 | 0.219 | -0.051 | 0.056 | 0.360 | 3 | 287 |
| RBC | AA | 0.1 | -0.116 | 0.019 | 1.50e-09 | -0.077 | 0.105 | 0.497 | - | - | - | -0.015 | 0.711 | 0.897 | 0.839 | -0.120 | 0.039 | 0.002 | 7 | 584 |
| RBC | AA | 0.3 | -0.084 | 0.013 | 5.54e-11 | 0.005 | 0.060 | 0.940 | - | - | - | -0.037 | 0.149 | 0.961 | 0.994 | -0.068 | 0.026 | 0.008 | 14 | 1440 |
| Plasma | DPA-n3 | 0.001 | -0.082 | 0.024 | 0.001 | -0.108 | 0.106 | 0.495 | - | - | - | 0.009 | 0.831 | 0.769 | 0.502 | -0.086 | 0.050 | 0.088 | 3 | 835 |
| Plasma | DPA-n3 | 0.01 | -0.082 | 0.024 | 0.001 | -0.109 | 0.107 | 0.492 | - | - | - | 0.009 | 0.824 | 0.775 | 0.512 | -0.086 | 0.050 | 0.086 | 3 | 937 |
| Plasma | DPA-n3 | 0.1 | -0.120 | 0.031 | 1.04e-04 | -0.033 | 0.069 | 0.642 | - | - | - | -0.027 | 0.169 | 0.620 | 0.724 | -0.078 | 0.048 | 0.100 | 14 | 1506 |
| Plasma | DPA-n3 | 0.3 | -0.116 | 0.021 | 5.54e-08 | -0.029 | 0.051 | 0.580 | - | - | - | -0.026 | 0.068 | 0.661 | 0.797 | -0.085 | 0.033 | 0.010 | 29 | 3466 |
| RBC | DPA-n3 | 0.001 | -0.162 | 0.028 | 1.19e-08 | - | - | - | - | - | - | - | - | - | - | - | - | - | 2 | 62 |
| RBC | DPA-n3 | 0.01 | -0.162 | 0.028 | 1.19e-08 | - | - | - | - | - | - | - | - | - | - | - | - | - | 2 | 62 |
| RBC | DPA-n3 | 0.1 | -0.162 | 0.028 | 1.19e-08 | - | - | - | - | - | - | - | - | - | - | - | - | - | 2 | 62 |
| RBC | DPA-n3 | 0.3 | -0.162 | 0.028 | 1.19e-08 | - | - | - | - | - | - | - | - | - | - | - | - | - | 2 | 62 |
| RBC | DTA | 0.001 | - | - | - | - | - | - | -0.103 | 0.088 | 0.244 | - | - | - | - | - | - | - | 1 | 80 |
| RBC | DTA | 0.01 | - | - | - | - | - | - | -0.103 | 0.088 | 0.244 | - | - | - | - | - | - | - | 1 | 80 |
| RBC | DTA | 0.1 | - | - | - | - | - | - | -0.103 | 0.088 | 0.244 | - | - | - | - | - | - | - | 1 | 80 |
| RBC | DTA | 0.3 | -0.152 | 0.077 | 0.048 | - | - | - | - | - | - | - | - | - | - | - | - | - | 2 | 114 |
| Plasma | DHA | 0.001 | - | - | - | - | - | - | 0.260 | 0.185 | 0.159 | - | - | - | - | - | - | - | 1 | 65 |
| Plasma | DHA | 0.01 | - | - | - | - | - | - | 0.260 | 0.184 | 0.159 | - | - | - | - | - | - | - | 1 | 64 |
| Plasma | DHA | 0.1 | - | - | - | - | - | - | 0.260 | 0.184 | 0.159 | - | - | - | - | - | - | - | 1 | 64 |
| Plasma | DHA | 0.3 | 0.270 | 0.018 | 1.86e-51 | - | - | - | - | - | - | - | - | - | - | - | - | - | 2 | 95 |

PUFA, polyunsaturated fatty acid; RBC, red blood cell; ALA, α-linolenic acid; LA, linoleic acid; GLA, γ-linolenic acid; DGLA, dihomo-γ-linolenic acid; AA, arachidonic acid; DPA-n3, docosapentaenoic acid; DTA, docosatetraenoic acid; DHA, docosahexaenoic acid; β, causal effect size; SE, standard error; IVW_MRE, inverse-variance weighted random-effects model; Egger, MR-Egger; nsnps, number of SNPs retained for this analysis.

**Supplementary Table 12.** Forward Mendelian randomization estimates of associations of genetically predicted polyunsaturated fatty acids with COVID-19 severity based on the release 4 HGI B1.

|  |  |  | **IVW_MRE** | | | **MR-Egger** | | | **Wald ratio** | | | **MR-Egger intercept** | | **Heterogeneity test** | | **Weighted median** | | |  |  |
| --- | --- | --- | --- | --- | --- | --- | --- | --- | --- | --- | --- | --- | --- | --- | --- | --- | --- | --- | --- | --- |
| **Group name** | **PUFA** | **R^2^** | **β** | **SE** | ***P*** | **β** | **SE** | ***P*** | **β** | **SE** | ***P*** | **β** | ***P*** | ***P*_IVW** | ***P*_Egger** | **β** | **SE** | ***P*** | **nsnps** | ***F*-statistic** |
| Plasma | ALA | 0.001 | - | - | - | - | - | - | 0.080 | 0.137 | 0.558 | - | - | - | - | - | - | - | 1 | 256 |
| Plasma | ALA | 0.01 | - | - | - | - | - | - | 0.081 | 0.138 | 0.558 | - | - | - | - | - | - | - | 1 | 312 |
| Plasma | ALA | 0.1 | 0.174 | 0.155 | 0.261 | -0.085 | 0.408 | 0.855 | - | - | - | 0.073 | 0.558 | 0.068 | 0.057 | 0.124 | 0.130 | 0.338 | 4 | 436 |
| Plasma | ALA | 0.3 | 0.142 | 0.072 | 0.050 | -0.119 | 0.210 | 0.583 | - | - | - | 0.064 | 0.214 | 0.100 | 0.140 | 0.052 | 0.083 | 0.531 | 13 | 1107 |
| RBC | ALA | 0.001 | - | - | - | - | - | - | 0.125 | 0.248 | 0.615 | - | - | - | - | - | - | - | 1 | 31 |
| RBC | ALA | 0.01 | - | - | - | - | - | - | 0.125 | 0.248 | 0.615 | - | - | - | - | - | - | - | 1 | 31 |
| RBC | ALA | 0.1 | - | - | - | - | - | - | 0.125 | 0.248 | 0.615 | - | - | - | - | - | - | - | 1 | 31 |
| RBC | ALA | 0.3 | - | - | - | - | - | - | 0.125 | 0.248 | 0.615 | - | - | - | - | - | - | - | 1 | 31 |
| Plasma | LA | 0.001 | 0.036 | 0.028 | 0.198 | 0.085 | 0.127 | 0.624 | - | - | - | -0.021 | 0.712 | 0.877 | 0.871 | 0.037 | 0.080 | 0.640 | 3 | 1329 |
| RBC | LA | 0.001 | - | - | - | - | - | - | 0.051 | 0.113 | 0.651 | - | - | - | - | - | - | - | 1 | 133 |
| RBC | LA | 0.01 | - | - | - | - | - | - | 0.051 | 0.113 | 0.651 | - | - | - | - | - | - | - | 1 | 133 |
| RBC | LA | 0.1 | 0.113 | 0.110 | 0.306 | - | - | - | - | - | - | - | - | - | - | - | - | - | 2 | 175 |
| RBC | LA | 0.3 | 0.146 | 0.049 | 0.003 | -0.098 | 0.296 | 0.750 | - | - | - | 0.072 | 0.430 | 0.598 | 0.570 | 0.145 | 0.076 | 0.057 | 9 | 566 |
| Plasma | GLA | 0.001 | -0.103 | 0.017 | 2.42e-09 | - | - | - | - | - | - | - | - | - | - | - | - | - | 2 | 293 |
| RBC | GLA | 0.001 | - | - | - | - | - | - | -0.100 | 0.199 | 0.615 | - | - | - | - | - | - | - | 1 | 49 |
| RBC | GLA | 0.01 | - | - | - | - | - | - | -0.100 | 0.199 | 0.615 | - | - | - | - | - | - | - | 1 | 49 |
| RBC | GLA | 0.1 | - | - | - | - | - | - | -0.100 | 0.199 | 0.615 | - | - | - | - | - | - | - | 1 | 49 |
| RBC | GLA | 0.3 | - | - | - | - | - | - | -0.100 | 0.199 | 0.615 | - | - | - | - | - | - | - | 1 | 49 |
| Plasma | DGLA | 0.001 | 0.025 | 0.048 | 0.595 | - | - | - | - | - | - | - | - | - | - | - | - | - | 2 | 1417 |
| RBC | DGLA | 0.001 | -0.015 | 0.013 | 0.231 | - | - | - | - | - | - | - | - | - | - | - | - | - | 2 | 542 |
| RBC | DGLA | 0.01 | 0.005 | 0.028 | 0.855 | -0.076 | 0.117 | 0.582 | - | - | - | 0.048 | 0.530 | 0.754 | 0.731 | -0.008 | 0.050 | 0.867 | 4 | 788 |
| RBC | DGLA | 0.1 | 0.041 | 0.032 | 0.202 | 0.062 | 0.081 | 0.458 | - | - | - | -0.013 | 0.778 | 0.175 | 0.134 | 0.046 | 0.038 | 0.224 | 14 | 2375 |
| RBC | DGLA | 0.3 | 0.041 | 0.020 | 0.034 | 0.055 | 0.051 | 0.293 | - | - | - | -0.008 | 0.780 | 0.137 | 0.111 | 0.038 | 0.025 | 0.130 | 26 | 5970 |
| Plasma | AA | 0.001 | -0.031 | 0.014 | 0.023 | - | - | - | - | - | - | - | - | - | - | - | - | - | 2 | 4616 |
| RBC | AA | 0.001 | -0.216 | 0.082 | 0.008 | 0.061 | 0.251 | 0.848 | - | - | - | -0.113 | 0.454 | 0.312 | 0.320 | -0.207 | 0.088 | 0.019 | 3 | 268 |
| RBC | AA | 0.01 | -0.218 | 0.080 | 0.006 | 0.055 | 0.235 | 0.854 | - | - | - | -0.114 | 0.435 | 0.326 | 0.393 | -0.209 | 0.088 | 0.017 | 3 | 287 |
| RBC | AA | 0.1 | -0.112 | 0.062 | 0.073 | 0.079 | 0.225 | 0.738 | - | - | - | -0.074 | 0.416 | 0.172 | 0.167 | -0.039 | 0.069 | 0.577 | 7 | 584 |
| RBC | AA | 0.3 | -0.068 | 0.035 | 0.051 | 0.107 | 0.103 | 0.322 | - | - | - | -0.071 | 0.101 | 0.319 | 0.474 | -0.042 | 0.046 | 0.361 | 14 | 1440 |
| Plasma | DPA-n3 | 0.001 | -0.117 | 0.071 | 0.097 | -0.018 | 0.177 | 0.936 | - | - | - | -0.033 | 0.637 | 0.495 | 0.319 | -0.087 | 0.087 | 0.317 | 3 | 835 |
| Plasma | DPA-n3 | 0.01 | -0.118 | 0.071 | 0.093 | -0.021 | 0.177 | 0.926 | - | - | - | -0.032 | 0.643 | 0.501 | 0.320 | -0.089 | 0.093 | 0.340 | 3 | 937 |
| Plasma | DPA-n3 | 0.1 | -0.121 | 0.065 | 0.061 | 0.003 | 0.131 | 0.980 | - | - | - | -0.039 | 0.296 | 0.143 | 0.160 | -0.096 | 0.079 | 0.226 | 14 | 1506 |
| Plasma | DPA-n3 | 0.3 | -0.124 | 0.042 | 0.003 | 0.008 | 0.094 | 0.932 | - | - | - | -0.040 | 0.132 | 0.060 | 0.092 | -0.053 | 0.055 | 0.336 | 29 | 3466 |
| RBC | DPA-n3 | 0.001 | -0.240 | 0.048 | 6.15e-07 | - | - | - | - | - | - | - | - | - | - | - | - | - | 2 | 62 |
| RBC | DPA-n3 | 0.01 | -0.240 | 0.048 | 6.15e-07 | - | - | - | - | - | - | - | - | - | - | - | - | - | 2 | 62 |
| RBC | DPA-n3 | 0.1 | -0.240 | 0.048 | 6.15e-07 | - | - | - | - | - | - | - | - | - | - | - | - | - | 2 | 62 |
| RBC | DPA-n3 | 0.3 | -0.240 | 0.048 | 6.15e-07 | - | - | - | - | - | - | - | - | - | - | - | - | - | 2 | 62 |
| RBC | DTA | 0.001 | - | - | - | - | - | - | -0.089 | 0.154 | 0.563 | - | - | - | - | - | - | - | 1 | 80 |
| RBC | DTA | 0.01 | - | - | - | - | - | - | -0.089 | 0.154 | 0.563 | - | - | - | - | - | - | - | 1 | 80 |
| RBC | DTA | 0.1 | - | - | - | - | - | - | -0.089 | 0.154 | 0.563 | - | - | - | - | - | - | - | 1 | 80 |
| RBC | DTA | 0.3 | -0.232 | 0.190 | 0.223 | - | - | - | - | - | - | - | - | - | - | - | - | - | 2 | 114 |
| Plasma | DHA | 0.001 | - | - | - | - | - | - | 0.468 | 0.329 | 0.155 | - | - | - | - | - | - | - | 1 | 65 |
| Plasma | DHA | 0.01 | - | - | - | - | - | - | 0.467 | 0.328 | 0.155 | - | - | - | - | - | - | - | 1 | 64 |
| Plasma | DHA | 0.1 | - | - | - | - | - | - | 0.467 | 0.328 | 0.155 | - | - | - | - | - | - | - | 1 | 64 |
| Plasma | DHA | 0.3 | 0.362 | 0.145 | 0.013 | - | - | - | - | - | - | - | - | - | - | - | - | - | 2 | 95 |

PUFA, polyunsaturated fatty acid; RBC, red blood cell; ALA, α-linolenic acid; LA, linoleic acid; GLA, γ-linolenic acid; DGLA, dihomo-γ-linolenic acid; AA, arachidonic acid; DPA-n3, docosapentaenoic acid; DTA, docosatetraenoic acid; DHA, docosahexaenoic acid; β, causal effect size; SE, standard error; IVW_MRE, inverse-variance weighted random-effects model; Egger, MR-Egger; nsnps, number of SNPs retained for this analysis.

**Supplementary Table 13.** Forward Mendelian randomization estimates of associations of genetically predicted polyunsaturated fatty acids with COVID-19 susceptibility based on the release 4 HGI C2.

|  |  |  | **IVW_MRE** | | | **MR-Egger** | | | **Wald ratio** | | | **MR-Egger intercept** | | **Heterogeneity test** | | **Weighted median** | | |  |  |
| --- | --- | --- | --- | --- | --- | --- | --- | --- | --- | --- | --- | --- | --- | --- | --- | --- | --- | --- | --- | --- |
| **Group name** | **PUFA** | **R^2^** | **β** | **SE** | ***P*** | **β** | **SE** | ***P*** | **β** | **SE** | ***P*** | **β** | ***P*** | ***P*_IVW** | ***P*_Egger** | **β** | **SE** | ***P*** | **nsnps** | ***F*-statistic** |
| Plasma | ALA | 0.001 | - | - | - | - | - | - | 0.005 | 0.047 | 0.908 | - | - | - | - | - | - | - | 1 | 256 |
| Plasma | ALA | 0.01 | - | - | - | - | - | - | 0.005 | 0.048 | 0.908 | - | - | - | - | - | - | - | 1 | 312 |
| Plasma | ALA | 0.1 | 0.003 | 0.011 | 0.782 | -0.012 | 0.086 | 0.904 | - | - | - | 0.004 | 0.868 | 0.957 | 0.870 | 0.007 | 0.039 | 0.861 | 4 | 436 |
| Plasma | ALA | 0.3 | -0.003 | 0.015 | 0.811 | -0.019 | 0.063 | 0.775 | - | - | - | 0.004 | 0.805 | 0.937 | 0.905 | -0.013 | 0.028 | 0.643 | 13 | 1107 |
| RBC | ALA | 0.001 | - | - | - | - | - | - | -0.023 | 0.090 | 0.796 | - | - | - | - | - | - | - | 1 | 31 |
| RBC | ALA | 0.01 | - | - | - | - | - | - | -0.023 | 0.090 | 0.796 | - | - | - | - | - | - | - | 1 | 31 |
| RBC | ALA | 0.1 | - | - | - | - | - | - | -0.023 | 0.090 | 0.796 | - | - | - | - | - | - | - | 1 | 31 |
| RBC | ALA | 0.3 | - | - | - | - | - | - | -0.023 | 0.090 | 0.796 | - | - | - | - | - | - | - | 1 | 31 |
| Plasma | LA | 0.001 | 0.009 | 0.028 | 0.740 | -0.029 | 0.044 | 0.625 | - | - | - | 0.017 | 0.465 | 0.326 | 0.318 | 0.003 | 0.027 | 0.900 | 3 | 1329 |
| RBC | LA | 0.001 | - | - | - | - | - | - | -0.003 | 0.042 | 0.949 | - | - | - | - | - | - | - | 1 | 133 |
| RBC | LA | 0.01 | - | - | - | - | - | - | -0.003 | 0.042 | 0.949 | - | - | - | - | - | - | - | 1 | 133 |
| RBC | LA | 0.1 | -0.002 | 0.001 | 0.168 | - | - | - | - | - | - | - | - | - | - | - | - | - | 2 | 175 |
| RBC | LA | 0.3 | -0.009 | 0.014 | 0.518 | 0.016 | 0.106 | 0.885 | - | - | - | -0.007 | 0.816 | 0.835 | 0.758 | -0.016 | 0.025 | 0.527 | 9 | 566 |
| Plasma | GLA | 0.001 | -0.005 | 0.004 | 0.230 | - | - | - | - | - | - | - | - | - | - | - | - | - | 2 | 293 |
| RBC | GLA | 0.001 | - | - | - | - | - | - | 0.019 | 0.072 | 0.796 | - | - | - | - | - | - | - | 1 | 49 |
| RBC | GLA | 0.01 | - | - | - | - | - | - | 0.019 | 0.072 | 0.796 | - | - | - | - | - | - | - | 1 | 49 |
| RBC | GLA | 0.1 | - | - | - | - | - | - | 0.019 | 0.072 | 0.796 | - | - | - | - | - | - | - | 1 | 49 |
| RBC | GLA | 0.3 | - | - | - | - | - | - | 0.019 | 0.072 | 0.796 | - | - | - | - | - | - | - | 1 | 49 |
| Plasma | DGLA | 0.001 | 0.003 | 0.001 | 1.64e-11 | - | - | - | - | - | - | - | - | - | - | - | - | - | 2 | 1417 |
| RBC | DGLA | 0.001 | 0.029 | 0.011 | 0.008 | - | - | - | - | - | - | - | - | - | - | - | - | - | 2 | 542 |
| RBC | DGLA | 0.01 | 0.017 | 0.015 | 0.270 | 0.032 | 0.046 | 0.556 | - | - | - | -0.009 | 0.751 | 0.429 | 0.273 | 0.022 | 0.019 | 0.240 | 4 | 788 |
| RBC | DGLA | 0.1 | 0.003 | 0.009 | 0.742 | -0.004 | 0.023 | 0.853 | - | - | - | 0.005 | 0.727 | 0.507 | 0.436 | -0.015 | 0.013 | 0.254 | 14 | 2375 |
| RBC | DGLA | 0.3 | -4.41e-04 | 0.005 | 0.936 | 0.001 | 0.016 | 0.965 | - | - | - | -0.001 | 0.938 | 0.800 | 0.755 | -0.009 | 0.009 | 0.306 | 26 | 5970 |
| Plasma | AA | 0.001 | -0.002 | 0.001 | 0.047 | - | - | - | - | - | - | - | - | - | - | - | - | - | 2 | 4616 |
| RBC | AA | 0.001 | -0.001 | 0.015 | 0.952 | 0.004 | 0.096 | 0.973 | - | - | - | -0.002 | 0.966 | 0.756 | 0.455 | -0.007 | 0.031 | 0.823 | 3 | 268 |
| RBC | AA | 0.01 | -0.007 | 0.019 | 0.705 | -0.019 | 0.087 | 0.865 | - | - | - | 0.005 | 0.911 | 0.634 | 0.345 | -0.010 | 0.032 | 0.767 | 3 | 287 |
| RBC | AA | 0.1 | -0.004 | 0.017 | 0.796 | -0.011 | 0.064 | 0.873 | - | - | - | 0.002 | 0.921 | 0.542 | 0.416 | 0.013 | 0.025 | 0.607 | 7 | 584 |
| RBC | AA | 0.3 | 0.003 | 0.010 | 0.772 | -0.003 | 0.037 | 0.941 | - | - | - | 0.002 | 0.877 | 0.792 | 0.727 | 0.007 | 0.016 | 0.651 | 14 | 1440 |
| Plasma | DPA-n3 | 0.001 | -0.001 | 0.024 | 0.961 | -0.049 | 0.063 | 0.580 | - | - | - | 0.016 | 0.549 | 0.509 | 0.432 | -0.007 | 0.030 | 0.810 | 3 | 835 |
| Plasma | DPA-n3 | 0.01 | -0.001 | 0.024 | 0.956 | -0.050 | 0.063 | 0.575 | - | - | - | 0.016 | 0.545 | 0.509 | 0.439 | -0.007 | 0.030 | 0.812 | 3 | 937 |
| Plasma | DPA-n3 | 0.1 | -0.005 | 0.015 | 0.717 | -0.036 | 0.040 | 0.384 | - | - | - | 0.010 | 0.392 | 0.902 | 0.905 | -0.006 | 0.026 | 0.834 | 14 | 1506 |
| Plasma | DPA-n3 | 0.3 | 0.001 | 0.010 | 0.937 | 0.010 | 0.030 | 0.736 | - | - | - | -0.003 | 0.729 | 0.955 | 0.943 | 0.017 | 0.018 | 0.358 | 29 | 3466 |
| RBC | DPA-n3 | 0.001 | -0.010 | 0.004 | 0.004 | - | - | - | - | - | - | - | - | - | - | - | - | - | 2 | 62 |
| RBC | DPA-n3 | 0.01 | -0.010 | 0.004 | 0.004 | - | - | - | - | - | - | - | - | - | - | - | - | - | 2 | 62 |
| RBC | DPA-n3 | 0.1 | -0.010 | 0.004 | 0.004 | - | - | - | - | - | - | - | - | - | - | - | - | - | 2 | 62 |
| RBC | DPA-n3 | 0.3 | -0.010 | 0.004 | 0.004 | - | - | - | - | - | - | - | - | - | - | - | - | - | 2 | 62 |
| RBC | DTA | 0.001 | - | - | - | - | - | - | 0.003 | 0.055 | 0.963 | - | - | - | - | - | - | - | 1 | 80 |
| RBC | DTA | 0.01 | - | - | - | - | - | - | 0.003 | 0.055 | 0.963 | - | - | - | - | - | - | - | 1 | 80 |
| RBC | DTA | 0.1 | - | - | - | - | - | - | 0.003 | 0.055 | 0.963 | - | - | - | - | - | - | - | 1 | 80 |
| RBC | DTA | 0.3 | 0.015 | 0.018 | 0.391 | - | - | - | - | - | - | - | - | - | - | - | - | - | 2 | 114 |
| Plasma | DHA | 0.001 | - | - | - | - | - | - | 0.046 | 0.114 | 0.689 | - | - | - | - | - | - | - | 1 | 65 |
| Plasma | DHA | 0.01 | - | - | - | - | - | - | 0.045 | 0.113 | 0.689 | - | - | - | - | - | - | - | 1 | 64 |
| Plasma | DHA | 0.1 | - | - | - | - | - | - | 0.045 | 0.113 | 0.689 | - | - | - | - | - | - | - | 1 | 64 |
| Plasma | DHA | 0.3 | 0.029 | 0.025 | 0.245 | - | - | - | - | - | - | - | - | - | - | - | - | - | 2 | 95 |

PUFA, polyunsaturated fatty acid; RBC, red blood cell; ALA, α-linolenic acid; LA, linoleic acid; GLA, γ-linolenic acid; DGLA, dihomo-γ-linolenic acid; AA, arachidonic acid; DPA-n3, docosapentaenoic acid; DTA, docosatetraenoic acid; DHA, docosahexaenoic acid; β, causal effect size; SE, standard error; IVW_MRE, inverse-variance weighted random-effects model; Egger, MR-Egger; nsnps, number of SNPs retained for this analysis.

**Supplementary Table 14.** Reverse Mendelian randomization estimates of associations of genetically predicted COVID-19 severity with polyunsaturated fatty acids based on the release 5 HGI A2 (COVID-19 SNP *P* < 5e-8).

|  |  | **IVW_MRE** | | | **MR-Egger** | | | **MR-Egger intercept** | | **Heterogeneity test** | | **Weighted median** | | |  |  |
| --- | --- | --- | --- | --- | --- | --- | --- | --- | --- | --- | --- | --- | --- | --- | --- | --- |
| **Group name** | **PUFA** | **β** | **SE** | ***P*** | **β** | **SE** | ***P*** | **β** | ***P*** | ***P*_IVW** | ***P*_Egger** | **β** | **SE** | ***P*** | **nsnps** | ***F*-statistic** |
| Plasma | ALA | 0.02 | 0.009 | 0.019 | - | - | - | - | - | - | - | - | - | - | 2 | 119 |
| RBC | ALA | 0.008 | 0.009 | 0.37 | 0.019 | 0.023 | 0.43 | -0.004 | 0.596 | 0.544 | 0.464 | 0.013 | 0.012 | 0.31 | 8 | 536 |
| RBC | LA | -0.004 | 0.003 | 0.241 | -0.014 | 0.009 | 0.146 | 0.003 | 0.229 | 0.527 | 0.634 | -0.005 | 0.004 | 0.233 | 8 | 536 |
| RBC | GLA | -0.006 | 0.006 | 0.279 | 0.015 | 0.033 | 0.668 | -0.007 | 0.515 | 0.991 | 0.994 | -0.008 | 0.016 | 0.603 | 8 | 536 |
| RBC | DGLA | -0.004 | 0.004 | 0.29 | -0.01 | 0.012 | 0.444 | 0.002 | 0.601 | 0.802 | 0.744 | -0.004 | 0.006 | 0.494 | 8 | 536 |
| RBC | AA | 0.001 | 0.002 | 0.595 | 0.006 | 0.006 | 0.335 | -0.002 | 0.373 | 0.747 | 0.764 | 0.001 | 0.003 | 0.799 | 8 | 536 |
| Plasma | DPA-n3 | -0.031 | 0.053 | 0.554 | - | - | - | - | - | - | - | - | - | - | 2 | 119 |
| RBC | DPA-n3 | 0.004 | 0.003 | 0.165 | -0.002 | 0.009 | 0.862 | 0.002 | 0.539 | 0.8 | 0.757 | 0.003 | 0.005 | 0.466 | 8 | 536 |
| RBC | DTA | 0.007 | 0.005 | 0.152 | -0.002 | 0.014 | 0.906 | 0.003 | 0.509 | 0.58 | 0.523 | -0.001 | 0.007 | 0.914 | 8 | 536 |
| Plasma | DHA | -0.061 | 0.164 | 0.71 | - | - | - | - | - | - | - | - | - | - | 2 | 119 |

PUFA, polyunsaturated fatty acid; RBC, red blood cell; ALA, α-linolenic acid; LA, linoleic acid; GLA, γ-linolenic acid; DGLA, dihomo-γ-linolenic acid; AA, arachidonic acid; DPA-n3, docosapentaenoic acid; DTA, docosatetraenoic acid; DHA, docosahexaenoic acid; β, causal effect size; SE, standard error; IVW_MRE, inverse-variance weighted random-effects model; Egger, MR-Egger; nsnps, number of SNPs retained for this analysis.

**Supplementary Table 15.** Reverse Mendelian randomization estimates of associations of genetically predicted COVID-19 severity with polyunsaturated fatty acids based on the release 5 HGI B2 (COVID-19 SNP *P* < 5e-8).

|  |  | **IVW_MRE** | | | **MR-Egger** | | | **MR-Egger intercept** | | **Heterogeneity test** | | **Weighted median** | | |  |  |
| --- | --- | --- | --- | --- | --- | --- | --- | --- | --- | --- | --- | --- | --- | --- | --- | --- |
| **Group name** | **PUFA** | **β** | **SE** | ***P*** | **β** | **SE** | ***P*** | **β** | ***P*** | ***P*_IVW** | ***P*_Egger** | **β** | **SE** | ***P*** | **nsnps** | ***F*-statistic** |
| Plasma | ALA | 0.005 | 0.013 | 0.706 | - | - | - | - | - | - | - | - | - | - | 2 | 94 |
| RBC | ALA | 0.010 | 0.019 | 0.606 | 0.035 | 0.040 | 0.453 | -0.006 | 0.527 | 0.132 | 0.109 | 0.021 | 0.016 | 0.192 | 5 | 438 |
| RBC | LA | -0.005 | 0.005 | 0.302 | -0.016 | 0.011 | 0.239 | 0.002 | 0.350 | 0.405 | 0.426 | -0.008 | 0.006 | 0.208 | 5 | 438 |
| RBC | GLA | -0.017 | 0.010 | 0.109 | 0.019 | 0.042 | 0.672 | -0.008 | 0.392 | 0.901 | 0.996 | -0.019 | 0.025 | 0.439 | 5 | 438 |
| RBC | DGLA | -0.003 | 0.006 | 0.595 | -0.004 | 0.015 | 0.831 | 1.08e-04 | 0.974 | 0.656 | 0.487 | -0.005 | 0.009 | 0.547 | 5 | 438 |
| RBC | AA | 0.001 | 0.003 | 0.806 | 0.003 | 0.008 | 0.752 | -4.23e-04 | 0.800 | 0.457 | 0.314 | 2.77e-05 | 0.004 | 0.995 | 5 | 438 |
| Plasma | DPA-n3 | 0.022 | 0.012 | 0.062 | - | - | - | - | - | - | - | - | - | - | 2 | 94 |
| RBC | DPA-n3 | 0.006 | 0.004 | 0.125 | 0.004 | 0.012 | 0.732 | 4.95e-04 | 0.843 | 0.686 | 0.528 | 0.005 | 0.007 | 0.423 | 5 | 438 |
| RBC | DTA | 0.005 | 0.009 | 0.551 | -0.015 | 0.017 | 0.430 | 0.005 | 0.257 | 0.339 | 0.461 | -0.002 | 0.010 | 0.805 | 5 | 438 |
| Plasma | DHA | -0.107 | 0.044 | 0.014 | - | - | - | - | - | - | - | - | - | - | 2 | 94 |

PUFA, polyunsaturated fatty acid; RBC, red blood cell; ALA, α-linolenic acid; LA, linoleic acid; GLA, γ-linolenic acid; DGLA, dihomo-γ-linolenic acid; AA, arachidonic acid; DPA-n3, docosapentaenoic acid; DTA, docosatetraenoic acid; DHA, docosahexaenoic acid; β, causal effect size; SE, standard error; IVW_MRE, inverse-variance weighted random-effects model; Egger, MR-Egger; nsnps, number of SNPs retained for this analysis.

**Supplementary Table 16.** Reverse Mendelian randomization estimates of associations of genetically predicted COVID-19 severity with polyunsaturated fatty acids based on the release 5 HGI B1 (COVID-19 SNP *P* < 5e-8).

|  |  | **Wald ratio** | | |  |  |
| --- | --- | --- | --- | --- | --- | --- |
| **Group name** | **PUFA** | **β** | **SE** | ***P*** | **nsnps** | ***F*-statistic** |
| Plasma | ALA | 3.68E-04 | 0.005 | 0.939 | 1 | 34 |
| RBC | ALA | -0.025 | 0.029 | 0.402 | 1 | 34 |
| RBC | LA | -0.012 | 0.011 | 0.289 | 1 | 34 |
| RBC | GLA | 0.022 | 0.043 | 0.604 | 1 | 34 |
| RBC | DGLA | -0.003 | 0.016 | 0.844 | 1 | 34 |
| RBC | AA | 0.012 | 0.007 | 0.1 | 1 | 34 |
| Plasma | DPA-n3 | -0.001 | 0.016 | 0.946 | 1 | 34 |
| RBC | DPA-n3 | -0.005 | 0.012 | 0.7 | 1 | 34 |
| RBC | DTA | 0.01 | 0.018 | 0.562 | 1 | 34 |
| Plasma | DHA | 0.104 | 0.079 | 0.186 | 1 | 34 |

PUFA, polyunsaturated fatty acid; RBC, red blood cell; ALA, α-linolenic acid; LA, linoleic acid; GLA, γ-linolenic acid; DGLA, dihomo-γ-linolenic acid; AA, arachidonic acid; DPA-n3, docosapentaenoic acid; DTA, docosatetraenoic acid; DHA, docosahexaenoic acid; β, causal effect size; SE, standard error; IVW_MRE, inverse-variance weighted random-effects model; Egger, MR-Egger; nsnps, number of SNPs retained for this analysis.

**Supplementary Table 17.** Reverse Mendelian randomization estimates of associations of genetically predicted COVID-19 susceptibility with polyunsaturated fatty acids based on the release 5 HGI C2 (COVID-19 SNP *P* < 5e-8).

|  |  | **IVW_MRE** | | | **MR-Egger** | | | **MR-Egger intercept** | | **Heterogeneity test** | | **Weighted median** | | |  |  |
| --- | --- | --- | --- | --- | --- | --- | --- | --- | --- | --- | --- | --- | --- | --- | --- | --- |
| **Group name** | **PUFA** | **β** | **SE** | ***P*** | **β** | **SE** | ***P*** | **β** | ***P*** | ***P*_IVW** | ***P*_Egger** | **β** | **SE** | ***P*** | **nsnps** | ***F*-statistic** |
| Plasma | ALA | 0.009 | 0.004 | 0.017 | 0.024 | 0.041 | 0.660 | -0.001 | 0.770 | 0.794 | 0.572 | 0.008 | 0.009 | 0.393 | 3 | 139 |
| RBC | ALA | 0.038 | 0.017 | 0.026 | 0.133 | 0.150 | 0.425 | -0.008 | 0.548 | 0.952 | 0.952 | 0.025 | 0.045 | 0.574 | 6 | 319 |
| RBC | LA | -0.004 | 0.013 | 0.772 | 0.001 | 0.062 | 0.992 | -3.71e-04 | 0.944 | 0.424 | 0.295 | 0.012 | 0.017 | 0.495 | 6 | 319 |
| RBC | GLA | -0.026 | 0.057 | 0.656 | -0.347 | 0.219 | 0.188 | 0.026 | 0.204 | 0.299 | 0.437 | -0.021 | 0.068 | 0.760 | 6 | 319 |
| RBC | DGLA | -0.004 | 0.024 | 0.870 | 0.060 | 0.108 | 0.606 | -0.005 | 0.573 | 0.145 | 0.112 | -0.026 | 0.026 | 0.322 | 6 | 319 |
| RBC | AA | 0.003 | 0.005 | 0.527 | -0.022 | 0.037 | 0.577 | 0.002 | 0.513 | 0.876 | 0.863 | 0.004 | 0.011 | 0.735 | 6 | 319 |
| Plasma | DPA-n3 | -0.025 | 0.025 | 0.328 | -0.202 | 0.137 | 0.380 | 0.014 | 0.414 | 0.400 | 0.749 | -0.037 | 0.030 | 0.226 | 3 | 139 |
| RBC | DPA-n3 | -0.001 | 0.010 | 0.888 | 0.023 | 0.060 | 0.723 | -0.002 | 0.700 | 0.814 | 0.722 | 0.006 | 0.018 | 0.756 | 6 | 319 |
| RBC | DTA | -0.005 | 0.018 | 0.771 | -0.122 | 0.089 | 0.243 | 0.009 | 0.249 | 0.616 | 0.785 | -0.003 | 0.026 | 0.904 | 6 | 319 |
| Plasma | DHA | 0.149 | 0.014 | 6.08e-26 | 0.250 | 0.657 | 0.769 | -0.008 | 0.901 | 0.988 | 0.998 | 0.160 | 0.141 | 0.256 | 3 | 139 |

PUFA, polyunsaturated fatty acid; RBC, red blood cell; ALA, α-linolenic acid; LA, linoleic acid; GLA, γ-linolenic acid; DGLA, dihomo-γ-linolenic acid; AA, arachidonic acid; DPA-n3, docosapentaenoic acid; DTA, docosatetraenoic acid; DHA, docosahexaenoic acid; β, causal effect size; SE, standard error; IVW_MRE, inverse-variance weighted random-effects model; Egger, MR-Egger; nsnps, number of SNPs retained for this analysis.

**Supplementary Table 18.** Reverse Mendelian randomization estimates of associations of genetically predicted COVID-19 severity with polyunsaturated fatty acids based on the release 5 HGI A2 (COVID-19 SNP *P* < 5e-6).

|  |  | **IVW_MRE** | | | **MR-Egger** | | | **MR-Egger intercept** | | **Heterogeneity test** | | **Weighted median** | | |  |  |
| --- | --- | --- | --- | --- | --- | --- | --- | --- | --- | --- | --- | --- | --- | --- | --- | --- |
| **Group name** | **PUFA** | **β** | **SE** | ***P*** | **β** | **SE** | ***P*** | **β** | ***P*** | ***P*_IVW** | ***P*_Egger** | **β** | **SE** | ***P*** | **nsnps** | ***F*-statistic** |
| Plasma | ALA | 0.002 | 0.003 | 0.568 | -3.09e-04 | 0.011 | 0.979 | 3.44e-04 | 0.868 | 0.302 | 0.216 | 0.002 | 0.003 | 0.583 | 8 | 262 |
| RBC | ALA | 0.010 | 0.006 | 0.099 | 0.007 | 0.017 | 0.696 | 0.001 | 0.833 | 0.653 | 0.605 | 0.014 | 0.010 | 0.150 | 31 | 1078 |
| RBC | LA | -0.004 | 0.002 | 0.057 | -0.006 | 0.006 | 0.360 | 4.00e-04 | 0.775 | 0.722 | 0.679 | -0.005 | 0.004 | 0.187 | 31 | 1078 |
| RBC | GLA | -0.015 | 0.007 | 0.050 | 0.011 | 0.025 | 0.648 | -0.006 | 0.265 | 0.948 | 0.957 | -0.015 | 0.014 | 0.276 | 31 | 1078 |
| RBC | DGLA | -0.002 | 0.003 | 0.459 | -0.004 | 0.009 | 0.651 | 4.09e-04 | 0.836 | 0.666 | 0.618 | -0.004 | 0.005 | 0.422 | 31 | 1078 |
| RBC | AA | 0.002 | 0.001 | 0.139 | 0.003 | 0.004 | 0.538 | -1.56e-04 | 0.865 | 0.907 | 0.883 | 0.003 | 0.002 | 0.292 | 31 | 1078 |
| Plasma | DPA-n3 | -0.002 | 0.011 | 0.822 | -0.016 | 0.045 | 0.736 | 0.002 | 0.768 | 0.075 | 0.049 | 0.008 | 0.012 | 0.533 | 8 | 262 |
| RBC | DPA-n3 | 2.17e-04 | 0.003 | 0.933 | 0.002 | 0.007 | 0.763 | -4.42e-04 | 0.770 | 0.479 | 0.432 | 0.002 | 0.004 | 0.567 | 31 | 1078 |
| RBC | DTA | 0.004 | 0.004 | 0.349 | 0.011 | 0.010 | 0.302 | -0.002 | 0.455 | 0.521 | 0.499 | 0.003 | 0.006 | 0.645 | 31 | 1078 |
| Plasma | DHA | -0.005 | 0.029 | 0.862 | 0.034 | 0.141 | 0.818 | -0.007 | 0.783 | 0.769 | 0.675 | -0.022 | 0.049 | 0.657 | 8 | 262 |

PUFA, polyunsaturated fatty acid; RBC, red blood cell; ALA, α-linolenic acid; LA, linoleic acid; GLA, γ-linolenic acid; DGLA, dihomo-γ-linolenic acid; AA, arachidonic acid; DPA-n3, docosapentaenoic acid; DTA, docosatetraenoic acid; DHA, docosahexaenoic acid; β, causal effect size; SE, standard error; IVW_MRE, inverse-variance weighted random-effects model; Egger, MR-Egger; nsnps, number of SNPs retained for this analysis.

**Supplementary Table 19.** Reverse Mendelian randomization estimates of associations of genetically predicted COVID-19 severity with polyunsaturated fatty acids based on the release 5 HGI B2 (COVID-19 SNP *P* < 5e-6).

|  |  | **IVW_MRE** | | | **MR-Egger** | | | **MR-Egger intercept** | | **Heterogeneity test** | | **Weighted median** | | |  |  |
| --- | --- | --- | --- | --- | --- | --- | --- | --- | --- | --- | --- | --- | --- | --- | --- | --- |
| **Group name** | **PUFA** | **β** | **SE** | ***P*** | **β** | **SE** | ***P*** | **β** | ***P*** | ***P*_IVW** | ***P*_Egger** | **β** | **SE** | ***P*** | **nsnps** | ***F*-statistic** |
| Plasma | ALA | 0.002 | 0.003 | 0.458 | -0.006 | 0.014 | 0.688 | 0.001 | 0.556 | 0.190 | 0.160 | 0.002 | 0.004 | 0.613 | 13 | 350 |
| RBC | ALA | 0.006 | 0.008 | 0.514 | 0.010 | 0.022 | 0.653 | -0.001 | 0.823 | 0.832 | 0.794 | 0.018 | 0.015 | 0.215 | 27 | 960 |
| RBC | LA | -0.002 | 0.003 | 0.560 | -0.012 | 0.008 | 0.175 | 0.002 | 0.193 | 0.948 | 0.967 | -0.007 | 0.005 | 0.194 | 27 | 960 |
| RBC | GLA | 3.35e-04 | 0.012 | 0.977 | 0.027 | 0.032 | 0.399 | -0.005 | 0.352 | 0.900 | 0.903 | -0.004 | 0.022 | 0.867 | 27 | 960 |
| RBC | DGLA | 0.003 | 0.006 | 0.633 | -0.002 | 0.013 | 0.887 | 0.001 | 0.694 | 0.179 | 0.152 | -0.006 | 0.008 | 0.482 | 27 | 960 |
| RBC | AA | -0.002 | 0.003 | 0.442 | 0.005 | 0.006 | 0.360 | -0.001 | 0.162 | 0.216 | 0.266 | 0.001 | 0.004 | 0.813 | 27 | 960 |
| Plasma | DPA-n3 | -0.002 | 0.008 | 0.765 | -0.053 | 0.039 | 0.201 | 0.006 | 0.207 | 0.777 | 0.852 | -0.002 | 0.013 | 0.898 | 13 | 350 |
| RBC | DPA-n3 | 0.004 | 0.005 | 0.403 | 0.001 | 0.010 | 0.961 | 0.001 | 0.723 | 0.108 | 0.089 | 0.005 | 0.006 | 0.456 | 27 | 960 |
| RBC | DTA | -0.002 | 0.006 | 0.684 | 2.81e-05 | 0.013 | 0.998 | -4.08e-04 | 0.840 | 0.497 | 0.443 | -0.003 | 0.009 | 0.718 | 27 | 960 |
| Plasma | DHA | -0.033 | 0.033 | 0.314 | -0.073 | 0.193 | 0.712 | 0.005 | 0.836 | 0.880 | 0.831 | -0.033 | 0.058 | 0.573 | 13 | 350 |

PUFA, polyunsaturated fatty acid; RBC, red blood cell; ALA, α-linolenic acid; LA, linoleic acid; GLA, γ-linolenic acid; DGLA, dihomo-γ-linolenic acid; AA, arachidonic acid; DPA-n3, docosapentaenoic acid; DTA, docosatetraenoic acid; DHA, docosahexaenoic acid; β, causal effect size; SE, standard error; IVW_MRE, inverse-variance weighted random-effects model; Egger, MR-Egger; nsnps, number of SNPs retained for this analysis.**Supplementary Table 20.** Reverse Mendelian randomization estimates of associations of genetically predicted COVID-19 severity with polyunsaturated fatty acids based on the release 5 HGI B1 (COVID-19 SNP *P* < 5e-6).

|  |  | **IVW_MRE** | | | **MR-Egger** | | | **MR-Egger intercept** | | **Heterogeneity test** | | **Weighted median** | | |  |  |
| --- | --- | --- | --- | --- | --- | --- | --- | --- | --- | --- | --- | --- | --- | --- | --- | --- |
| **Group name** | **PUFA** | **β** | **SE** | ***P*** | **β** | **SE** | ***P*** | **β** | ***P*** | ***P*_IVW** | ***P*_Egger** | **β** | **SE** | ***P*** | **nsnps** | ***F*-statistic** |
| Plasma | ALA | 0.003 | 0.002 | 0.162 | -0.004 | 0.014 | 0.810 | 0.002 | 0.679 | 0.674 | 0.486 | 0.003 | 0.004 | 0.440 | 3 | 81 |
| RBC | ALA | 0.009 | 0.014 | 0.529 | 0.046 | 0.038 | 0.244 | -0.011 | 0.306 | 0.068 | 0.080 | 1.97e-04 | 0.016 | 0.990 | 13 | 317 |
| RBC | LA | -0.001 | 0.004 | 0.891 | -0.010 | 0.011 | 0.404 | 0.003 | 0.397 | 0.451 | 0.431 | -0.002 | 0.006 | 0.698 | 13 | 317 |
| RBC | GLA | 0.025 | 0.015 | 0.097 | 0.032 | 0.043 | 0.467 | -0.002 | 0.850 | 0.598 | 0.516 | 0.022 | 0.022 | 0.299 | 13 | 317 |
| RBC | DGLA | -0.010 | 0.004 | 0.014 | -0.017 | 0.016 | 0.293 | 0.002 | 0.610 | 0.947 | 0.929 | -0.006 | 0.007 | 0.395 | 13 | 317 |
| RBC | AA | -0.001 | 0.003 | 0.753 | -0.005 | 0.009 | 0.629 | 0.001 | 0.687 | 0.091 | 0.068 | -0.001 | 0.004 | 0.744 | 13 | 317 |
| Plasma | DPA-n3 | -0.004 | 0.007 | 0.566 | -0.015 | 0.047 | 0.809 | 0.002 | 0.857 | 0.696 | 0.412 | -0.002 | 0.014 | 0.856 | 3 | 81 |
| RBC | DPA-n3 | 0.005 | 0.003 | 0.147 | -0.001 | 0.012 | 0.948 | 0.002 | 0.615 | 0.866 | 0.831 | 0.005 | 0.006 | 0.388 | 13 | 317 |
| RBC | DTA | -0.009 | 0.009 | 0.340 | 0.004 | 0.025 | 0.862 | -0.004 | 0.582 | 0.035 | 0.028 | -2.75e-05 | 0.010 | 0.998 | 13 | 317 |
| Plasma | DHA | 0.048 | 0.051 | 0.345 | 0.210 | 0.237 | 0.539 | -0.035 | 0.609 | 0.482 | 0.327 | 0.050 | 0.066 | 0.453 | 3 | 81 |

PUFA, polyunsaturated fatty acid; RBC, red blood cell; ALA, α-linolenic acid; LA, linoleic acid; GLA, γ-linolenic acid; DGLA, dihomo-γ-linolenic acid; AA, arachidonic acid; DPA-n3, docosapentaenoic acid; DTA, docosatetraenoic acid; DHA, docosahexaenoic acid; β, causal effect size; SE, standard error; IVW_MRE, inverse-variance weighted random-effects model; Egger, MR-Egger; nsnps, number of SNPs retained for this analysis.**Supplementary Table 21.** Reverse Mendelian randomization estimates of associations of genetically predicted COVID-19 susceptibility with polyunsaturated fatty acids based on the release 5 HGI C2 (COVID-19 SNP *P* < 5e-6).

|  |  | **IVW_MRE** | | | **MR-Egger** | | | **MR-Egger intercept** | | **Heterogeneity test** | | **Weighted median** | | |  |  |
| --- | --- | --- | --- | --- | --- | --- | --- | --- | --- | --- | --- | --- | --- | --- | --- | --- |
| **Group name** | **PUFA** | **β** | **SE** | ***P*** | **β** | **SE** | ***P*** | **β** | ***P*** | ***P*_IVW** | ***P*_Egger** | **β** | **SE** | ***P*** | **nsnps** | ***F*-statistic** |
| Plasma | ALA | -0.003 | 0.009 | 0.759 | 0.046 | 0.037 | 0.262 | -0.004 | 0.227 | 0.060 | 0.108 | 0.004 | 0.009 | 0.634 | 8 | 268 |
| RBC | ALA | 0.059 | 0.024 | 0.015 | 0.084 | 0.087 | 0.343 | -0.002 | 0.760 | 0.304 | 0.261 | 0.053 | 0.032 | 0.103 | 26 | 792 |
| RBC | LA | -0.004 | 0.007 | 0.572 | -0.041 | 0.030 | 0.185 | 0.003 | 0.212 | 0.863 | 0.894 | 0.001 | 0.012 | 0.952 | 26 | 792 |
| RBC | GLA | -0.008 | 0.034 | 0.826 | -0.116 | 0.122 | 0.352 | 0.008 | 0.365 | 0.377 | 0.370 | -0.047 | 0.048 | 0.323 | 26 | 792 |
| RBC | DGLA | -0.011 | 0.015 | 0.451 | -0.057 | 0.053 | 0.293 | 0.003 | 0.378 | 0.044 | 0.044 | -0.003 | 0.019 | 0.868 | 26 | 792 |
| RBC | AA | 1.24e-04 | 0.005 | 0.980 | 0.001 | 0.020 | 0.952 | -7.98e-05 | 0.955 | 0.761 | 0.712 | 0.001 | 0.008 | 0.910 | 26 | 792 |
| Plasma | DPA-n3 | -0.030 | 0.030 | 0.310 | -0.056 | 0.144 | 0.712 | 0.002 | 0.862 | 0.054 | 0.032 | -0.033 | 0.028 | 0.236 | 8 | 268 |
| RBC | DPA-n3 | 4.15e-04 | 0.009 | 0.963 | 0.033 | 0.032 | 0.316 | -0.002 | 0.303 | 0.511 | 0.518 | 0.004 | 0.013 | 0.744 | 26 | 792 |
| RBC | DTA | 0.011 | 0.013 | 0.378 | -0.037 | 0.048 | 0.446 | 0.004 | 0.302 | 0.573 | 0.582 | 0.001 | 0.019 | 0.945 | 26 | 792 |
| Plasma | DHA | -0.074 | 0.160 | 0.645 | 0.429 | 0.739 | 0.583 | -0.036 | 0.511 | 0.014 | 0.012 | 0.132 | 0.127 | 0.300 | 8 | 268 |

PUFA, polyunsaturated fatty acid; RBC, red blood cell; ALA, α-linolenic acid; LA, linoleic acid; GLA, γ-linolenic acid; DGLA, dihomo-γ-linolenic acid; AA, arachidonic acid; DPA-n3, docosapentaenoic acid; DTA, docosatetraenoic acid; DHA, docosahexaenoic acid; β, causal effect size; SE, standard error; IVW_MRE, inverse-variance weighted random-effects model; Egger, MR-Egger; nsnps, number of SNPs retained for this analysis.**Supplementary Table 22.** Reverse Mendelian randomization estimates of associations of genetically predicted COVID-19 severity with polyunsaturated fatty acids based on the release 4 HGI A2 (COVID-19 SNP *P* < 5e-8).

|  |  | **IVW_MRE** | | | **MR-Egger** | | | **MR-Egger intercept** | | **Heterogeneity test** | | **Weighted median** | | |  |  |
| --- | --- | --- | --- | --- | --- | --- | --- | --- | --- | --- | --- | --- | --- | --- | --- | --- |
| **Group name** | **PUFA** | **β** | **SE** | ***P*** | **β** | **SE** | ***P*** | **β** | ***P*** | ***P*_IVW** | ***P*_Egger** | **β** | **SE** | ***P*** | **nsnps** | ***F*-statistic** |
| Plasma | ALA | 0.001 | 0.006 | 0.874 | 0.125 | 0.071 | 0.331 | -0.024 | 0.332 | 0.134 | 0.318 | -0.002 | 0.005 | 0.677 | 3 | 135 |
| RBC | ALA | 0.008 | 0.009 | 0.383 | 0.014 | 0.022 | 0.558 | -0.002 | 0.781 | 0.449 | 0.339 | 0.011 | 0.012 | 0.376 | 7 | 487 |
| RBC | LA | -0.003 | 0.003 | 0.406 | -0.011 | 0.008 | 0.226 | 0.003 | 0.301 | 0.514 | 0.564 | -0.005 | 0.004 | 0.200 | 7 | 487 |
| RBC | GLA | -0.006 | 0.006 | 0.350 | 0.014 | 0.030 | 0.650 | -0.006 | 0.483 | 0.976 | 0.985 | -0.009 | 0.016 | 0.592 | 7 | 487 |
| RBC | DGLA | -0.003 | 0.004 | 0.338 | -0.009 | 0.011 | 0.455 | 0.002 | 0.603 | 0.785 | 0.719 | -0.004 | 0.006 | 0.500 | 7 | 487 |
| RBC | AA | 1.46e-04 | 0.001 | 0.918 | 0.004 | 0.005 | 0.479 | -0.001 | 0.446 | 0.887 | 0.895 | 0.001 | 0.003 | 0.792 | 7 | 487 |
| Plasma | DPA-n3 | 0.009 | 0.018 | 0.626 | -0.293 | 0.296 | 0.503 | 0.058 | 0.493 | 0.230 | 0.231 | 0.012 | 0.015 | 0.432 | 3 | 135 |
| RBC | DPA-n3 | 0.005 | 0.002 | 0.068 | 0.001 | 0.008 | 0.894 | 0.001 | 0.663 | 0.851 | 0.785 | 0.003 | 0.005 | 0.462 | 7 | 487 |
| RBC | DTA | 0.004 | 0.004 | 0.340 | -0.007 | 0.012 | 0.587 | 0.004 | 0.346 | 0.699 | 0.738 | -0.001 | 0.007 | 0.847 | 7 | 487 |
| Plasma | DHA | -0.091 | 0.066 | 0.166 | -0.032 | 1.141 | 0.982 | -0.012 | 0.967 | 0.380 | 0.165 | -0.080 | 0.070 | 0.254 | 3 | 135 |

PUFA, polyunsaturated fatty acid; RBC, red blood cell; ALA, α-linolenic acid; LA, linoleic acid; GLA, γ-linolenic acid; DGLA, dihomo-γ-linolenic acid; AA, arachidonic acid; DPA-n3, docosapentaenoic acid; DTA, docosatetraenoic acid; DHA, docosahexaenoic acid; β, causal effect size; SE, standard error; IVW_MRE, inverse-variance weighted random-effects model; Egger, MR-Egger; nsnps, number of SNPs retained for this analysis.

**Supplementary Table 23.** Reverse Mendelian randomization estimates of associations of genetically predicted COVID-19 severity with polyunsaturated fatty acids based on the release 4 HGI B2 (COVID-19 SNP *P* < 5e-8).

|  |  | **IVW_MRE** | | | **MR-Egger** | | | **Wald ratio** | | | **MR-Egger intercept** | | **Heterogeneity test** | | **Weighted median** | | |  |  |
| --- | --- | --- | --- | --- | --- | --- | --- | --- | --- | --- | --- | --- | --- | --- | --- | --- | --- | --- | --- |
| **Group name** | **PUFA** | **β** | **SE** | **P** | **β** | **SE** | **P** | **β** | **SE** | **P** | **β** | **P** | **P_IVW** | **P_Egger** | **β** | **SE** | **P** | **nsnps** | **F-statistic** |
| Plasma | ALA | - | - | - | - | - | - | 0.03 | 0.015 | 0.049 | - | - | - | - | - | - | - | 1 | 61 |
| RBC | ALA | 0.005 | 0.013 | 0.688 | 0.022 | 0.03 | 0.501 | - | - | - | -0.005 | 0.558 | 0.197 | 0.155 | 0.013 | 0.014 | 0.333 | 6 | 462 |
| RBC | LA | -0.003 | 0.004 | 0.366 | -0.011 | 0.009 | 0.29 | - | - | - | 0.002 | 0.402 | 0.498 | 0.48 | -0.006 | 0.005 | 0.224 | 6 | 462 |
| RBC | GLA | -0.01 | 0.009 | 0.279 | 0.018 | 0.034 | 0.625 | - | - | - | -0.008 | 0.409 | 0.891 | 0.934 | -0.012 | 0.019 | 0.542 | 6 | 462 |
| RBC | DGLA | -0.005 | 0.005 | 0.349 | -0.004 | 0.012 | 0.737 | - | - | - | -8.21E-05 | 0.98 | 0.548 | 0.405 | -0.004 | 0.007 | 0.546 | 6 | 462 |
| RBC | AA | 0.001 | 0.002 | 0.818 | 0.002 | 0.006 | 0.734 | - | - | - | -4.40E-04 | 0.774 | 0.599 | 0.468 | 3.89E-04 | 0.003 | 0.905 | 6 | 462 |
| Plasma | DPA-n3 | - | - | - | - | - | - | -0.009 | 0.055 | 0.864 | - | - | - | - | - | - | - | 1 | 61 |
| RBC | DPA-n3 | 0.005 | 0.003 | 0.079 | 0.003 | 0.009 | 0.792 | - | - | - | 0.001 | 0.767 | 0.786 | 0.675 | 0.004 | 0.005 | 0.429 | 6 | 462 |
| RBC | DTA | 0.005 | 0.006 | 0.422 | -0.013 | 0.014 | 0.417 | - | - | - | 0.005 | 0.23 | 0.464 | 0.624 | -0.002 | 0.008 | 0.826 | 6 | 462 |
| Plasma | DHA | - | - | - | - | - | - | -0.027 | 0.155 | 0.862 | - | - | - | - | - | - | - | 1 | 61 |

PUFA, polyunsaturated fatty acid; RBC, red blood cell; ALA, α-linolenic acid; LA, linoleic acid; GLA, γ-linolenic acid; DGLA, dihomo-γ-linolenic acid; AA, arachidonic acid; DPA-n3, docosapentaenoic acid; DTA, docosatetraenoic acid; DHA, docosahexaenoic acid; β, causal effect size; SE, standard error; IVW_MRE, inverse-variance weighted random-effects model; Egger, MR-Egger; nsnps, number of SNPs retained for this analysis.**Supplementary Table 24.** Reverse Mendelian randomization estimates of associations of genetically predicted COVID-19 severity with polyunsaturated fatty acids based on the release 4 HGI B1 (COVID-19 SNP *P* < 5e-8).

|  |  | **Wald ratio** | | |  |  |
| --- | --- | --- | --- | --- | --- | --- |
| **Group name** | **PUFA** | **β** | **SE** | ***P*** | **nsnps** | ***F*-statistic** |
| RBC | ALA | 0.004 | 0.023 | 0.874 | 1 | 31 |
| RBC | LA | -0.008 | 0.009 | 0.347 | 1 | 31 |
| RBC | GLA | 0.02 | 0.033 | 0.545 | 1 | 31 |
| RBC | DGLA | 0.001 | 0.012 | 0.91 | 1 | 31 |
| RBC | AA | 0.006 | 0.006 | 0.299 | 1 | 31 |
| RBC | DPA-n3 | -0.003 | 0.009 | 0.771 | 1 | 31 |
| RBC | DTA | 0.006 | 0.014 | 0.635 | 1 | 31 |

PUFA, polyunsaturated fatty acid; RBC, red blood cell; ALA, α-linolenic acid; LA, linoleic acid; GLA, γ-linolenic acid; DGLA, dihomo-γ-linolenic acid; AA, arachidonic acid; DPA-n3, docosapentaenoic acid; DTA, docosatetraenoic acid; DHA, docosahexaenoic acid; β, causal effect size; SE, standard error; IVW_MRE, inverse-variance weighted random-effects model; Egger, MR-Egger; nsnps, number of SNPs retained for this analysis.

**Supplementary Table 25.** Reverse Mendelian randomization estimates of associations of genetically predicted COVID-19 susceptibility with polyunsaturated fatty acids based on the release 4 HGI C2 (COVID-19 SNP *P* < 5e-8).

|  |  | **IVW_MRE** | | | **MR-Egger** | | | **Wald ratio** | | | **MR-Egger intercept** | | **Heterogeneity test** | | **Weighted median** | | |  |  |
| --- | --- | --- | --- | --- | --- | --- | --- | --- | --- | --- | --- | --- | --- | --- | --- | --- | --- | --- | --- |
| **Group name** | **PUFA** | **β** | **SE** | **P** | **β** | **SE** | **P** | **β** | **SE** | **P** | **β** | **P** | **P_IVW** | **P_Egger** | **β** | **SE** | **P** | **nsnps** | **F-statistic** |
| Plasma | ALA | - | - | - | - | - | - | 0.066 | 0.034 | 0.049 | - | - | - | - | - | - | - | 1 | 31 |
| RBC | ALA | 0.034 | 0.018 | 0.057 | -0.005 | 0.059 | 0.942 | - | - | - | 0.008 | 0.583 | 0.686 | 0.686 | 0.034 | 0.032 | 0.292 | 3 | 183 |
| RBC | LA | -0.014 | 0.013 | 0.289 | -0.020 | 0.037 | 0.687 | - | - | - | 0.001 | 0.885 | 0.238 | 0.096 | -0.018 | 0.012 | 0.129 | 3 | 183 |
| RBC | GLA | -0.022 | 0.024 | 0.352 | 0.001 | 0.087 | 0.995 | - | - | - | -0.004 | 0.810 | 0.727 | 0.461 | -0.017 | 0.046 | 0.709 | 3 | 183 |
| RBC | DGLA | -0.011 | 0.026 | 0.666 | -0.039 | 0.066 | 0.660 | - | - | - | 0.005 | 0.712 | 0.066 | 0.036 | -0.005 | 0.016 | 0.757 | 3 | 183 |
| RBC | AA | 0.001 | 0.007 | 0.881 | 0.010 | 0.018 | 0.682 | - | - | - | -0.002 | 0.675 | 0.388 | 0.230 | 0.002 | 0.008 | 0.756 | 3 | 183 |
| Plasma | DPA-n3 | - | - | - | - | - | - | -0.021 | 0.121 | 0.864 | - | - | - | - | - | - | - | 1 | 31 |
| RBC | DPA-n3 | 0.012 | 0.009 | 0.176 | -0.002 | 0.024 | 0.941 | - | - | - | 0.003 | 0.623 | 0.582 | 0.427 | 0.011 | 0.012 | 0.380 | 3 | 183 |
| RBC | DTA | -0.005 | 0.039 | 0.891 | -0.055 | 0.096 | 0.670 | - | - | - | 0.009 | 0.660 | 0.007 | 0.007 | 0.002 | 0.018 | 0.901 | 3 | 183 |
| Plasma | DHA | - | - | - | - | - | - | -0.059 | 0.337 | 0.862 | - | - | - | - | - | - | - | 1 | 31 |

PUFA, polyunsaturated fatty acid; RBC, red blood cell; ALA, α-linolenic acid; LA, linoleic acid; GLA, γ-linolenic acid; DGLA, dihomo-γ-linolenic acid; AA, arachidonic acid; DPA-n3, docosapentaenoic acid; DTA, docosatetraenoic acid; DHA, docosahexaenoic acid; β, causal effect size; SE, standard error; IVW_MRE, inverse-variance weighted random-effects model; Egger, MR-Egger; nsnps, number of SNPs retained for this analysis.**Supplementary Table 26.** Reverse Mendelian randomization estimates of associations of genetically predicted COVID-19 severity with polyunsaturated fatty acids based on the release 4 HGI A2 (COVID-19 SNP *P* < 5e-6).

|  |  | **IVW_MRE** | | | **MR-Egger** | | | **MR-Egger intercept** | | **Heterogeneity test** | | **Weighted median** | | |  |  |
| --- | --- | --- | --- | --- | --- | --- | --- | --- | --- | --- | --- | --- | --- | --- | --- | --- |
| **Group name** | **PUFA** | **β** | **SE** | ***P*** | **β** | **SE** | ***P*** | **β** | ***P*** | ***P*_IVW** | ***P*_Egger** | **β** | **SE** | ***P*** | **nsnps** | ***F*-statistic** |
| Plasma | ALA | 4.31e-04 | 0.004 | 0.909 | 0.019 | 0.024 | 0.464 | -0.003 | 0.468 | 0.120 | 0.110 | -0.001 | 0.004 | 0.685 | 6 | 202 |
| RBC | ALA | 0.012 | 0.007 | 0.080 | -0.007 | 0.014 | 0.634 | 0.005 | 0.153 | 0.431 | 0.498 | 0.014 | 0.011 | 0.184 | 25 | 905 |
| RBC | LA | -0.004 | 0.003 | 0.085 | -0.006 | 0.005 | 0.279 | 4.86e-04 | 0.721 | 0.471 | 0.420 | -0.006 | 0.004 | 0.126 | 25 | 905 |
| RBC | GLA | -0.016 | 0.010 | 0.100 | -0.011 | 0.021 | 0.609 | -0.001 | 0.794 | 0.470 | 0.416 | -0.006 | 0.014 | 0.692 | 25 | 905 |
| RBC | DGLA | -0.001 | 0.004 | 0.809 | 1.97e-04 | 0.008 | 0.981 | -3.03e-04 | 0.880 | 0.346 | 0.296 | -0.004 | 0.006 | 0.456 | 25 | 905 |
| RBC | AA | 0.001 | 0.001 | 0.419 | 0.001 | 0.004 | 0.848 | 1.20e-04 | 0.891 | 0.849 | 0.810 | 0.002 | 0.002 | 0.454 | 25 | 905 |
| Plasma | DPA-n3 | 0.007 | 0.009 | 0.446 | -0.016 | 0.064 | 0.816 | 0.004 | 0.732 | 0.404 | 0.294 | 0.008 | 0.011 | 0.467 | 6 | 202 |
| RBC | DPA-n3 | 0.004 | 0.003 | 0.155 | 0.005 | 0.006 | 0.367 | -4.37e-04 | 0.763 | 0.486 | 0.433 | 0.003 | 0.004 | 0.404 | 25 | 905 |
| RBC | DTA | 0.004 | 0.004 | 0.366 | -0.005 | 0.009 | 0.604 | 0.002 | 0.294 | 0.257 | 0.268 | 0.003 | 0.006 | 0.623 | 25 | 905 |
| Plasma | DHA | -0.081 | 0.064 | 0.204 | -0.155 | 0.403 | 0.720 | 0.012 | 0.860 | 0.067 | 0.037 | -0.087 | 0.060 | 0.146 | 6 | 202 |

PUFA, polyunsaturated fatty acid; RBC, red blood cell; ALA, α-linolenic acid; LA, linoleic acid; GLA, γ-linolenic acid; DGLA, dihomo-γ-linolenic acid; AA, arachidonic acid; DPA-n3, docosapentaenoic acid; DTA, docosatetraenoic acid; DHA, docosahexaenoic acid; β, causal effect size; SE, standard error; IVW_MRE, inverse-variance weighted random-effects model; Egger, MR-Egger; nsnps, number of SNPs retained for this analysis.**Supplementary Table 27.** Reverse Mendelian randomization estimates of associations of genetically predicted COVID-19 severity with polyunsaturated fatty acids based on the release 4 HGI B2 (COVID-19 SNP *P* < 5e-6).

|  |  | **IVW_MRE** | | | **MR-Egger** | | | **MR-Egger intercept** | | **Heterogeneity test** | | **Weighted median** | | |  |  |
| --- | --- | --- | --- | --- | --- | --- | --- | --- | --- | --- | --- | --- | --- | --- | --- | --- |
| **Group name** | **PUFA** | **β** | **SE** | ***P*** | **β** | **SE** | ***P*** | **β** | ***P*** | ***P*_IVW** | ***P*_Egger** | **β** | **SE** | ***P*** | **nsnps** | ***F*-statistic** |
| Plasma | ALA | 1.44e-04 | 0.005 | 0.975 | -0.005 | 0.019 | 0.794 | 0.001 | 0.781 | 0.173 | 0.110 | -0.001 | 0.005 | 0.889 | 6 | 180 |
| RBC | ALA | 0.005 | 0.005 | 0.357 | 0.005 | 0.015 | 0.761 | -1.07e-05 | 0.997 | 0.990 | 0.985 | 0.014 | 0.011 | 0.197 | 32 | 1076 |
| RBC | LA | -4.20e-04 | 0.003 | 0.874 | -0.009 | 0.006 | 0.149 | 0.002 | 0.122 | 0.543 | 0.626 | -0.005 | 0.004 | 0.219 | 32 | 1076 |
| RBC | GLA | -0.016 | 0.008 | 0.043 | 0.014 | 0.023 | 0.529 | -0.007 | 0.135 | 0.965 | 0.983 | -0.019 | 0.015 | 0.208 | 32 | 1076 |
| RBC | DGLA | -1.03e-04 | 0.004 | 0.977 | 0.004 | 0.008 | 0.640 | -0.001 | 0.588 | 0.648 | 0.614 | -0.003 | 0.006 | 0.569 | 32 | 1076 |
| RBC | AA | 3.42e-04 | 0.002 | 0.840 | 0.003 | 0.004 | 0.393 | -0.001 | 0.387 | 0.605 | 0.595 | -0.002 | 0.003 | 0.406 | 32 | 1076 |
| Plasma | DPA-n3 | 0.005 | 0.013 | 0.726 | 0.019 | 0.056 | 0.757 | -0.002 | 0.810 | 0.275 | 0.183 | -0.011 | 0.015 | 0.482 | 6 | 180 |
| RBC | DPA-n3 | 0.002 | 0.003 | 0.501 | -2.94e-04 | 0.006 | 0.963 | 5.00e-04 | 0.696 | 0.585 | 0.542 | 0.002 | 0.004 | 0.571 | 32 | 1076 |
| RBC | DTA | -2.57e-04 | 0.004 | 0.952 | 0.002 | 0.009 | 0.814 | -0.001 | 0.767 | 0.477 | 0.430 | -0.002 | 0.006 | 0.701 | 32 | 1076 |
| Plasma | DHA | -0.058 | 0.037 | 0.120 | -0.098 | 0.200 | 0.648 | 0.006 | 0.842 | 0.807 | 0.690 | -0.046 | 0.065 | 0.480 | 6 | 180 |

PUFA, polyunsaturated fatty acid; RBC, red blood cell; ALA, α-linolenic acid; LA, linoleic acid; GLA, γ-linolenic acid; DGLA, dihomo-γ-linolenic acid; AA, arachidonic acid; DPA-n3, docosapentaenoic acid; DTA, docosatetraenoic acid; DHA, docosahexaenoic acid; β, causal effect size; SE, standard error; IVW_MRE, inverse-variance weighted random-effects model; Egger, MR-Egger; nsnps, number of SNPs retained for this analysis.**Supplementary Table 28.** Reverse Mendelian randomization estimates of associations of genetically predicted COVID-19 severity with polyunsaturated fatty acids based on the release 4 HGI B1 (COVID-19 SNP *P* < 5e-6).

|  |  | **IVW_MRE** | | | **MR-Egger** | | | **Wald ratio** | | | **MR-Egger intercept** | | **Heterogeneity test** | | **Weighted median** | | |  |  |
| --- | --- | --- | --- | --- | --- | --- | --- | --- | --- | --- | --- | --- | --- | --- | --- | --- | --- | --- | --- |
| **Group name** | **PUFA** | **β** | **SE** | **P** | **β** | **SE** | **P** | **β** | **SE** | **P** | **β** | **P** | **P_IVW** | **P_Egger** | **β** | **SE** | **P** | **nsnps** | **F-statistic** |
| Plasma | ALA | - | - | - | - | - | - | 0.006 | 0.006 | 0.317 | - | - | - | - | - | - | - | 1 | 22 |
| RBC | ALA | 0.002 | 0.009 | 0.834 | -0.008 | 0.021 | 0.696 | - | - | - | 0.004 | 0.588 | 0.471 | 0.408 | 0.004 | 0.013 | 0.778 | 11 | 262 |
| RBC | LA | 0.001 | 0.003 | 0.807 | -0.001 | 0.008 | 0.886 | - | - | - | 0.001 | 0.784 | 0.632 | 0.546 | -0.001 | 0.005 | 0.771 | 11 | 262 |
| RBC | GLA | 0.011 | 0.014 | 0.420 | 0.072 | 0.030 | 0.037 | - | - | - | -0.026 | 0.045 | 0.406 | 0.837 | 0.031 | 0.019 | 0.099 | 11 | 262 |
| RBC | DGLA | 0.004 | 0.006 | 0.492 | 0.001 | 0.013 | 0.960 | - | - | - | 0.001 | 0.778 | 0.180 | 0.133 | 0.001 | 0.006 | 0.842 | 11 | 262 |
| RBC | AA | 0.001 | 0.002 | 0.470 | 0.004 | 0.005 | 0.428 | - | - | - | -0.001 | 0.528 | 0.863 | 0.837 | 0.003 | 0.003 | 0.359 | 11 | 262 |
| Plasma | DPA-n3 | - | - | - | - | - | - | 0.001 | 0.021 | 0.976 | - | - | - | - | - | - | - | 1 | 22 |
| RBC | DPA-n3 | -0.003 | 0.004 | 0.409 | -0.007 | 0.008 | 0.408 | - | - | - | 0.002 | 0.588 | 0.510 | 0.445 | -0.002 | 0.005 | 0.716 | 11 | 262 |
| RBC | DTA | 0.009 | 0.004 | 0.016 | 0.019 | 0.012 | 0.152 | - | - | - | -0.004 | 0.378 | 0.929 | 0.941 | 0.004 | 0.007 | 0.589 | 11 | 262 |
| Plasma | DHA | - | - | - | - | - | - | 0.109 | 0.095 | 0.249 | - | - | - | - | - | - | - | 1 | 22 |

PUFA, polyunsaturated fatty acid; RBC, red blood cell; ALA, α-linolenic acid; LA, linoleic acid; GLA, γ-linolenic acid; DGLA, dihomo-γ-linolenic acid; AA, arachidonic acid; DPA-n3, docosapentaenoic acid; DTA, docosatetraenoic acid; DHA, docosahexaenoic acid; β, causal effect size; SE, standard error; IVW_MRE, inverse-variance weighted random-effects model; Egger, MR-Egger; nsnps, number of SNPs retained for this analysis.**Supplementary Table 29.** Reverse Mendelian randomization estimates of associations of genetically predicted COVID-19 susceptibility with polyunsaturated fatty acids based on the release 4 HGI C2 (COVID-19 SNP *P* < 5e-6).

|  |  | **IVW_MRE** | | | **MR-Egger** | | | **MR-Egger intercept** | | **Heterogeneity test** | | **Weighted median** | | |  |  |
| --- | --- | --- | --- | --- | --- | --- | --- | --- | --- | --- | --- | --- | --- | --- | --- | --- |
| **Group name** | **PUFA** | **β** | **SE** | ***P*** | **β** | **SE** | ***P*** | **β** | ***P*** | ***P*_IVW** | ***P*_Egger** | **β** | **SE** | ***P*** | **nsnps** | ***F*-statistic** |
| Plasma | ALA | -0.001 | 0.006 | 0.891 | 0.021 | 0.053 | 0.705 | -0.002 | 0.693 | 0.349 | 0.262 | -0.002 | 0.007 | 0.756 | 7 | 172 |
| RBC | ALA | 0.013 | 0.011 | 0.206 | -0.004 | 0.037 | 0.909 | 0.002 | 0.607 | 0.982 | 0.977 | 0.018 | 0.022 | 0.412 | 19 | 556 |
| RBC | LA | 0.001 | 0.006 | 0.881 | -0.014 | 0.014 | 0.328 | 0.002 | 0.252 | 0.476 | 0.504 | -1.91e-04 | 0.009 | 0.983 | 19 | 556 |
| RBC | GLA | -0.030 | 0.015 | 0.043 | -0.015 | 0.055 | 0.781 | -0.002 | 0.776 | 0.990 | 0.985 | -0.015 | 0.032 | 0.638 | 19 | 556 |
| RBC | DGLA | -0.013 | 0.010 | 0.171 | -0.020 | 0.023 | 0.401 | 0.001 | 0.754 | 0.166 | 0.133 | -0.008 | 0.013 | 0.543 | 19 | 556 |
| RBC | AA | -0.003 | 0.005 | 0.518 | 0.001 | 0.011 | 0.894 | -0.001 | 0.658 | 0.183 | 0.152 | -0.002 | 0.006 | 0.733 | 19 | 556 |
| Plasma | DPA-n3 | 0.006 | 0.018 | 0.732 | -0.178 | 0.151 | 0.293 | 0.014 | 0.275 | 0.341 | 0.390 | 0.019 | 0.023 | 0.417 | 7 | 172 |
| RBC | DPA-n3 | 0.008 | 0.007 | 0.286 | 0.005 | 0.017 | 0.759 | 2.77e-04 | 0.883 | 0.212 | 0.168 | 0.005 | 0.010 | 0.587 | 19 | 556 |
| RBC | DTA | -0.003 | 0.012 | 0.818 | -0.019 | 0.029 | 0.515 | 0.002 | 0.538 | 0.050 | 0.043 | -0.004 | 0.014 | 0.769 | 19 | 556 |
| Plasma | DHA | -0.137 | 0.082 | 0.095 | -0.840 | 0.710 | 0.290 | 0.055 | 0.364 | 0.467 | 0.463 | -0.120 | 0.116 | 0.301 | 7 | 172 |

PUFA, polyunsaturated fatty acid; RBC, red blood cell; ALA, α-linolenic acid; LA, linoleic acid; GLA, γ-linolenic acid; DGLA, dihomo-γ-linolenic acid; AA, arachidonic acid; DPA-n3, docosapentaenoic acid; DTA, docosatetraenoic acid; DHA, docosahexaenoic acid; β, causal effect size; SE, standard error; IVW_MRE, inverse-variance weighted random-effects model; Egger, MR-Egger; nsnps, number of SNPs retained for this analysis.

**Supplementary Table 30.** Forward Mendelian randomization estimates of associations of genetically predicted polyunsaturated fatty acids with COVID-19 severity and susceptibility.

| **Release 5 HGI A2 (COVID-19 severity)** | | | | | | | | | | | | | | | | |
| --- | --- | --- | --- | --- | --- | --- | --- | --- | --- | --- | --- | --- | --- | --- | --- | --- |
|  |  | **IVW_MRE** | | | **MR-Egger** | | | **MR-Egger intercept** | | **Heterogeneity test** | | **Weighted median** | | |  |  |
| **PUFA** | **R^2^** | **β** | **SE** | ***P*** | **β** | **SE** | ***P*** | **β** | ***P*** | ***P*_IVW** | ***P*_Egger** | **β** | **SE** | ***P*** | **nsnps** | ***F*-statistic** |
| Omega-3 PUFAs \|\| id:met-c-855 | 0.001 | -0.168 | 0.079 | 0.034 | -0.507 | 0.453 | 0.345 | 0.041 | 0.495 | 0.781 | 0.765 | -0.234 | 0.143 | 0.101 | 5 | 338 |
|  | 0.01 | -0.168 | 0.079 | 0.034 | -0.507 | 0.453 | 0.345 | 0.041 | 0.495 | 0.781 | 0.765 | -0.234 | 0.140 | 0.093 | 5 | 338 |
|  | 0.1 | -0.162 | 0.071 | 0.022 | -0.512 | 0.451 | 0.320 | 0.042 | 0.467 | 0.875 | 0.884 | -0.234 | 0.144 | 0.104 | 6 | 375 |
|  | 0.3 | -0.230 | 0.099 | 0.020 | -0.476 | 0.430 | 0.296 | 0.028 | 0.569 | 0.398 | 0.342 | -0.234 | 0.129 | 0.069 | 11 | 637 |
| DHA \|\| id:met-c-852 | 0.001 | -0.009 | 0.184 | 0.962 | -0.773 | 1.110 | 0.537 | 0.088 | 0.535 | 0.114 | 0.094 | -0.210 | 0.175 | 0.229 | 5 | 268 |
|  | 0.01 | -0.009 | 0.184 | 0.962 | -0.773 | 1.110 | 0.537 | 0.088 | 0.535 | 0.114 | 0.094 | -0.210 | 0.176 | 0.232 | 5 | 268 |
|  | 0.1 | -0.009 | 0.184 | 0.962 | -0.773 | 1.110 | 0.537 | 0.088 | 0.535 | 0.114 | 0.094 | -0.210 | 0.173 | 0.225 | 5 | 268 |
|  | 0.3 | -0.092 | 0.153 | 0.551 | -0.196 | 0.954 | 0.845 | 0.012 | 0.916 | 0.139 | 0.086 | -0.271 | 0.159 | 0.088 | 7 | 371 |
| Omega-6 PUFAs \|\| id:met-c-856 | 0.001 | -0.072 | 0.070 | 0.309 | 0.153 | 0.216 | 0.497 | -0.032 | 0.287 | 0.743 | 0.785 | -0.049 | 0.111 | 0.660 | 11 | 654 |
|  | 0.01 | -0.077 | 0.075 | 0.306 | 0.089 | 0.216 | 0.690 | -0.024 | 0.424 | 0.643 | 0.622 | -0.051 | 0.113 | 0.654 | 12 | 693 |
|  | 0.1 | -0.065 | 0.070 | 0.356 | 0.102 | 0.215 | 0.645 | -0.024 | 0.421 | 0.701 | 0.684 | -0.041 | 0.105 | 0.694 | 13 | 771 |
|  | 0.3 | -0.075 | 0.072 | 0.295 | 0.057 | 0.212 | 0.791 | -0.019 | 0.513 | 0.665 | 0.625 | -0.043 | 0.105 | 0.685 | 14 | 828 |
| LA \|\| id:met-c-893 | 0.001 | -0.042 | 0.059 | 0.479 | 0.044 | 0.172 | 0.803 | -0.013 | 0.593 | 0.786 | 0.745 | -0.012 | 0.094 | 0.897 | 15 | 950 |
|  | 0.01 | -0.042 | 0.059 | 0.479 | 0.044 | 0.172 | 0.803 | -0.013 | 0.593 | 0.786 | 0.745 | -0.012 | 0.096 | 0.899 | 15 | 950 |
|  | 0.1 | -0.014 | 0.054 | 0.789 | 0.130 | 0.179 | 0.480 | -0.022 | 0.396 | 0.877 | 0.877 | -0.006 | 0.093 | 0.949 | 15 | 1014 |
|  | 0.3 | -0.005 | 0.062 | 0.935 | -0.030 | 0.155 | 0.847 | 0.004 | 0.860 | 0.565 | 0.502 | -0.007 | 0.088 | 0.934 | 21 | 1259 |
| **Release 5 HGI B1 (COVID-19 severity)** | | | | | | | | | | | | | | | | |
|  |  | **IVW_MRE** | | | **MR-Egger** | | | **MR-Egger intercept** | | **Heterogeneity test** | | **Weighted median** | | |  |  |
| **PUFA** | **R^2^** | **β** | **SE** | ***P*** | **β** | **SE** | ***P*** | **β** | ***P*** | ***P*_IVW** | ***P*_Egger** | **β** | **SE** | ***P*** | **nsnps** | ***F*-statistic** |
| Omega-3 PUFAs \|\| id:met-c-855 | 0.001 | -0.278 | 0.062 | 8.81e-06 | -0.574 | 0.506 | 0.320 | 0.037 | 0.576 | 0.958 | 0.953 | -0.207 | 0.166 | 0.212 | 6 | 372 |
|  | 0.01 | -0.278 | 0.062 | 8.81e-06 | -0.574 | 0.506 | 0.320 | 0.037 | 0.576 | 0.958 | 0.953 | -0.207 | 0.173 | 0.232 | 6 | 372 |
|  | 0.1 | -0.276 | 0.055 | 5.13e-07 | -0.568 | 0.501 | 0.308 | 0.036 | 0.572 | 0.983 | 0.984 | -0.216 | 0.155 | 0.164 | 7 | 409 |
|  | 0.3 | -0.278 | 0.065 | 1.75e-05 | -0.582 | 0.437 | 0.212 | 0.034 | 0.491 | 0.956 | 0.952 | -0.221 | 0.136 | 0.103 | 12 | 671 |
| DHA \|\| id:met-c-852 | 0.001 | -0.156 | 0.138 | 0.259 | -0.827 | 0.792 | 0.356 | 0.079 | 0.438 | 0.487 | 0.448 | -0.143 | 0.194 | 0.462 | 6 | 306 |
|  | 0.01 | -0.156 | 0.138 | 0.259 | -0.827 | 0.792 | 0.356 | 0.079 | 0.438 | 0.487 | 0.448 | -0.143 | 0.193 | 0.461 | 6 | 306 |
|  | 0.1 | -0.156 | 0.138 | 0.259 | -0.827 | 0.792 | 0.356 | 0.079 | 0.438 | 0.487 | 0.448 | -0.143 | 0.193 | 0.461 | 6 | 306 |
|  | 0.3 | -0.144 | 0.126 | 0.255 | -0.932 | 0.696 | 0.229 | 0.088 | 0.293 | 0.464 | 0.501 | -0.119 | 0.190 | 0.530 | 8 | 408 |
| Omega-6 PUFAs \|\| id:met-c-856 | 0.001 | 0.032 | 0.103 | 0.755 | 0.221 | 0.264 | 0.427 | -0.028 | 0.458 | 0.377 | 0.343 | -0.033 | 0.138 | 0.809 | 10 | 618 |
|  | 0.01 | 0.032 | 0.103 | 0.755 | 0.221 | 0.264 | 0.427 | -0.028 | 0.458 | 0.377 | 0.343 | -0.033 | 0.135 | 0.806 | 10 | 618 |
|  | 0.1 | 0.107 | 0.117 | 0.362 | 0.283 | 0.317 | 0.396 | -0.026 | 0.563 | 0.120 | 0.098 | -0.001 | 0.135 | 0.993 | 11 | 696 |
|  | 0.3 | 0.102 | 0.111 | 0.358 | 0.259 | 0.297 | 0.403 | -0.024 | 0.579 | 0.161 | 0.132 | -0.009 | 0.136 | 0.947 | 12 | 752 |
| LA \|\| id:met-c-893 | 0.001 | 0.097 | 0.090 | 0.278 | 0.346 | 0.207 | 0.122 | -0.039 | 0.211 | 0.322 | 0.380 | 0.126 | 0.121 | 0.298 | 13 | 866 |
|  | 0.01 | 0.097 | 0.090 | 0.278 | 0.346 | 0.207 | 0.122 | -0.039 | 0.211 | 0.322 | 0.380 | 0.126 | 0.116 | 0.278 | 13 | 866 |
|  | 0.1 | 0.141 | 0.101 | 0.163 | 0.397 | 0.254 | 0.147 | -0.039 | 0.297 | 0.113 | 0.130 | 0.153 | 0.122 | 0.210 | 13 | 930 |
|  | 0.3 | 0.159 | 0.086 | 0.065 | 0.186 | 0.209 | 0.385 | -0.004 | 0.889 | 0.122 | 0.093 | 0.168 | 0.110 | 0.126 | 19 | 1175 |
| **Release 5 HGI B2 (COVID-19 severity)** | | | | | | | | | | | | | | | | |
|  |  | **IVW_MRE** | | | **MR-Egger** | | | **MR-Egger intercept** | | **Heterogeneity test** | | **Weighted median** | | |  |  |
| **PUFA** | **R^2^** | **β** | **SE** | ***P*** | **β** | **SE** | ***P*** | **β** | ***P*** | ***P*_IVW** | ***P*_Egger** | **β** | **SE** | ***P*** | **nsnps** | ***F*-statistic** |
| Omega-3 PUFAs \|\| id:met-c-855 | 0.001 | -0.160 | 0.056 | 0.004 | -0.414 | 0.310 | 0.274 | 0.031 | 0.459 | 0.767 | 0.774 | -0.203 | 0.100 | 0.043 | 5 | 338 |
|  | 0.01 | -0.160 | 0.056 | 0.004 | -0.414 | 0.310 | 0.274 | 0.031 | 0.459 | 0.767 | 0.774 | -0.203 | 0.098 | 0.039 | 5 | 338 |
|  | 0.1 | -0.161 | 0.048 | 0.001 | -0.407 | 0.308 | 0.257 | 0.030 | 0.455 | 0.872 | 0.886 | -0.200 | 0.098 | 0.042 | 6 | 375 |
|  | 0.3 | -0.182 | 0.045 | 6.12e-05 | -0.407 | 0.271 | 0.168 | 0.025 | 0.417 | 0.879 | 0.879 | -0.210 | 0.087 | 0.015 | 11 | 637 |
| DHA \|\| id:met-c-852 | 0.001 | -0.077 | 0.110 | 0.485 | -0.475 | 0.692 | 0.542 | 0.046 | 0.600 | 0.206 | 0.151 | -0.224 | 0.118 | 0.057 | 5 | 268 |
|  | 0.01 | -0.077 | 0.110 | 0.485 | -0.475 | 0.692 | 0.542 | 0.046 | 0.600 | 0.206 | 0.151 | -0.224 | 0.119 | 0.060 | 5 | 268 |
|  | 0.1 | -0.077 | 0.110 | 0.485 | -0.475 | 0.692 | 0.542 | 0.046 | 0.600 | 0.206 | 0.151 | -0.224 | 0.113 | 0.047 | 5 | 268 |
|  | 0.3 | -0.090 | 0.083 | 0.282 | -0.401 | 0.505 | 0.463 | 0.034 | 0.558 | 0.370 | 0.304 | -0.220 | 0.101 | 0.030 | 7 | 371 |
| Omega-6 PUFAs \|\| id:met-c-856 | 0.001 | -0.014 | 0.056 | 0.802 | 0.209 | 0.145 | 0.183 | -0.032 | 0.127 | 0.495 | 0.681 | -0.008 | 0.079 | 0.922 | 11 | 654 |
|  | 0.01 | -0.016 | 0.056 | 0.781 | 0.219 | 0.147 | 0.170 | -0.034 | 0.116 | 0.492 | 0.698 | -0.020 | 0.075 | 0.795 | 11 | 661 |
|  | 0.1 | 0.022 | 0.063 | 0.727 | 0.257 | 0.154 | 0.126 | -0.034 | 0.130 | 0.223 | 0.346 | 0.026 | 0.078 | 0.738 | 12 | 740 |
|  | 0.3 | 0.022 | 0.063 | 0.727 | 0.257 | 0.154 | 0.126 | -0.034 | 0.130 | 0.223 | 0.346 | 0.026 | 0.078 | 0.739 | 12 | 740 |
| LA \|\| id:met-c-893 | 0.001 | 0.019 | 0.045 | 0.671 | 0.188 | 0.119 | 0.141 | -0.026 | 0.146 | 0.603 | 0.740 | 0.017 | 0.067 | 0.800 | 13 | 854 |
|  | 0.01 | 0.019 | 0.045 | 0.671 | 0.188 | 0.119 | 0.141 | -0.026 | 0.146 | 0.603 | 0.740 | 0.017 | 0.067 | 0.800 | 13 | 854 |
|  | 0.1 | 0.057 | 0.051 | 0.257 | 0.276 | 0.124 | 0.047 | -0.033 | 0.081 | 0.354 | 0.575 | 0.054 | 0.068 | 0.429 | 13 | 918 |
|  | 0.3 | 0.055 | 0.048 | 0.250 | 0.095 | 0.118 | 0.436 | -0.006 | 0.721 | 0.236 | 0.195 | 0.021 | 0.062 | 0.734 | 19 | 1164 |
| **Release 5 HGI C2 (COVID-19 susceptibility)** | | | | | | | | | | | | | | | | |
|  |  | **IVW_MRE** | | | **MR-Egger** | | | **MR-Egger intercept** | | **Heterogeneity test** | | **Weighted median** | | |  |  |
| **PUFA** | **R^2^** | **β** | **SE** | ***P*** | **β** | **SE** | ***P*** | **β** | ***P*** | ***P*_IVW** | ***P*_Egger** | **β** | **SE** | ***P*** | **nsnps** | ***F*-statistic** |
| Omega-3 PUFAs \|\| id:met-c-855 | 0.001 | 0.01 | 0.043 | 0.818 | -0.149 | 0.163 | 0.428 | 0.019 | 0.387 | 0.389 | 0.38 | -0.018 | 0.05 | 0.717 | 5 | 338 |
|  | 0.01 | 0.01 | 0.043 | 0.818 | -0.149 | 0.163 | 0.428 | 0.019 | 0.387 | 0.389 | 0.38 | -0.018 | 0.049 | 0.711 | 5 | 338 |
|  | 0.1 | 0.003 | 0.039 | 0.934 | -0.133 | 0.159 | 0.45 | 0.017 | 0.425 | 0.482 | 0.448 | -0.02 | 0.048 | 0.675 | 6 | 375 |
|  | 0.3 | -0.02 | 0.029 | 0.49 | -0.031 | 0.139 | 0.826 | 0.001 | 0.934 | 0.603 | 0.509 | -0.023 | 0.042 | 0.592 | 11 | 637 |
| DHA \|\| id:met-c-852 | 0.001 | -0.02 | 0.04 | 0.608 | 0.025 | 0.272 | 0.934 | -0.005 | 0.877 | 0.567 | 0.404 | -0.032 | 0.057 | 0.572 | 5 | 268 |
|  | 0.01 | -0.02 | 0.04 | 0.608 | 0.025 | 0.272 | 0.934 | -0.005 | 0.877 | 0.567 | 0.404 | -0.032 | 0.06 | 0.593 | 5 | 268 |
|  | 0.1 | -0.02 | 0.04 | 0.608 | 0.025 | 0.272 | 0.934 | -0.005 | 0.877 | 0.567 | 0.404 | -0.032 | 0.055 | 0.562 | 5 | 268 |
|  | 0.3 | -0.003 | 0.032 | 0.932 | -0.079 | 0.236 | 0.751 | 0.008 | 0.755 | 0.724 | 0.617 | -0.02 | 0.051 | 0.693 | 7 | 371 |
| Omega-6 PUFAs \|\| id:met-c-856 | 0.001 | 0.032 | 0.027 | 0.229 | 0.091 | 0.073 | 0.245 | -0.009 | 0.401 | 0.622 | 0.607 | 0.047 | 0.041 | 0.257 | 11 | 654 |
|  | 0.01 | 0.034 | 0.027 | 0.206 | 0.091 | 0.074 | 0.25 | -0.008 | 0.422 | 0.602 | 0.578 | 0.049 | 0.041 | 0.23 | 11 | 661 |
|  | 0.1 | 0.041 | 0.026 | 0.11 | 0.098 | 0.074 | 0.213 | -0.008 | 0.421 | 0.627 | 0.605 | 0.068 | 0.039 | 0.085 | 12 | 740 |
|  | 0.3 | 0.041 | 0.026 | 0.11 | 0.098 | 0.074 | 0.213 | -0.008 | 0.421 | 0.627 | 0.605 | 0.068 | 0.039 | 0.087 | 12 | 740 |
| LA \|\| id:met-c-893 | 0.001 | 0.028 | 0.022 | 0.206 | 0.103 | 0.061 | 0.118 | -0.011 | 0.202 | 0.686 | 0.77 | 0.031 | 0.034 | 0.373 | 13 | 854 |
|  | 0.01 | 0.028 | 0.022 | 0.206 | 0.103 | 0.061 | 0.118 | -0.011 | 0.202 | 0.686 | 0.77 | 0.031 | 0.034 | 0.368 | 13 | 854 |
|  | 0.1 | 0.033 | 0.023 | 0.143 | 0.115 | 0.063 | 0.097 | -0.012 | 0.189 | 0.624 | 0.718 | 0.034 | 0.033 | 0.305 | 13 | 918 |
|  | 0.3 | 0.03 | 0.019 | 0.102 | 0.078 | 0.053 | 0.158 | -0.007 | 0.334 | 0.816 | 0.824 | 0.038 | 0.031 | 0.228 | 19 | 1164 |

PUFA, polyunsaturated fatty acid; RBC, red blood cell; ALA, α-linolenic acid; LA, linoleic acid; GLA, γ-linolenic acid; DGLA, dihomo-γ-linolenic acid; AA, arachidonic acid; DPA-n3, docosapentaenoic acid; DTA, docosatetraenoic acid; DHA, docosahexaenoic acid; β, causal effect size; SE, standard error; IVW_MRE, inverse-variance weighted random-effects model; Egger, MR-Egger; nsnps, number of SNPs retained for this analysis.

**Supplementary Table 31.** Related traits of genetic instruments for arachidonic acid and docosapentaenoic acid.

| **PUFA** | **SNP** | **Chr: position (BP)** | **Effect allele** | **Non effect allele** | **Trait** | **Beta** | **SE** | **P-value** |
| --- | --- | --- | --- | --- | --- | --- | --- | --- |
| Red blood cell AA | rs2581624 | chr3:142633869 | C | G | Red blood cell fatty acid levels | NA | NA | 1.00E-10 |
| Red blood cell AA | rs2072114 | chr11:61605215 | A | G | ALA 18:3n3 | -0.015 | 0.0014 | 2.41E-28 |
|  |  |  |  |  | Adrenic acid 22:4n6 | 0.0419 | 0.0026 | 6.04E-59 |
|  |  |  |  |  | Arachidonic acid 20:4n6 | 1.562 | 0.0382 | 2.38e-366 |
|  |  |  |  |  | Cis trans-18:2 | -0.0037 | 6.00E-04 | 3.13E-09 |
|  |  |  |  |  | DPA 22:5n3 | 0.0655 | 0.0039 | 4.45E-64 |
|  |  |  |  |  | Dihomo-gamma-linolenic acid | -0.1536 | 0.0197 | 5.95E-15 |
|  |  |  |  |  | EPA 20:5n3 | 0.0746 | 0.0067 | 1.42E-28 |
|  |  |  |  |  | Gamma-linolenic acid 18:3n6 | 0.0152 | 0.0013 | 2.34E-31 |
|  |  |  |  |  | Linoleic acid 18:2n6 | -1.556 | 0.0616 | 6.13E-141 |
|  |  |  |  |  | Oleic acid 18:1n9 | -0.2212 | 0.0278 | 1.80E-15 |
|  |  |  |  |  | Stearic acid 18:0 | 0.1672 | 0.0265 | 2.58E-10 |
|  |  |  |  |  | High density lipoprotein | 0.0399 | 0.0048 | 1.44E-16 |
|  |  |  |  |  | Low density lipoprotein | 0.0454 | 0.0052 | 2.47E-17 |
|  |  |  |  |  | Total cholesterol | 0.045 | 0.005 | 2.35E-18 |
|  |  |  |  |  | Triglycerides | -0.0473 | 0.0048 | 5.25E-20 |
|  |  |  |  |  | Fasting blood glucose | NA | NA | 1.10E-08 |
|  |  |  |  |  | HDL cholesterol | NA | NA | 1.23E-14 |
|  |  |  |  |  | LDL cholesterol | NA | NA | 2.47E-09 |
|  |  |  |  |  | Plasma alpha linolenic acid levels | NA | NA | 2.41E-28 |
|  |  |  |  |  | Plasma docosapentaenoic acid levels | NA | NA | 4.45E-64 |
|  |  |  |  |  | Plasma eicosapentaenoic acid levels | NA | NA | 1.42E-28 |
|  |  |  |  |  | Plasma oleic acid | NA | NA | 1.80E-15 |
|  |  |  |  |  | Plasma stearic acid | NA | NA | 2.58E-10 |
|  |  |  |  |  | Heart rate | -0.4574 | 0.0703 | 6.26E-10 |
|  |  |  |  |  | Fasting glucose | 0.023 | 0.003 | 1.60E-14 |
|  |  |  |  |  | Trans fatty acid levels | 0.0037 | 0.0006238 | 3.00E-09 |
|  |  |  |  |  | Pulse rate | -0.03384 | 0.003803 | 5.73E-19 |
| Red blood cell AA | rs2269928 | chr11:61537529 | T | G | Mean platelet volume | 0.02741 | 0.004426 | 5.92E-10 |
|  |  |  |  |  | Red blood cell count | -0.02437 | 0.004356 | 2.20E-08 |
|  |  |  |  |  | Red cell distribution width | 0.03547 | 0.004334 | 2.75E-16 |
| Plasma AA | rs174547 | chr11:61570783 | C | T | Eosinophil count | -0.02407 | 0.003741 | 1.24E-10 |
| Plasma DPA-n3 |  |  |  |  | Granulocyte count | -0.02593 | 0.003767 | 5.82E-12 |
|  |  |  |  |  | Granulocyte percentage of myeloid white cells | -0.03025 | 0.003749 | 7.06E-16 |
|  |  |  |  |  | Hematocrit | 0.02423 | 0.003705 | 6.16E-11 |
|  |  |  |  |  | Hemoglobin concentration | 0.0275 | 0.003721 | 1.47E-13 |
|  |  |  |  |  | Mean corpuscular volume | -0.02382 | 0.003704 | 1.26E-10 |
|  |  |  |  |  | Mean platelet volume | -0.03889 | 0.003792 | 1.14E-24 |
|  |  |  |  |  | Monocyte percentage of white cells | 0.0283 | 0.003739 | 3.77E-14 |
|  |  |  |  |  | Myeloid white cell count | -0.02348 | 0.003778 | 5.15E-10 |
|  |  |  |  |  | Neutrophil count | -0.02351 | 0.003758 | 3.94E-10 |
|  |  |  |  |  | Platelet count | 0.03721 | 0.003824 | 2.21E-22 |
|  |  |  |  |  | Plateletcrit | 0.02208 | 0.003837 | 8.64E-09 |
|  |  |  |  |  | Red blood cell count | 0.03451 | 0.003732 | 2.29E-20 |
|  |  |  |  |  | Red cell distribution width | -0.04908 | 0.003713 | 6.77E-40 |
|  |  |  |  |  | Reticulocyte count | 0.02155 | 0.00377 | 1.09E-08 |
|  |  |  |  |  | Sum basophil neutrophil counts | -0.02375 | 0.003765 | 2.81E-10 |
|  |  |  |  |  | Sum eosinophil basophil counts | -0.02255 | 0.003745 | 1.74E-09 |
|  |  |  |  |  | Sum neutrophil eosinophil counts | -0.02578 | 0.003761 | 7.15E-12 |
|  |  |  |  |  | White blood cell count | -0.0231 | 0.003761 | 8.16E-10 |
|  |  |  |  |  | ALA 18:3n3 | 0.0159 | 9.00E-04 | 3.47E-64 |
|  |  |  |  |  | Adrenic acid 22:4n6 | -0.0483 | 0.0019 | 6.26E-140 |
|  |  |  |  |  | Arachidonic acid 20:4n6 | -1.691 | 0.0253 | 3.30e-971 |
|  |  |  |  |  | Cis trans-18:2 | 0.0032 | 4.00E-04 | 6.17E-14 |
|  |  |  |  |  | DPA 22:5n3 | -0.0746 | 0.0028 | 3.79E-154 |
|  |  |  |  |  | Dihomo-gamma-linolenic acid | 0.355 | 0.0136 | 2.63E-151 |
|  |  |  |  |  | EPA 20:5n3 | -0.082 | 0.0051 | 1.83E-57 |
|  |  |  |  |  | Gamma-linolenic acid 18:3n6 | -0.0156 | 9.00E-04 | 2.29E-72 |
|  |  |  |  |  | Linoleic acid 18:2n6 | 1.474 | 0.0417 | 4.98E-274 |
|  |  |  |  |  | Oleic acid 18:1n9 | 0.2282 | 0.0194 | 5.57E-32 |
|  |  |  |  |  | Palmitoleic acid 16:1n7 | 0.023 | 0.0033 | 2.97E-12 |
|  |  |  |  |  | Stearic acid 18:0 | -0.1773 | 0.0193 | 4.42E-20 |
|  |  |  |  |  | Height | -0.016 | 0.0028 | 2.10E-08 |
|  |  |  |  |  | High density lipoprotein | -0.0389 | 0.0035 | 4.05E-27 |
|  |  |  |  |  | Low density lipoprotein | -0.0505 | 0.0038 | 7.99E-38 |
|  |  |  |  |  | Total cholesterol | -0.0472 | 0.0037 | 1.35E-35 |
|  |  |  |  |  | Triglycerides | 0.0469 | 0.0035 | 1.04E-40 |
|  |  |  |  |  | FADS1 gene expression in human liver | NA | NA | 5.00E-35 |
|  |  |  |  |  | FADS3 gene expression in human liver | NA | NA | 1.00E-08 |
|  |  |  |  |  | Fasting blood glucose | NA | NA | 1.72E-08 |
|  |  |  |  |  | HDL cholesterol | NA | NA | 2.00E-12 |
|  |  |  |  |  | LDL cholesterol | NA | NA | 1.00E-19 |
|  |  |  |  |  | Plasma alpha linolenic acid levels | NA | NA | 7.00E-84 |
|  |  |  |  |  | Plasma docosapentaenoic acid levels | NA | NA | 3.79E-154 |
|  |  |  |  |  | Plasma eicosapentaenoic acid levels | NA | NA | 1.83E-57 |
|  |  |  |  |  | Plasma oleic acid | NA | NA | 5.57E-32 |
|  |  |  |  |  | Plasma palmitoleic acid | NA | NA | 2.97E-12 |
|  |  |  |  |  | Plasma stearic acid | NA | NA | 4.42E-20 |
|  |  |  |  |  | RR interval | NA | NA | 8.20E-10 |
|  |  |  |  |  | Heart rate | 0.3278 | 0.0538 | 6.99E-09 |
|  |  |  |  |  | Fasting glucose | -0.019 | 0.0022 | 1.33E-17 |
|  |  |  |  |  | Age related disease endophenotypes | NA | NA | 1.00E-29 |
|  |  |  |  |  | Age related diseases mortality and associated endophenotypes | NA | NA | 7.00E-25 |
|  |  |  |  |  | Glycerophospholipid levels | -0.1652 | 0.00585 | 2.00E-175 |
|  |  |  |  |  | Lipid metabolism phenotypes | NA | NA | 8.00E-262 |
|  |  |  |  |  | Metabolic traits | 0.178 | 0.007783 | 9.00E-116 |
|  |  |  |  |  | Metabolite levels | 36.3 | 1.273 | 7.00E-179 |
|  |  |  |  |  | Metabolite levels lipid measures | 0.4 | 0.04062 | 7.00E-23 |
|  |  |  |  |  | Phospholipid levels plasma | -0.02 | 0.001182 | 3.00E-64 |
|  |  |  |  |  | Plasma omega 6 polyunsaturated fatty acid levels arachidonic acid | 1.69 | NA | 0 |
|  |  |  |  |  | Plasma omega 6 polyunsaturated fatty acid levels gamma linolenic acid | -0.02 | 0.001111 | 2.00E-72 |
|  |  |  |  |  | Plasma omega 6 polyunsaturated fatty acid levels linoleic acid | -0.048 | 0.00578 | 1.00E-16 |
|  |  |  |  |  | Resting heart rate | 6.2 | 1.034 | 2.00E-09 |
|  |  |  |  |  | Sphingolipid levels | -0.0295 | 0.004508 | 6.00E-11 |
|  |  |  |  |  | Trans fatty acid levels | -0.0032 | 0.0004262 | 6.00E-14 |
|  |  |  |  |  | Asthma | -0.005033 | 0.0008184 | 7.77E-10 |
|  |  |  |  |  | Average weekly champagne plus white wine intake | 0.01429 | 0.002567 | 2.62E-08 |
|  |  |  |  |  | Nap during day | 0.009819 | 0.001514 | 8.91E-11 |
|  |  |  |  |  | Pulse rate | 0.03383 | 0.002632 | 8.59E-38 |
|  |  |  |  |  | Self-reported asthma | -0.005349 | 0.0008199 | 6.86E-11 |
|  |  |  |  |  | Cholesterol hdl | NA | NA | 2.00E-12 |
|  |  |  |  |  | Electrocardiography | NA | NA | 7.00E-179 |
|  |  |  |  |  | Lipid metabolism | NA | NA | 8.00E-262 |
|  |  |  |  |  | Metabolism | NA | NA | 9.00E-116 |
|  |  |  |  |  | Phospholipids | NA | NA | 3.00E-64 |
| Plasma AA | rs16966952 | chr16:15135943 | A | G | Arm fat-free mass left | -0.009646 | 0.001691 | 1.17E-08 |
|  |  |  |  |  | Arm fat-free mass right | -0.009821 | 0.001658 | 3.13E-09 |
|  |  |  |  |  | Arm predicted mass left | -0.009541 | 0.001685 | 1.49E-08 |
|  |  |  |  |  | Arm predicted mass right | -0.009723 | 0.001652 | 3.94E-09 |
|  |  |  |  |  | Basal metabolic rate | -0.01142 | 0.001764 | 9.52E-11 |
|  |  |  |  |  | Comparative height size at age 10 | -0.01001 | 0.001816 | 3.60E-08 |
|  |  |  |  |  | Leg fat-free mass left | -0.0111 | 0.001752 | 2.35E-10 |
|  |  |  |  |  | Leg fat-free mass right | -0.01103 | 0.001751 | 2.98E-10 |
|  |  |  |  |  | Leg predicted mass left | -0.01099 | 0.00174 | 2.62E-10 |
|  |  |  |  |  | Leg predicted mass right | -0.0109 | 0.001739 | 3.72E-10 |
|  |  |  |  |  | Sitting height | -0.01451 | 0.002056 | 1.71E-12 |
|  |  |  |  |  | Trunk fat-free mass | -0.01096 | 0.001676 | 6.15E-11 |
|  |  |  |  |  | Trunk predicted mass | -0.0109 | 0.00167 | 6.78E-11 |
|  |  |  |  |  | Weight | -0.01282 | 0.002325 | 3.50E-08 |
|  |  |  |  |  | Whole body fat-free mass | -0.01113 | 0.001682 | 3.67E-11 |
|  |  |  |  |  | Whole body water mass | -0.01128 | 0.001685 | 2.13E-11 |
|  |  |  |  |  | Whole body fat-free mass | -0.0197 | 0.00158 | 1.06E-35 |
|  |  |  |  |  | Whole body water mass | -0.01995 | 0.001582 | 1.93E-36 |
| Red blood cell DPA-n3 | rs8523 | chr6:10981053 | G | A | DHA 22:6n3 | 0.1141 | 0.0151 | 3.94E-14 |
|  |  |  |  |  | DPA 22:5n3 | -0.0397 | 0.003 | 8.17E-39 |
|  |  |  |  |  | EPA 20:5n3 | -0.0334 | 0.0053 | 4.06E-10 |
|  |  |  |  |  | Plasma docosahexaenoic acid levels | NA | NA | 3.94E-14 |
|  |  |  |  |  | Plasma docosapentaenoic acid levels | NA | NA | 8.17E-39 |
|  |  |  |  |  | Plasma eicosapentaenoic acid levels | NA | NA | 4.06E-10 |
|  |  |  |  |  | Red blood cell fatty acid levels | -0.115 | 0.01954 | 4.00E-09 |
| Red blood cell DPA-n3 | rs174549 | chr11:61571382 | A | G | Eosinophil count | -0.02703 | 0.003846 | 2.10E-12 |
|  |  |  |  |  | Granulocyte count | -0.02896 | 0.003872 | 7.54E-14 |
|  |  |  |  |  | Granulocyte percentage of myeloid white cells | -0.03259 | 0.003853 | 2.71E-17 |
|  |  |  |  |  | Hematocrit | 0.023 | 0.003808 | 1.54E-09 |
|  |  |  |  |  | Hemoglobin concentration | 0.02697 | 0.003825 | 1.77E-12 |
|  |  |  |  |  | Mean corpuscular volume | -0.02357 | 0.003808 | 6.03E-10 |
|  |  |  |  |  | Mean platelet volume | -0.04643 | 0.003898 | 1.05E-32 |
|  |  |  |  |  | Monocyte percentage of white cells | 0.03014 | 0.003843 | 4.43E-15 |
|  |  |  |  |  | Myeloid white cell count | -0.02637 | 0.003883 | 1.10E-11 |
|  |  |  |  |  | Neutrophil count | -0.0261 | 0.003863 | 1.42E-11 |
|  |  |  |  |  | Platelet count | 0.03859 | 0.003931 | 9.47E-23 |
|  |  |  |  |  | Red blood cell count | 0.03337 | 0.003836 | 3.35E-18 |
|  |  |  |  |  | Red cell distribution width | -0.05227 | 0.003817 | 1.07E-42 |
|  |  |  |  |  | Sum basophil neutrophil counts | -0.02636 | 0.00387 | 9.70E-12 |
|  |  |  |  |  | Sum eosinophil basophil counts | -0.02522 | 0.00385 | 5.68E-11 |
|  |  |  |  |  | Sum neutrophil eosinophil counts | -0.02883 | 0.003866 | 8.79E-14 |
|  |  |  |  |  | White blood cell count | -0.02525 | 0.003866 | 6.47E-11 |
|  |  |  |  |  | ALA 18:3n3 | 0.0159 | 0.001 | 1.88E-58 |
|  |  |  |  |  | Adrenic acid 22:4n6 | -0.0478 | 0.002 | 5.94E-128 |
|  |  |  |  |  | Arachidonic acid 20:4n6 | -1.688 | 0.0265 | 1.24e-884 |
|  |  |  |  |  | Cis trans-18:2 | 0.0035 | 4.00E-04 | 4.77E-15 |
|  |  |  |  |  | Dihomo-gamma-linolenic acid | 0.3907 | 0.0141 | 4.79E-168 |
|  |  |  |  |  | Gamma-linolenic acid 18:3n6 | -0.0151 | 9.00E-04 | 3.98E-62 |
|  |  |  |  |  | Linoleic acid 18:2n6 | 1.427 | 0.0439 | 3.88E-232 |
|  |  |  |  |  | Oleic acid 18:1n9 | 0.2373 | 0.0205 | 4.31E-31 |
|  |  |  |  |  | Palmitoleic acid 16:1n7 | 0.0235 | 0.0035 | 2.31E-11 |
|  |  |  |  |  | Stearic acid 18:0 | -0.1757 | 0.0198 | 6.57E-19 |
|  |  |  |  |  | High density lipoprotein | -0.0362 | 0.0036 | 4.34E-23 |
|  |  |  |  |  | Low density lipoprotein | -0.05 | 0.0039 | 4.39E-36 |
|  |  |  |  |  | Total cholesterol | -0.0474 | 0.0038 | 2.05E-34 |
|  |  |  |  |  | Triglycerides | 0.0436 | 0.0035 | 6.92E-34 |
|  |  |  |  |  | FADS1 gene expression in liver | NA | NA | 2.10E-17 |
|  |  |  |  |  | Fasting blood glucose | NA | NA | 3.96E-09 |
|  |  |  |  |  | HDL cholesterol | NA | NA | 1.63E-08 |
|  |  |  |  |  | Heart rate | NA | NA | 1.38E-22 |
|  |  |  |  |  | Heart rate in non diabetics T2D | NA | NA | 6.10E-10 |
|  |  |  |  |  | Heart rate in non diabetics T2D with additional exclusion of MI HF Afib Heart block heart rate altering medications extremes of HR | NA | NA | 2.30E-08 |
|  |  |  |  |  | Heart rate in non hypertensives | NA | NA | 1.90E-10 |
|  |  |  |  |  | Heart rate in non hypertensives with additional exclusion of MI HF Afib Heart block heart rate altering medications extremes of HR | NA | NA | 5.00E-09 |
|  |  |  |  |  | Heart rate in non obese non cancer and non kidney stone individuals | NA | NA | 1.20E-09 |
|  |  |  |  |  | Heart rate in non obese non cancer and non kidney stone individuals with additional exclusion of MI HF Afib Heart block heart rate altering medications extremes of HR | NA | NA | 1.60E-08 |
|  |  |  |  |  | Heart rate with additional exclusion of MI HF Afib Heart block heart rate altering medications extremes of HR | NA | NA | 2.80E-09 |
|  |  |  |  |  | Heart rate with age12 | NA | NA | 1.50E-08 |
|  |  |  |  |  | LDL cholesterol | NA | NA | 5.27E-20 |
|  |  |  |  |  | Lung cancer | NA | NA | 7.60E-13 |
|  |  |  |  |  | Plasma alpha linolenic acid levels | NA | NA | 1.88E-58 |
|  |  |  |  |  | Plasma oleic acid | NA | NA | 4.31E-31 |
|  |  |  |  |  | Plasma palmitoleic acid | NA | NA | 2.31E-11 |
|  |  |  |  |  | Plasma stearic acid | NA | NA | 6.57E-19 |
|  |  |  |  |  | QT interval | NA | NA | 4.33E-08 |
|  |  |  |  |  | RR interval | NA | NA | 2.69E-09 |
|  |  |  |  |  | Fasting glucose | -0.019 | 0.0023 | 1.06E-17 |
|  |  |  |  |  | Eosinophil counts | 0.02703 | 0.003842 | 2.00E-12 |
|  |  |  |  |  | Laryngeal squamous cell carcinoma | NA | NA | 1.00E-20 |
|  |  |  |  |  | Metabolite levels | NA | NA | 2.00E-30 |
|  |  |  |  |  | Trans fatty acid levels | 0.0035 | 0.0004472 | 5.00E-15 |
|  |  |  |  |  | Asthma | -0.005078 | 0.0008413 | 1.58E-09 |
|  |  |  |  |  | Heel bone mineral density right | 0.0246 | 0.004472 | 3.80E-08 |
|  |  |  |  |  | Height | -0.01354 | 0.001866 | 3.95E-13 |
|  |  |  |  |  | Nap during day | 0.01002 | 0.001557 | 1.21E-10 |
|  |  |  |  |  | Pulse rate | 0.03262 | 0.002705 | 1.79E-33 |
|  |  |  |  |  | Self-reported asthma | -0.005339 | 0.0008428 | 2.38E-10 |
| Plasma DPA-n3 | rs780094 | chr2:27741237 | T | C | Granulocyte count | 0.02779 | 0.003675 | 3.95E-14 |
|  |  |  |  |  | Granulocyte percentage of myeloid white cells | 0.0393 | 0.003655 | 5.72E-27 |
|  |  |  |  |  | Hematocrit | -0.02212 | 0.003612 | 9.12E-10 |
|  |  |  |  |  | High light scatter percentage of red cells | 0.03392 | 0.003668 | 2.32E-20 |
|  |  |  |  |  | High light scatter reticulocyte count | 0.03077 | 0.003668 | 4.98E-17 |
|  |  |  |  |  | Lymphocyte count | 0.02236 | 0.003683 | 1.27E-09 |
|  |  |  |  |  | Monocyte percentage of white cells | -0.04437 | 0.003643 | 4.02E-34 |
|  |  |  |  |  | Myeloid white cell count | 0.02465 | 0.003684 | 2.22E-11 |
|  |  |  |  |  | Neutrophil count | 0.02933 | 0.003665 | 1.22E-15 |
|  |  |  |  |  | Platelet count | 0.03571 | 0.003728 | 9.71E-22 |
|  |  |  |  |  | Plateletcrit | 0.03368 | 0.003742 | 2.25E-19 |
|  |  |  |  |  | Red blood cell count | -0.02097 | 0.003636 | 8.01E-09 |
|  |  |  |  |  | Red cell distribution width | -0.02401 | 0.003623 | 3.39E-11 |
|  |  |  |  |  | Reticulocyte count | 0.031 | 0.003674 | 3.23E-17 |
|  |  |  |  |  | Reticulocyte fraction of red cells | 0.03648 | 0.003672 | 2.93E-23 |
|  |  |  |  |  | Sum basophil neutrophil counts | 0.02871 | 0.003671 | 5.24E-15 |
|  |  |  |  |  | Sum neutrophil eosinophil counts | 0.02831 | 0.003668 | 1.17E-14 |
|  |  |  |  |  | White blood cell count | 0.0295 | 0.003667 | 8.65E-16 |
|  |  |  |  |  | DPA 22:5n3 | 0.0167 | 0.0029 | 9.04E-09 |
|  |  |  |  |  | Palmitoleic acid 16:1n7 | 0.0199 | 0.0033 | 1.26E-09 |
|  |  |  |  |  | log eGFR creatinine in non diabetics | 0.0063 | 0.00092 | 4.80E-12 |
|  |  |  |  |  | log eGFR creatinine | NA | NA | 2.40E-09 |
|  |  |  |  |  | Type II diabetes | -0.08066 | 0.01318 | 8.50E-10 |
|  |  |  |  |  | Height | -0.021 | 0.003 | 5.70E-12 |
|  |  |  |  |  | Total cholesterol | 0.0504 | 0.0036 | 5.28E-41 |
|  |  |  |  |  | Triglycerides | 0.1102 | 0.0034 | 2.65E-220 |
|  |  |  |  |  | 2 hour glucose tolerance test | NA | NA | 1.04E-10 |
|  |  |  |  |  | APOB assay lipoprotein fraction concentration | NA | NA | 1.07E-17 |
|  |  |  |  |  | APOB assay lipoprotein fraction concentration in fasting sample | NA | NA | 2.67E-13 |
|  |  |  |  |  | C reactive protein | NA | NA | 3.90E-08 |
|  |  |  |  |  | C reactive protein female | NA | NA | 2.74E-32 |
|  |  |  |  |  | Fasting blood glucose | NA | NA | 5.60E-38 |
|  |  |  |  |  | Fasting blood glucose in high BMI subjects | NA | NA | 2.54E-17 |
|  |  |  |  |  | Fasting blood glucose in low BMI subjects | NA | NA | 6.94E-14 |
|  |  |  |  |  | Fasting insulin | NA | NA | 3.60E-20 |
|  |  |  |  |  | Fasting insulin in high BMI subjects | NA | NA | 3.96E-09 |
|  |  |  |  |  | Fasting plasma glucose after exclusion of subjects with FG levels 7 mmolL | NA | NA | 8.50E-09 |
|  |  |  |  |  | HDL cholesterol mean size lipoprotein fraction concentration in fasting sample | NA | NA | 1.70E-09 |
|  |  |  |  |  | HDL cholesterol small lipoprotein fraction concentration in fasting sample | NA | NA | 3.10E-17 |
|  |  |  |  |  | HOMA IR | NA | NA | 3.00E-24 |
|  |  |  |  |  | Height adults | NA | NA | 2.20E-11 |
|  |  |  |  |  | Hypertriglyceridemia | NA | NA | 5.29E-13 |
|  |  |  |  |  | IDL total lipoprotein fraction concentration | NA | NA | 6.98E-10 |
|  |  |  |  |  | IDL total lipoprotein fraction concentration in fasting sample | NA | NA | 4.50E-08 |
|  |  |  |  |  | IFT172 cis expression in liver | NA | NA | 1.53E-27 |
|  |  |  |  |  | IFT172 cis expression in omental fat | NA | NA | 1.70E-63 |
|  |  |  |  |  | IFT172 cis expression in subcutaneous fat | NA | NA | 8.85E-52 |
|  |  |  |  |  | LDL cholesterol mean size lipoprotein fraction concentration | NA | NA | 3.30E-12 |
|  |  |  |  |  | LDL cholesterol mean size lipoprotein fraction concentration in fasting sample | NA | NA | 9.80E-13 |
|  |  |  |  |  | LDL cholesterol small lipoprotein fraction concentration | NA | NA | 2.60E-19 |
|  |  |  |  |  | LDL cholesterol small lipoprotein fraction concentration in fasting sample | NA | NA | 8.60E-18 |
|  |  |  |  |  | LDL cholesterol total lipoprotein fraction concentration | NA | NA | 3.79E-19 |
|  |  |  |  |  | LDL cholesterol total lipoprotein fraction concentration in fasting sample | NA | NA | 2.87E-16 |
|  |  |  |  |  | Nonalcoholic fatty liver disease | NA | NA | 2.59E-08 |
|  |  |  |  |  | Plasma C reactive protein female | NA | NA | 6.73E-15 |
|  |  |  |  |  | Plasma docosapentaenoic acid levels | NA | NA | 2.52E-09 |
|  |  |  |  |  | Plasma palmitoleic acid | NA | NA | 1.26E-09 |
|  |  |  |  |  | Plasma protein C levels | NA | NA | 1.89E-16 |
|  |  |  |  |  | Serum creatinine | NA | NA | 9.60E-11 |
|  |  |  |  |  | Serum triglycerides | NA | NA | 8.05E-14 |
|  |  |  |  |  | Serum urate | NA | NA | 4.68E-17 |
|  |  |  |  |  | Triglycerides change with statins | NA | NA | 1.20E-14 |
|  |  |  |  |  | Triglycerides in metabolic syndrome subjects without diabetes | NA | NA | 5.89E-20 |
|  |  |  |  |  | Triglycerides ln | NA | NA | 1.50E-15 |
|  |  |  |  |  | Triglycerides mmoll | NA | NA | 5.03E-09 |
|  |  |  |  |  | Type 2 diabetes | NA | NA | 1.30E-09 |
|  |  |  |  |  | Uric acid | NA | NA | 1.40E-09 |
|  |  |  |  |  | VLDL cholesterol total lipoprotein fraction concentration in fasting sample | NA | NA | 1.56E-13 |
|  |  |  |  |  | Fasting glucose | -0.027 | 0.0021 | 2.58E-37 |
|  |  |  |  |  | log Fasting insulin | -0.019 | 0.0025 | 7.06E-14 |
|  |  |  |  |  | log Fasting insulin adjusted for BMI | -0.019 | 0.0021 | 7.37E-20 |
|  |  |  |  |  | Age related disease endophenotypes | NA | NA | 8.00E-101 |
|  |  |  |  |  | Age related diseases mortality and associated endophenotypes | NA | NA | 4.00E-91 |
|  |  |  |  |  | Calcium levels | 0.017 | 0.002629 | 1.00E-10 |
|  |  |  |  |  | Crohns disease | NA | NA | 4.00E-22 |
|  |  |  |  |  | Fasting glucose related traits | NA | NA | 6.00E-38 |
|  |  |  |  |  | Fasting glucose related traits interaction with BMI | NA | NA | 4.00E-24 |
|  |  |  |  |  | Fasting insulin related traits | NA | NA | 4.00E-20 |
|  |  |  |  |  | Fasting insulin related traits interaction with BMI | NA | NA | 3.00E-10 |
|  |  |  |  |  | Fasting plasma glucose | NA | NA | 4.00E-09 |
|  |  |  |  |  | Gondoic acid 201n 9 levels | 0.002 | 0.0003609 | 3.00E-08 |
|  |  |  |  |  | Metabolic syndrome | 0.13 | 0.01422 | 6.00E-20 |
|  |  |  |  |  | Metabolic traits | 0.101 | 0.006595 | 6.00E-53 |
|  |  |  |  |  | Phospholipid levels plasma | 0.02 | 0.003479 | 9.00E-09 |
|  |  |  |  |  | Renal underexcretion gout | 0.3001 | 0.05004 | 2.00E-09 |
|  |  |  |  |  | Triglyceride levels | 0.1 | 0.008807 | 7.00E-30 |
|  |  |  |  |  | Urate levels in obese individuals | -0.085 | 0.01515 | 2.00E-08 |
|  |  |  |  |  | Uric acid levels | 0.05 | 0.008184 | 1.00E-09 |
|  |  |  |  |  | Alcohol intake frequency | 0.04817 | 0.003654 | 1.13E-39 |
|  |  |  |  |  | Alcohol intake versus 10 years previously | 0.01213 | 0.001899 | 1.65E-10 |
|  |  |  |  |  | Arm fat-free mass left | -0.0187 | 0.001588 | 5.30E-32 |
|  |  |  |  |  | Arm fat-free mass right | -0.01918 | 0.001556 | 7.08E-35 |
|  |  |  |  |  | Arm predicted mass left | -0.01853 | 0.001582 | 1.12E-31 |
|  |  |  |  |  | Arm predicted mass right | -0.01904 | 0.001551 | 1.22E-34 |
|  |  |  |  |  | Basal metabolic rate | -0.01875 | 0.001656 | 1.05E-29 |
|  |  |  |  |  | Comparative height size at age 10 | -0.01443 | 0.001706 | 2.73E-17 |
|  |  |  |  |  | Daytime dozing or sleeping | 0.007992 | 0.001236 | 1.00E-10 |
|  |  |  |  |  | Diabetes diagnosed by doctor | -0.003732 | 0.0005357 | 3.25E-12 |
|  |  |  |  |  | Impedance of arm left | 0.01849 | 0.001764 | 1.09E-25 |
|  |  |  |  |  | Impedance of arm right | 0.01826 | 0.001757 | 2.65E-25 |
|  |  |  |  |  | Impedance of leg left | 0.01825 | 0.002278 | 1.11E-15 |
|  |  |  |  |  | Impedance of leg right | 0.01786 | 0.00226 | 2.75E-15 |
|  |  |  |  |  | Impedance of whole body | 0.01996 | 0.001905 | 1.10E-25 |
|  |  |  |  |  | Leg fat-free mass left | -0.01689 | 0.001645 | 1.01E-24 |
|  |  |  |  |  | Leg fat-free mass right | -0.01687 | 0.001644 | 1.10E-24 |
|  |  |  |  |  | Leg predicted mass left | -0.0167 | 0.001634 | 1.59E-24 |
|  |  |  |  |  | Leg predicted mass right | -0.01682 | 0.001634 | 7.45E-25 |
|  |  |  |  |  | Medication for cholesterol, blood pressure or diabetes: cholesterol lowering medication | 0.01126 | 0.00156 | 5.27E-13 |
|  |  |  |  |  | Pulse rate | 0.02764 | 0.002574 | 6.81E-27 |
|  |  |  |  |  | Self-reported diabetes | -0.002996 | 0.0004858 | 6.93E-10 |
|  |  |  |  |  | Self-reported gout | 0.003201 | 0.0002953 | 2.27E-27 |
|  |  |  |  |  | Self-reported high cholesterol | 0.009575 | 0.0008188 | 1.39E-31 |
|  |  |  |  |  | Sitting height | -0.01796 | 0.001931 | 1.42E-20 |
|  |  |  |  |  | Sodium in urine | 0.01908 | 0.002433 | 4.41E-15 |
|  |  |  |  |  | Treatment with allopurinol | 0.002657 | 0.000264 | 7.90E-24 |
|  |  |  |  |  | Treatment with atorvastatin | 0.002956 | 0.0004279 | 4.96E-12 |
|  |  |  |  |  | Treatment with cholesterol lowering medication | 0.007841 | 0.001138 | 5.72E-12 |
|  |  |  |  |  | Treatment with simvastatin | 0.004384 | 0.0007923 | 3.16E-08 |
|  |  |  |  |  | Trunk fat-free mass | -0.02057 | 0.001573 | 4.92E-39 |
|  |  |  |  |  | Trunk predicted mass | -0.02057 | 0.001568 | 2.64E-39 |
|  |  |  |  |  | Weight | -0.01651 | 0.002183 | 4.04E-14 |
|  |  |  |  |  | Whole body fat-free mass | -0.0197 | 0.00158 | 1.06E-35 |
|  |  |  |  |  | Whole body water mass | -0.01995 | 0.001582 | 1.93E-36 |
|  |  |  |  |  | Age at menopause | 0.12 | 0.02 | 1.50E-09 |
|  |  |  |  |  | Glucose tolerance test | NA | NA | 6.00E-38 |
|  |  |  |  |  | Glucose transporter type 2 | NA | NA | 4.00E-20 |
|  |  |  |  |  | Insulin resistance | NA | NA | 3.00E-24 |
|  |  |  |  |  | Metabolic syndrome x | NA | NA | 6.00E-20 |
|  |  |  |  |  | Metabolism | NA | NA | 6.00E-53 |
|  |  |  |  |  | Phospholipids | NA | NA | 9.00E-09 |
| Plasma DPA-n3 | rs3734398 | chr6:10982973 | C | T | DHA 22:6n3 | -0.1143 | 0.0144 | 1.65E-15 |
|  |  |  |  |  | EPA 20:5n3 | 0.0352 | 0.0051 | 3.99E-12 |
|  |  |  |  |  | Plasma docosahexaenoic acid levels | NA | NA | 1.65E-15 |
|  |  |  |  |  | Plasma eicosapentaenoic acid levels | NA | NA | 3.99E-12 |

PUFA, polyunsaturated fatty acid; AA, arachidonic acid; DPA-n3, docosapentaenoic acid; SNP, single nucleotide polymorphism; Chr, chromosome; EAF, Effect allele frequency; SE, standard error. For each PUFA, we selected SNPs that reached genome-wide significance level (P < 5 × 10^−8^) and were restricted by linkage disequilibrium (LD) clumping to ensure independence (R^2^ < 0.001 within a 10 Mb window).

**Reference**

1. Zhao JV, Schooling CM. Effect of Linoleic Acid on Ischemic Heart Disease and Its Risk Factors: A Mendelian Randomization Study. *BMC Med* (2019) 17(1):61. Epub 2019/03/15. doi: 10.1186/s12916-019-1293-x.

2. Zhang T, Au Yeung SL, Schooling CM. Associations of Arachidonic Acid Synthesis with Cardiovascular Risk Factors and Relation to Ischemic Heart Disease and Stroke: A Univariable and Multivariable Mendelian Randomization Study. *Nutrients* (2021) 13(5). Epub 2021/05/01. doi: 10.3390/nu13051489.

3. Yuan S, Back M, Bruzelius M, Mason AM, Burgess S, Larsson S. Plasma Phospholipid Fatty Acids, Fads1 and Risk of 15 Cardiovascular Diseases: A Mendelian Randomisation Study. *Nutrients* (2019) 11(12). Epub 2019/12/11. doi: 10.3390/nu11123001.

4. Yuan T, Si S, Li Y, Li W, Chen X, Liu C, et al. Roles for Circulating Polyunsaturated Fatty Acids in Ischemic Stroke and Modifiable Factors: A Mendelian Randomization Study. *Nutr J* (2020) 19(1):70. Epub 2020/07/13. doi: 10.1186/s12937-020-00582-4.

5. Liao LZ, Li WD, Liu Y, Li JP, Zhuang XD, Liao XX. Exploring the Causal Pathway from Omega-6 Levels to Coronary Heart Disease: A Network Mendelian Randomization Study. *Nutr Metab Cardiovasc Dis* (2020) 30(2):233-40. Epub 2019/10/28. doi: 10.1016/j.numecd.2019.09.013.

6. Mazidi M, Katsiki N, Shekoohi N, Banach M. Monounsaturated Fatty Acid Levels May Not Affect Cardiovascular Events: Results from a Mendelian Randomization Analysis. *Front Nutr* (2020) 7:123. Epub 2020/09/29. doi: 10.3389/fnut.2020.00123.

7. Zhang T, Zhao JV, Schooling CM. The Associations of Plasma Phospholipid Arachidonic Acid with Cardiovascular Diseases: A Mendelian Randomization Study. *EBioMedicine* (2021) 63:103189. Epub 2021/01/09. doi: 10.1016/j.ebiom.2020.103189.

8. Yuan S, Larsson SC. Plasma Phospholipid Fatty Acids and Risk of Atrial Fibrillation: A Mendelian Randomization Study. *Nutrients* (2019) 11(7). Epub 2019/07/25. doi: 10.3390/nu11071651.

9. Park S, Lee S, Kim Y, Lee Y, Kang M, Kim K, et al. Causal Effects of Serum Levels of N-3 or N-6 Polyunsaturated Fatty Acids on Coronary Artery Disease: Mendelian Randomization Study. *Nutrients* (2021) 13(5). Epub 2021/05/01. doi: 10.3390/nu13051490.

10. Wang W, Wang J, Zhuang Z, Gao M, Yang R, Liu Z, et al. Assessment of Causality between Modifiable Factors and Heart Failure: A Mendelian Randomization Analysis. *Asia Pac J Clin Nutr* (2021) 30(2):340-7. Epub 2021/07/01. doi: 10.6133/apjcn.202106_30(2).0019.

11. Chen HY, Cairns BJ, Small AM, Burr HA, Ambikkumar A, Martinsson A, et al. Association of Fads1/2 Locus Variants and Polyunsaturated Fatty Acids with Aortic Stenosis. *JAMA Cardiol* (2020) 5(6):694-702. Epub 2020/03/19. doi: 10.1001/jamacardio.2020.0246.

12. Yuan S, Larsson SC. Association of Genetic Variants Related to Plasma Fatty Acids with Type 2 Diabetes Mellitus and Glycaemic Traits: A Mendelian Randomisation Study. *Diabetologia* (2020) 63(1):116-23. Epub 2019/11/07. doi: 10.1007/s00125-019-05019-0.

13. Jager S, Cuadrat R, Hoffmann P, Wittenbecher C, Schulze MB. Desaturase Activity and the Risk of Type 2 Diabetes and Coronary Artery Disease: A Mendelian Randomization Study. *Nutrients* (2020) 12(8). Epub 2020/08/01. doi: 10.3390/nu12082261.

14. Adams CD, Neuhausen SL. Evaluating Causal Associations between Chronotype and Fatty Acids and between Fatty Acids and Type 2 Diabetes: A Mendelian Randomization Study. *Nutr Metab Cardiovasc Dis* (2019) 29(11):1176-84. Epub 2019/08/06. doi: 10.1016/j.numecd.2019.06.020.

15. Zulyniak MA, Fuller H, Iles MM. Investigation of the Causal Association between Long-Chain N-6 Polyunsaturated Fatty Acid Synthesis and the Risk of Type 2 Diabetes: A Mendelian Randomization Analysis. *Lifestyle Genom* (2020) 13(5):146-53. Epub 2020/08/14. doi: 10.1159/000509663.

16. Ma M, Yang F, Wang Z, Bao Q, Shen J, Xie X. Association of Plasma Polyunsaturated Fatty Acids with Arterial Blood Pressure: A Mendelian Randomization Study. *Medicine (Baltimore)* (2021) 100(3):e24359. Epub 2021/02/07. doi: 10.1097/MD.0000000000024359.

17. Zhao JV, Schooling CM. The Role of Linoleic Acid in Asthma and Inflammatory Markers: A Mendelian Randomization Study. *Am J Clin Nutr* (2019) 110(3):685-90. Epub 2019/07/10. doi: 10.1093/ajcn/nqz130.

18. Liyanage UE, Ong JS, An J, Gharahkhani P, Law MH, MacGregor S. Mendelian Randomization Study for Genetically Predicted Polyunsaturated Fatty Acids Levels on Overall Cancer Risk and Mortality. *Cancer Epidemiol Biomarkers Prev* (2019) 28(6):1015-23. Epub 2019/04/06. doi: 10.1158/1055-9965.EPI-18-0940.

19. Larsson SC, Carter P, Vithayathil M, Mason AM, Michaelsson K, Baron JA, et al. Genetically Predicted Plasma Phospholipid Arachidonic Acid Concentrations and 10 Site-Specific Cancers in Uk Biobank and Genetic Consortia Participants: A Mendelian Randomization Study. *Clin Nutr* (2021) 40(5):3332-7. Epub 2020/11/18. doi: 10.1016/j.clnu.2020.11.004.

20. Liu J, Zhou H, Zhang Y, Huang Y, Fang W, Yang Y, et al. Docosapentaenoic Acid and Lung Cancer Risk: A Mendelian Randomization Study. *Cancer Med* (2019) 8(4):1817-25. Epub 2019/02/12. doi: 10.1002/cam4.2018.

21. Shen J, Zhou H, Liu J, Zhang Y, Zhou T, Yang Y, et al. A Modifiable Risk Factors Atlas of Lung Cancer: A Mendelian Randomization Study. *Cancer Med* (2021) 10(13):4587-603. Epub 2021/06/03. doi: 10.1002/cam4.4015.

22. Yang Z, Li J, Sun Y, Qu Z, Lin Y, Zhang L, et al. Using Genetic Variants to Evaluate the Causal Effect of Plasma Phospholipid Fatty Acids on Breast Cancer and Prostate Cancer: A Mendelian Randomization Study. *Front Genet* (2021) 12:664498. Epub 2021/07/20. doi: 10.3389/fgene.2021.664498.

23. Guo F, Wang M, Guo X, Pu L, Sun M, Li S, et al. The Association between Fatty Acid Intake and Breast Cancer Based on the Nhanes and Mendelian Randomization Study. *Cancer Epidemiol* (2021) 73:101966. Epub 2021/06/20. doi: 10.1016/j.canep.2021.101966.

24. Khankari NK, Murff HJ, Zeng C, Wen W, Eeles RA, Easton DF, et al. Polyunsaturated Fatty Acids and Prostate Cancer Risk: A Mendelian Randomisation Analysis from the Practical Consortium. *Br J Cancer* (2016) 115(5):624-31. Epub 2016/08/05. doi: 10.1038/bjc.2016.228.

25. Ghoneim DH, Zhu J, Zheng W, Long J, Murff HJ, Ye F, et al. Mendelian Randomization Analysis of N-6 Polyunsaturated Fatty Acid Levels and Pancreatic Cancer Risk. *Cancer Epidemiol Biomarkers Prev* (2020) 29(12):2735-9. Epub 2020/09/25. doi: 10.1158/1055-9965.EPI-20-0651.

26. Seviiri M, Law MH, Ong JS, Gharahkhani P, Nyholt DR, Olsen CM, et al. Polyunsaturated Fatty Acid Levels and the Risk of Keratinocyte Cancer: A Mendelian Randomization Analysis. *Cancer Epidemiol Biomarkers Prev* (2021) 30(8):1591-8. Epub 2021/06/06. doi: 10.1158/1055-9965.EPI-20-1765.

27. Khankari NK, Banbury BL, Borges MC, Haycock P, Albanes D, Arndt V, et al. Mendelian Randomization of Circulating Polyunsaturated Fatty Acids and Colorectal Cancer Risk. *Cancer Epidemiol Biomarkers Prev* (2020) 29(4):860-70. Epub 2020/02/14. doi: 10.1158/1055-9965.EPI-19-0891.

28. May-Wilson S, Sud A, Law PJ, Palin K, Tuupanen S, Gylfe A, et al. Pro-Inflammatory Fatty Acid Profile and Colorectal Cancer Risk: A Mendelian Randomisation Analysis. *Eur J Cancer* (2017) 84:228-38. Epub 2017/08/23. doi: 10.1016/j.ejca.2017.07.034.

29. Isom CA, Shrubsole MJ, Cai Q, Smalley WE, Ness RM, Zheng W, et al. Arachidonic Acid and Colorectal Adenoma Risk: A Mendelian Randomization Study. *Clin Epidemiol* (2019) 11:17-22. Epub 2018/12/28. doi: 10.2147/CLEP.S186883.

30. Liyanage UE, Law MH, Ong JS, Cust AE, Mann GJ, Ward SV, et al. Polyunsaturated Fatty Acids and Risk of Melanoma: A Mendelian Randomisation Analysis. *Int J Cancer* (2018) 143(3):508-14. Epub 2018/02/24. doi: 10.1002/ijc.31334.

31. Saunders CN, Cornish AJ, Kinnersley B, Law PJ, Claus EB, Il'yasova D, et al. Lack of Association between Modifiable Exposures and Glioma Risk: A Mendelian Randomization Analysis. *Neuro Oncol* (2020) 22(2):207-15. Epub 2019/10/31. doi: 10.1093/neuonc/noz209.

32. Yuan S, Lemming EW, Michaelsson K, Larsson SC. Plasma Phospholipid Fatty Acids, Bone Mineral Density and Fracture Risk: Evidence from a Mendelian Randomization Study. *Clin Nutr* (2020) 39(7):2180-6. Epub 2019/10/01. doi: 10.1016/j.clnu.2019.09.005.

33. Zhao JV, Schooling CM. Role of Linoleic Acid in Autoimmune Disorders: A Mendelian Randomisation Study. *Ann Rheum Dis* (2019) 78(5):711-3. Epub 2018/11/10. doi: 10.1136/annrheumdis-2018-214519.

34. Sun L, Zhu J, Mi S, Li Y, Wang T, Li Y. Causal Association of Monounsaturated Fatty Acids with Rheumatoid Arthritis but Not Osteoarthritis: A Two-Sample Mendelian Randomization Study. *Nutrition* (2021) 91-92:111363. Epub 2021/07/12. doi: 10.1016/j.nut.2021.111363.

35. Wang K, Zhong Y, Yang F, Hu C, Liu X, Zhu Y, et al. Causal Effects of N-6 Polyunsaturated Fatty Acids on Age-Related Macular Degeneration: A Mendelian Randomization Study. *J Clin Endocrinol Metab* (2021) 106(9):e3565-e72. Epub 2021/05/14. doi: 10.1210/clinem/dgab338.

36. Carreras-Torres R, Ibanez-Sanz G, Obon-Santacana M, Duell EJ, Moreno V. Identifying Environmental Risk Factors for Inflammatory Bowel Diseases: A Mendelian Randomization Study. *Sci Rep* (2020) 10(1):19273. Epub 2020/11/08. doi: 10.1038/s41598-020-76361-2.

37. Cheng TS, Day FR, Perry JRB, Luan J, Langenberg C, Forouhi NG, et al. Prepubertal Dietary and Plasma Phospholipid Fatty Acids Related to Puberty Timing: Longitudinal Cohort and Mendelian Randomization Analyses. *Nutrients* (2021) 13(6). Epub 2021/06/03. doi: 10.3390/nu13061868.

38. Milaneschi Y, Peyrot WJ, Nivard MG, Mbarek H, Boomsma DI, B WJHP. A Role for Vitamin D and Omega-3 Fatty Acids in Major Depression? An Exploration Using Genomics. *Transl Psychiatry* (2019) 9(1):219. Epub 2019/09/07. doi: 10.1038/s41398-019-0554-y.

39. Tomata Y, Larsson SC, Hagg S. Polyunsaturated Fatty Acids and Risk of Alzheimer's Disease: A Mendelian Randomization Study. *Eur J Nutr* (2020) 59(4):1763-6. Epub 2019/11/05. doi: 10.1007/s00394-019-02126-x.

40. Wang Z, Meng L, Shen L, Ji HF. Impact of Modifiable Risk Factors on Alzheimer's Disease: A Two-Sample Mendelian Randomization Study. *Neurobiol Aging* (2020) 91:167 e11- e19. Epub 2020/03/25. doi: 10.1016/j.neurobiolaging.2020.02.018.

41. Jones HJ, Borges MC, Carnegie R, Mongan D, Rogers PJ, Lewis SJ, et al. Associations between Plasma Fatty Acid Concentrations and Schizophrenia: A Two-Sample Mendelian Randomisation Study. *Lancet Psychiatry* (2021). Epub 2021/11/05. doi: 10.1016/S2215-0366(21)00286-8.

42. Sallis H, Steer C, Paternoster L, Davey Smith G, Evans J. Perinatal Depression and Omega-3 Fatty Acids: A Mendelian Randomisation Study. *J Affect Disord* (2014) 166:124-31. Epub 2014/07/12. doi: 10.1016/j.jad.2014.04.077.

43. Thompson AD, Jones HJ, Heron J, Hibbeln J, Sullivan S, Zammit S. Omega-3 and Omega-6 Fatty Acids and Risk of Psychotic Outcomes in the Alspac Birth Cohort. *Schizophr Res* (2020) 224:108-15. Epub 2020/10/18. doi: 10.1016/j.schres.2020.09.018.
